# Effect of berry maturity stages on the germination and protein constituents of African nightshade (*Solanum scabrum)* seeds

**DOI:** 10.1038/s41598-024-80312-6

**Published:** 2024-12-16

**Authors:** Noella Andenyi Ekhuya, Mary Abukutsa Onyango, Jennifer Senkler, Traud Winkelmann, Christin Bündig

**Affiliations:** 1 Department of Horticulture and Food Security, Jomo Kenyatta University of Agriculture and Technology, P.O. Box 62000, 0200 Nairobi, Kenya; 2 Institute of Plant Genetics, Leibniz Universität Hannover, Herrenhäuser Straße 2, 30419 Hannover, Germany; 3 Institute of Horticultural Production Systems, Leibniz Universität Hannover, Herrenhäuser Straße 2, 30419 Hannover, Germany

**Keywords:** Germination percentage, Seed quality, Proteomics, *Solanum scabrum*, Biological techniques, Developmental biology

## Abstract

African nightshade (*Solanum scabrum*) is a vegetable of great importance in several African countries. Production by seed is constrained by limited access to high quality seed, leaving farmers unable to meet the growing demand. The aim of this study was to investigate effects of berry maturity stages (mature green and purple) on germination and protein components of African nightshade seeds. Nine accessions were used for the germination assays and three selected for the proteomics. The seeds harvested at the purple ripe stage showed a higher germination percentage than seeds harvested at the mature green stage. Seeds of three accessions, which showed different germination responses depending on maturity, were subjected to gel-based proteome comparison followed by mass spectrometry. Analysis revealed a total of 563 spots, of which 19–23% were differentially abundant between the two harvest times. This is the first study to utilise proteomics in African nightshade. Four types of seed storage proteins were identified. Identified proteins were associated with seed maturation, indicating more advanced maturation stages in accessions with higher germination capacity. In addition, a reference proteome map of *S. scabrum* was created using GelMap platform (www.gelmap.de). Colour change of African nightshade berries seems to be a suitable indicator for seed maturity.

**Supplementary Information:**

The online version contains supplementary material available at 10.1038/s41598-024-80312-6.

## Introduction

African nightshades are indigenous leafy vegetables that are commonly consumed in West and East Africa. Several species are known under African nightshades and occur in the *Solanum* genus within the *Solanaceae* family^1^. The most common species include *Solanum scabrum*, *S. villosum*, *S. nigrum*, and *S. americanum*^1^. Like many traditional vegetables, African nightshades play a significant role in nutrition, income generation and food security. Nightshade vegetables are a source of essential nutrients, as they are rich in calcium, iron, and vitamins A and C^2,3^. Similar results using the accession Olevolosi revealed that African nightshades are rich in health promoting phytonutrients and mineral elements^4^. Some of the mineral contents reported for Olevolosi of African nightshade harvested at 120 days after planting (dap) were phosphorus 3.2 g kg^− 1^, potassium 40.5 g kg^− 1^, calcium 21.6 g kg^− 1^, magnesium 3.7 g kg^− 1^, iron 0.7 g kg^− 1^, zinc 62.1 g kg^− 1^, manganese 287.5 g kg^− 1^ and nitrogen 58.6 g kg^− 1 [4^.

African nightshades are propagated through seeds. The genetic exchange between different individuals within the same species occurs by open pollination. After successful pollination, flowers develop into fruits after about 9–10 days. The berries then develop to maturity within 3–4 weeks. Within two weeks of reaching physiological maturity, the berries change colour, ripen and soften. However, the production of this vegetable has been largely constrained by limited access to high-quality seed stock^5^. Despite the growing popularity and demand for these vegetables, the production has not reached significant proportions, thus, farmers can hardly meet the growing demand. It has been previously shown that production stands at around 1.5 tonnes per hectare (t ha^− 1^) against a potential yield of 2.5 t ha^− 1^^6^. The use of high-quality seeds and good crop management practices will help to achieve optimal yields. However, the poor seed quality (laboratory germination percentage of around 50% and low field germination of only up to 5.3%) is majorly attributed to seed harvesting and processing techniques^3^. Factors affecting seed quality include the maturity stage at which the seed is harvested, methods of seed extraction, and storage conditions of the seeds^3^.

Seed maturity is a significant factor determining seed quality and is essential for germination and successful seedling emergence. For instance, in tomato it was shown that quality tomato seeds could be obtained from half-ripe as well as fully-ripe berry stages of different accessions based on colour change of the berries, leading to a high germination percentage and seedling emergence^7^. However, the seeds produced at the initial stage recorded germination percentages below the recommended, thus indicating lower seed quality. As a result of the accumulation of essential elements, storage reserves and nutrients in the seed, the constitution of seeds extracted from fruits at different stages of maturity are likely to vary, thereby affecting the seed quality^8^. For example, research on coffee seed development showed that seeds of *Coffea arabica* harvested at two different developmental stages presented a difference in their water-soluble proteins. These differences were shown to directly influence the germination of the coffee seeds hence their quality^9^. In tomatoes, an increase in germination rate and germination percentage was recorded as fruits developed from the breaker stage to red ripe stage^10^. On the other hand, a maximum germination percentage was obtained in seeds extracted from fruits of tomatoes at the breaker stage, while it decreased as the fruits continued to ripen. However, fruits at the red ripe stage germinated more rapidly, and further delayed harvesting led to a reduction in germination percentage^11^. Therefore, African nightshade seeds harvested at different maturity stages may have varying quality. Nevertheless, profound knowledge about the optimal maturity stage of African nightshade is lacking.

Proteomics is a powerful tool for monitoring the physiology of cells and tissues under specific developmental conditions. Different studies have already analysed seeds of multiple species from important crop plants through proteomics, usually under diverse environmental conditions^12^^13^ . Results by Li et al. (2012)^14^ revealed variations in abundance of protein levels at different stages of *Brassica campestris* L. seed development. A proteomic analysis of the change in the amount of stress-related proteins during seed development showed that some LEA proteins accumulated at physiological maturity and remained at high levels in mature seeds of *Oryza sativa*^15^.

This study aimed to determine the germination of African nightshade (*Solanum scabrum*) seeds extracted from berries at different maturity stages. Seeds of accessions which showed contrasting germination responses depending on their maturity were submitted to a gel-based proteomic comparison followed by mass spectrometry in order to identify physiological differences between the seeds of different maturity stages and between the contrasting accessions. This study sought to answer the following questions: How does germination of seeds of African nightshade harvested from berries at the mature green stage and the ripe stage differ? How do the protein constituents vary in accessions of African nightshades that differ in germination percentages? Based on existing literature we hypothesised that the time of berry harvesting affects the germination of African nightshades, where the berries harvested at the later maturity stages have a higher germination percentage and accessions of African nightshade differ in their protein constitution irrespective of their maturation stage.

## Materials and methods

### Seed source

African nightshade seeds were collected from five counties in western Kenya. The selected counties were known to have high activities in the production, marketing, and consumption of African nightshades. The seeds were collected from farmers, local seed traders, and agro-veterinary shops that sell seeds from registered seed companies (e.g., Simlaw seed company). Accessions Abuku 1 and Abuku 2 were obtained from the JKUAT African Indigenous Vegetable (AIV) project in Kiambu county. Accessions Olevolosi and SS 40 were obtained from the World Vegetable Center (WVC) in Arusha, Tanzania. Accession 18 was collected from Simlaw seed company in Nakuru county while accessions 1 and 7 were collected from a farmer in Kakamega county, accession 33 from a farmer in Kisii and Acc3 from a farmer in Siaya county. The nine accessions were selected to represent the various seed sources available to the farmers in areas that are known for production of African nightshades (Table 1).

**Table 1:**
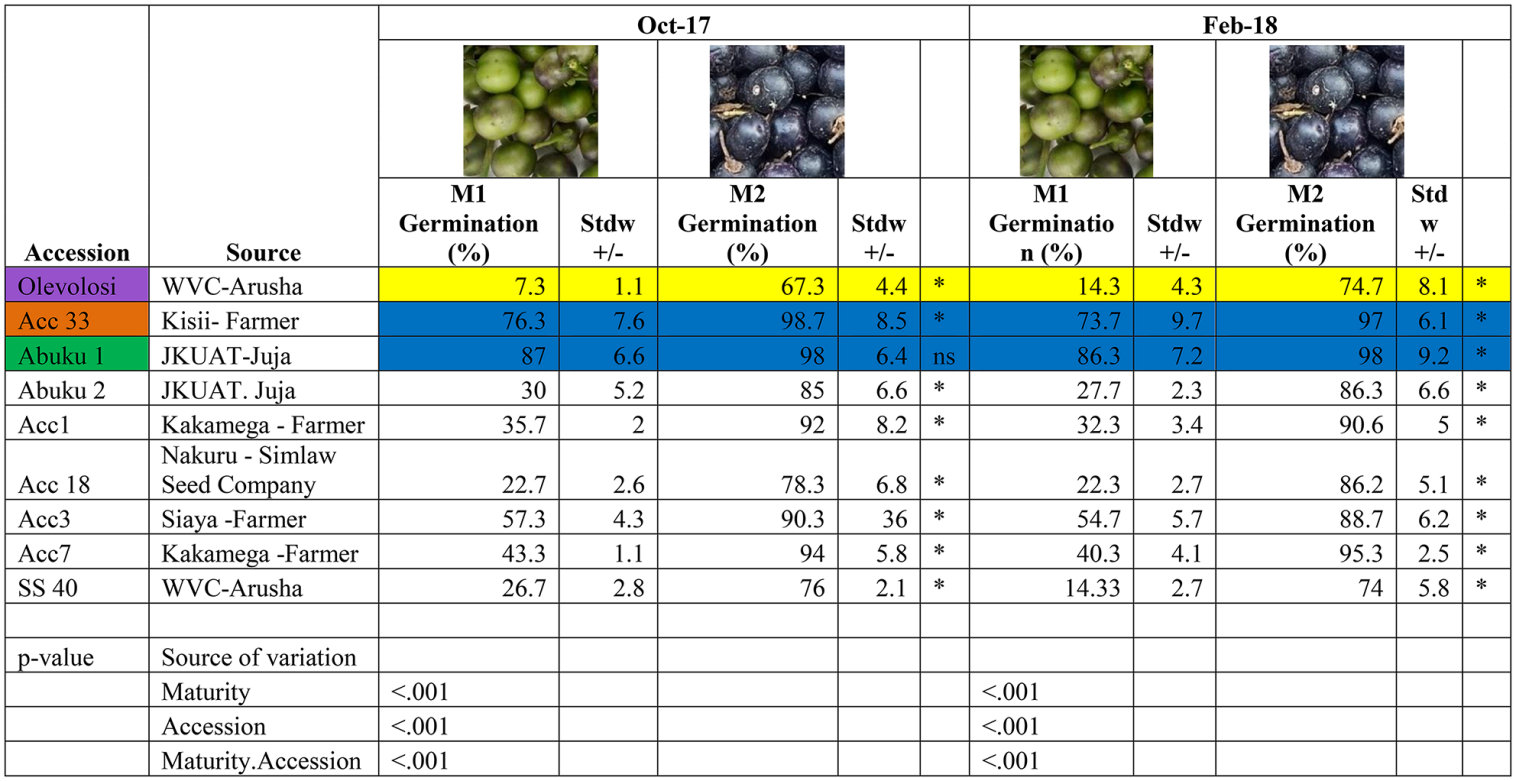
Germination percentage (mean +/- stdw) of nine African nightshade (*Solanum scabrum*) seed accessions harvested at two maturity stages (M1 and M2).

M1 = the mature green stage; M2 = the ripe purple stage. A (*) in the last column of each year indicates significant difference in the means, while (ns) indicates no significant difference between the means (least significant difference was used to separate means and Bonferroni was used to adjust p value *p* < 0.05), *n* = 3. The photos in the table are exemplary berries of Acc 33 to display colour at the specific maturity stage. Purple background = Olevolosi; orange background = Acc 33 and green background = Abuku 1. Yellow background = used within the manuscript as low germinating accession; blue = used within the manuscript as well germinating accessions.

### Field experiment for seed production

The field experiment was conducted at the Jomo Kenyatta University of Agriculture and Technology farm block A (-1.09002690011, 37.010965343). The precipitation of the region is bimodal with the long rainy season starting in March until July, whereas the short rainy season lasts from September to November. The region shows an average rainfall of 1,129.8 mm, an average temperature of 20.3 °C (68.5 °F), and an altitude of 1490 m above sea level. The soils are predominantly heterogeneous clay loamy soils which are inherently fertile.

The experiment was laid out in a Randomised Complete Block Design (RCBD) replicated 3 times as block A, B, and C. Each block had nine plots of 4 m x 2.4 m with 140 plants in each block. Berries were harvested from ten plants which were randomly selected per plot at two maturity stages: M1 = Mature green (at the onset of colour change from green to purple, Table 1); M2 = fully ripe stage (when the fruits were fully ripe and had a deep purple colour, Table 1). The berries at M1 stage were harvested from the first trusses 68 days after planting while the berries at M2 stage were harvested from the same trusses 80 days after sowing. The berries from the first trusses which flowered, set, developed and matured at the same time were selected, marked and harvested. Seeds from each of the samples were extracted from the berries immediately after harvesting, dried at 25 –30 °C to a constant water content of 8.7%, and stored at 20–30% relative humidity in an air tight glass jar at room temperature for three months. A germination test was not conducted at harvest, the first germination test was done three months after harvest in 2017 and 7 months for the second experiment in 2018.

### Germination assay

 Three replicates of 100 seeds each from M1 and M2 of nine African nightshade accessions were used in the germination assay (Table 1, for pictures of plants and berries see Figure S1). 100 seeds each were placed in a plastic 9 cm Petri dish lined with Whatman cotton filter paper, and moistened with distilled water. The Petri dishes were then placed in a growth chamber at a constant temperature of 25 +/-1 °C in darkness^16^. The experiment was laid out in a Completely Randomized Design (CRD). Observations were recorded on the germination percentage for each treatment. Germination was determined based on radicle emergence daily, starting 3 days after the onset of the experiment until day 14.

### Seed preparation for protein extraction

Out of the nine *S. scabrum* accessions, three were selected for proteomic analysis based on germination percentage results. Two accessions were chosen with the minimum differences between the two maturity stages (Abuku 1 and Acc 33) and one accession that showed the highest difference in germination percentage between the two stages, and the overall lowest germination percentage (Olevolosi). Around 100 mg seeds from two maturity stages and three accessions were instantly frozen in liquid nitrogen (LN) in January 2018. Samples were then ground with a ball mill (MM 400, Retsch, VERDER Group, Netherlands) through stainless steel beads (7 mm diameter) in a reaction tube and either stored at -80 °C or immediately used for protein extraction.

### Phenol protein extraction

The protein extraction was performed after Faurobert et al. (2007)^17^. First, 750 µl of ice cold extraction buffer (700 mM sucrose, 500 mM Tris, 50 mM EDTA, 100 mM KCl, 2 ml 2% ß-mercaptoethanol, 1 ml 2% PMSF, in 100 ml ddH_2_O, pH adjusted to 8.0 with HCl) was added to homogenised seed powder. The solution was vortexed and afterwards incubated for 10 min on ice. Then, 750 µl of phenol was added to the samples. The samples were shaken at RT for 30 min. Afterwards samples were centrifuged for 10 min at 12,000 g at 4 °C. The upper phase was then transferred to a new reaction tube (~ 400 µl). The same volume of ice cold extraction buffer was added to the upper phase and vortexed. Samples were centrifuged for 10 min at 12,000 g and 4 °C. The upper phase was transferred to a new reaction tube. The sample was then filled up with precipitation solution (0.1 M ammonium acetate in methanol) and incubated overnight at -20 °C. On the morning of the next day, samples were centrifuged for 3 min at 15,000 g at 4 °C. Pellets were resuspended three times in 1 ml precipitation solution and centrifuged for 3 min at 15,000 g at 4 °C after each resuspention. Afterwards, samples were resuspended in 1 ml acetone solution (80% (v/v) acetone). Samples were then centrifuged for 3 min at 15,000 g at 4 °C. The supernatant solution was discarded and pellets were dried at RT under a fume hood. Finally, protein pellets were weighed and frozen at -80 °C until further use.

### 2D IEF/SDS-PAGE

About 4 mg of protein pellet suspended in 350 µl rehydration buffer was used for a 2D gel electrophoresis. The samples were transferred to IEF strips (18 cm, pH 3–11 NL, GE Healthcare, Freiburg, Germany). Isoelectric focusing was performed according to Mihr et al. (2003)^18^. A polyacrylamide gel (13.5 ml 49.5T/3 C acrylamide, 15 ml tricine gel buffer (3 M Tris, 0.3% (w/v) SDS, 6 ml 87% glycerine, 10.5 ml bidest H_2_O, 150 µl 10% APS and 15 µl TEMED) was poured between two glass plates (20 × 20 cm in size with 1 mm thickness of the gel). The IEF strips were equilibrated for 15 min in 40 ml equilibration solution (50 mM Tris-Cl (pH 8.8), 6 M Urea, 30% (v/v) glycerine, 2% (w/v) SDS, a small tip of bromophenol blue) containing 0.4 g dithiothreitol (DTT). A subsequent bath in 40 ml equilibration solution containing 1 g iodoacetamide (IAA) without DTT for 15 min ensued, which was followed by a washing step in tricine gel buffer. The IEF strips were then placed on top of the acrylamide gel and run for 18 h at max. 500 V and 30 mA per gel.

### Gel staining procedure

Proteins were fixed in the gel for 2 h (15% ethanol, 10% acetic acid) and stained over night with Coomassie blue CBB G-250 (Merck, Darmstadt, Germany) in a solution containing 1% (w/v) ortho-phosphoric acid (85%), 10% (w/v) ammoniumsulfate, and 20% ethanol (v/v).

### Processing of gel images of different maturity stages of the berries

Scanned images of Coomassie colloidial stained gels were analysed according to Berth et al. (2007)^19^, using the Delta2D software 4.4 (Decodon, Greifswald, Germany). Three replicate gels per accession and maturity stage (M1 and M2) were analysed and spots were automatically detected. Minor corrections of gel disturbances were manually done. For determining significant differences in spot patterns between M1 and M2 stage berries within an accession, a Student´s t-test based on the normalised relative spot volume was performed (p-value ≤ 0.05). Additionally, only spots with a fold change higher than 1.5 were taken into consideration. Three individual comparisons were made between the different groups (M2 versus M1 Olevolosi; M2 versus M1 Abuku 1; and M2 versus M1 Acc33). PCA analysis can be found in Figure S2.

### Mass spectrometry analyses

Based on spot ID and differences in spot volume, those spots were selected for picking, which had a high fold change in a single comparison or which showed up in more than one comparison. Excised protein spots were in-gel-digested with trypsin as described before^20^ and analysed by high pressure liquid chromatography (HPLC) electrospray (ESI) quadrupole (Q) time-of-flight (ToF) mass spectrometry (MS) using an Easy nano LC (Thermo scientific) coupled to a micrOTOF Q II (Bruker Daltonics) using parameters given in Klodmann et al. (2011)^21^.

Data processing was carried out with the ProteinScape 2.1 software (Bruker Daltonics). For protein identification, *Solanum tuberosum* protein sequences of “the working gene model set v6.1” (DM_1–3_516_R44_potato.v6.1.working_models.pep.fa.gz) were downloaded from Spud-DB (http://spuddb.uga.edu/) on February 16th, 2021. Database search was carried out applying standard parameters, as given in Klodmann et al. (2011)^21^. Complete list of proteins can be found in Table S6.

### Reference map (GelMap)

**Fig. 1:**
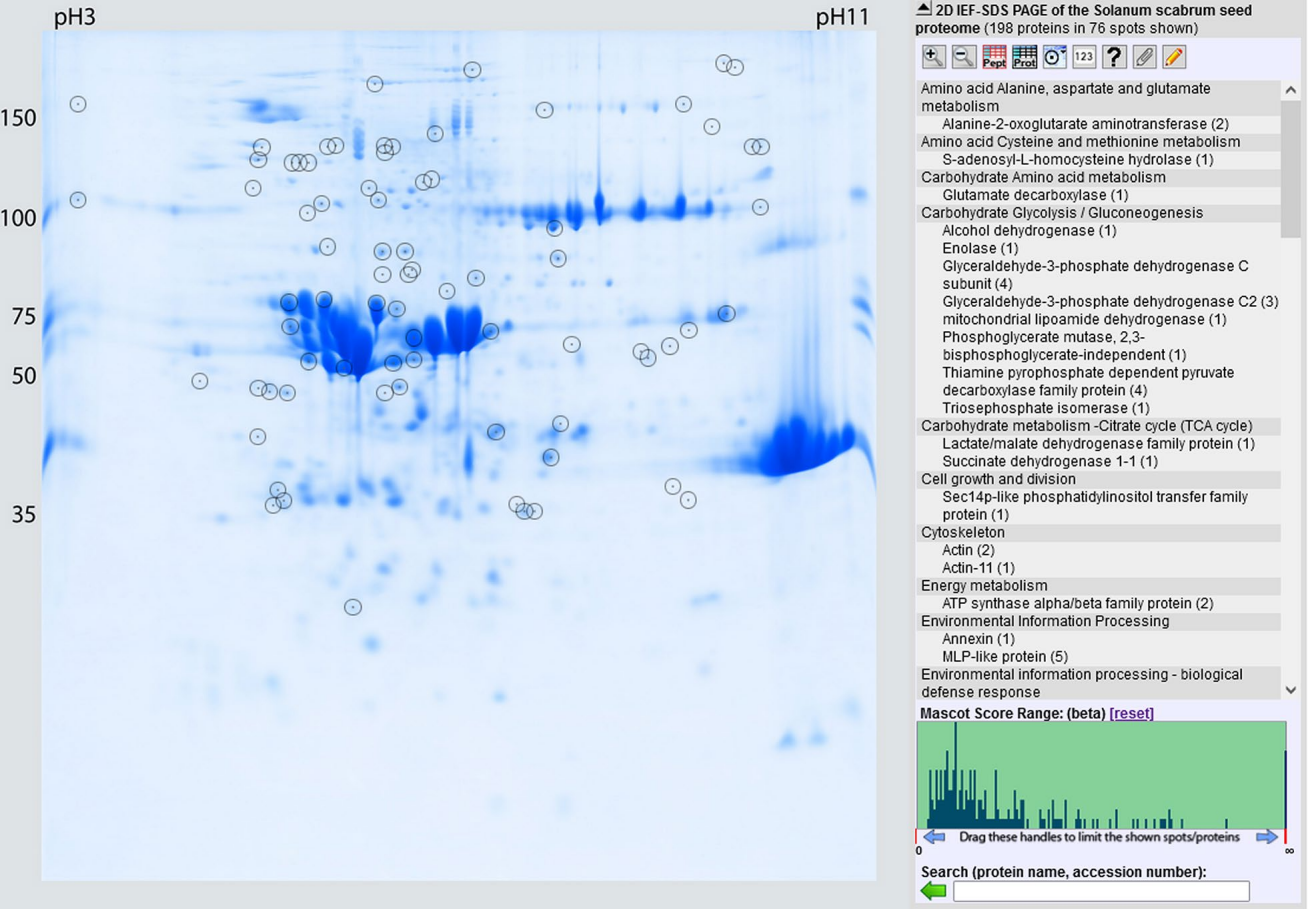
GelMap of the proteomic dataset of this study. Screenshot from the website. The GelMap is accessible via www.gelmap.de/2700 (password: Solanum2024). Protein data from all gels were merged and interactively linked to a IEF-SDS gel of Olevolosi. The map includes all information (functional context of proteins, spot regulations, as well as physiological as well as mass spectrometry-based parameters of proteins).

To better visualise our protein data, an interactive reference map was created using GelMap (www.gelmap.de)^22^ (Fig. 1). Therefore, the IEF-SDS gel of Olevolosi (M2) was used as a basis to indicate all identified proteins of all gels. The reference map is accessible via www.gelmap.de/2700 (password: Solanum2024).

### Statistical analyses

Germination data was analysed for statistical significance by a two way Analysis of Variance (ANOVA). The data on germination percentage was arcsine transformed prior to analysis (data included in Table 1 represent original values). Fischer’s least significant difference (*p* < 0.05) was used to determine significant differences between accessions. The data was analysed using GenStat version 22.1 Edition package.

Venn diagrams were prepared based on spot ID with the help of Software Venny Version 2.1.0 by Juan Carlos Oliveros (https://bioinfogp.cnb.csic.es/tools/venny/).

Heat maps were drawn with R (R version 4.3.3, R Core Team, 2024) in R Studio (version 2023.12.1.402, Posit Team, 2024) with library ´gplots´^23^.

## Results

### Germination assays

The data on seed germination for nine different seed accessions of *S. scabrum* harvested at two development stages is presented in Table 1. There was a significant effect (*p* < 0.05) of the maturity stage, the accession and their interaction (Table 1). As evident, seeds harvested at the ripe stage (M2) recorded higher germination percentages compared to seeds harvested at the mature green stage (M1), for both experiments conducted in 2017 and 2018. Accession 1 exhibited the highest range in germination percentage (15%) with a maximum of 46% and a minimum of 31% in 2017, and 14% (maximum of 39% and a minimum of 25%) in 2018 for the M1 seeds. Olevolosi recorded the lowest range in germination percentage of 2% in both 2017 and 2018. For the seeds harvested at the ripe stage, Accession 18 exhibited the highest range of 10% with a maximum of 86% and a minimum of 76% in 2017 while Abuku 2 recorded the highest range 7% in germination percentage in 2018. Abuku I had the lowest range of 2% in germination percentage in both years.

The Mean Germination Time (MGT) of the M1 seeds was higher than that of seeds harvested at the M2 stage. At the M1 stage Accessions 33 and Abuku 1 showed the least average time needed (5.38 and 5.22 days) for germination in 2017 and 2018, respectively (Table S7). For the seeds harvested at the M2 stage, Accession 33 recorded the least average time needed for germination (4.78, 4.46 days in 2017 and 2018, respectively). Olevolosi recorded the longest average time for germination for both M1 and M2 seeds, in 2017 and 2018 (Table S7).

The Mean Germination Rate (MGR) of the seed accessions harvested at the M2 stage was greater than that of the seeds harvested at the M1 stage. Abuku 1 recorded a greater proportion of seeds germinating within the given period of time while Olevolosi recorded the least proportion of germinated seeds within the period of time, for both M1 and M2 in 2017 and 2018.

On basis of the statistical analysis of the germination assays, three accessions were chosen for further investigation through proteomic analysis. The three include Olevolosi as the accession with the lowest germination percentage and Abuku 1 and Acc 33, as they both show high germination percentages for the M2 stage, however vary at the M1 stage, where Acc 33 displayed lower germination. While there was no significant difference in germination percentage (*p* < 0.05) between Abuku 1 and Acc 33 in 2017, Olevolosi showed significantly lower germination percentages than the other accessions (Abuku 1 and Acc 33) in both experiments for seeds harvested at the M2 stage.

### Proteomic analyses of selected spots

Proteomic analyses were performed to determine proteins changing in abundance as an effect of maturity stage at harvest of *S. scabrum* seeds. Gel analysis displayed a total of 563 spots, 108 differentially abundant spots (DAS) for Olevolosi (higher abundant in M1 = 43; in M2 = 65), 126 DAS for Acc 33 (higher abundant in M1 = 70; in M2 = 56), and 130 DAS for Abuku 1 (higher abundant in M1 = 60; in M2 = 70) (Table 2).

**Table 2:**
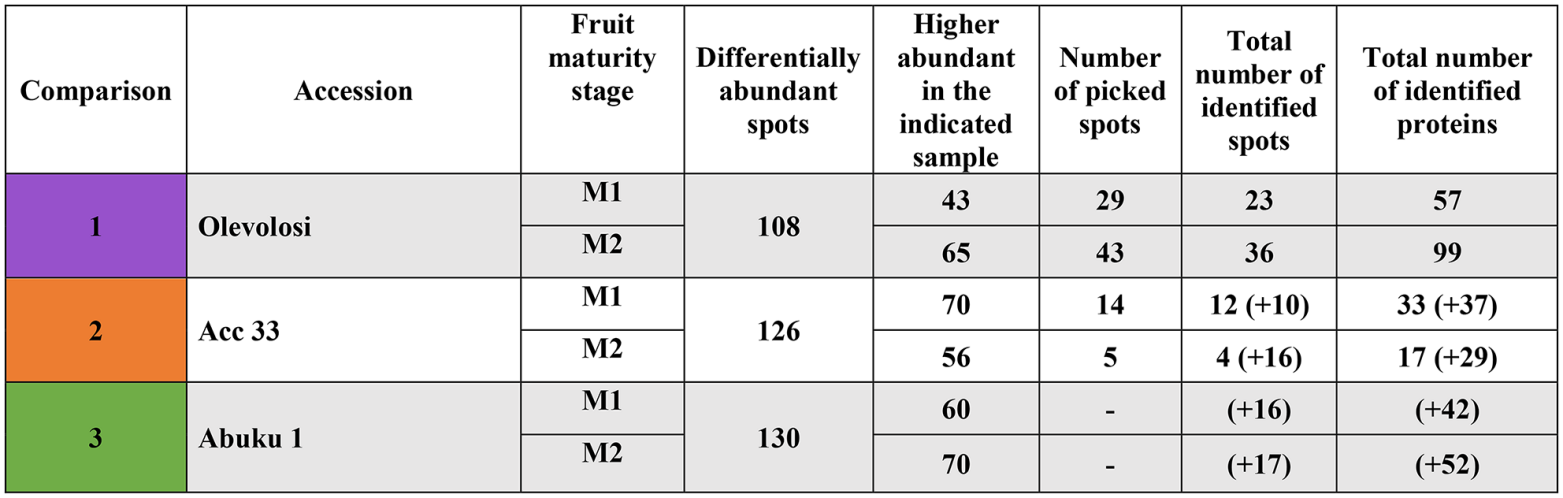
Overview of total proteins found on gels, the total differentially abundant proteins in the comparisons and the identified number of proteins.

Numbers in brackets = identified from other gels (e.g. 12 (+ 10) = 12 spots identified from Acc 33 gel + 10 spots identified from spots picked from other gels). Spots = individual spots with spot ID in gel. Proteins = individual proteins in all spots. For Abuku 1, no spots were picked from Abuku 1 gels but were identified through picking of spots within other gels with same spot ID.

**Fig. 2:**
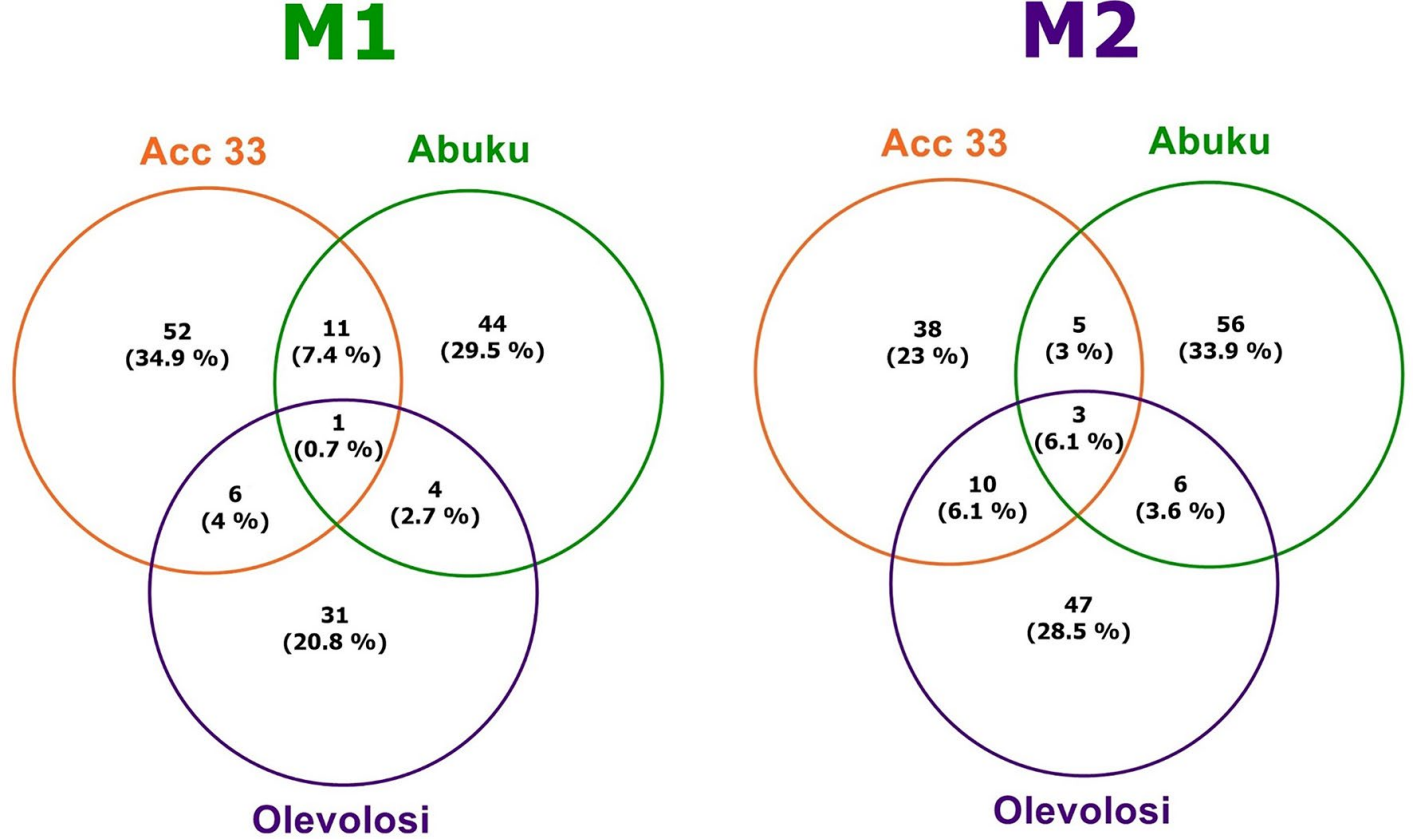
Venn diagrams based on differentially abundant spots for M1 or M2 for the accessions Acc 33, Abuku 1 and Olevolosi. Data are based on Spot ID before the identification of individual proteins and were done separately, for spots higher abundant at M1 (left) and M2 (rigth) stage. Figure was drawn by hand based on data analysis with online software Venny Version 2.1.0 by Juan Carlos Oliveros (https://bioinfogp.cnb.csic.es/tools/venny/).

Based on the spot ID number, a separate comparison of the three accessions for M1 and M2 was performed (Fig. 2). The majority of DAS was found to be specific for each accession (up to 34.9% of total DAS in Acc 33 M1). However, minor fractions of the identified spots were also found in overlaps of the accessions. Acc 33 and Abuku 1, which represent the more readily germinating accessions, displayed an overlap of 11 spots (7.4% of DAS) for M1 and 5 spots (3.0% of DAS) for M2. Comparison between Olevolosi, which represents the lowest germinating accession, and Acc 33 revealed 6 overlapping spots (4% of DAS) and Olevolosi compared to Abuku 1 showed 4 spots (2.7% of DAS) for M1 and 6 spots (3.6% of DAS) for M2. All three accessions displayed an overlap of only 1 spot (0.7% of DAS) for M1 and 3 spots (6.1% of DAS) for M2.

Spot selection for picking from the gels was based on high regulation and Venn comparison. Therefore, only those spots were extracted from gels with high regulation, and spots which were present in two accessions with the same regulation as well as spots which based on their ID were overlapping between accessions. Thus, for MS analysis, a total of 91 spots was picked. The total number of identified spots was 75, representing a 81.4% identification rate. In total, 206 proteins were identified in these 75 spots (Table 2). With the publicly available tool GelMap, a protein reference map was generated, which may be complemented and used in future research projects dealing with seed proteins of African nightshades (Fig. 1).

**Fig. 3:**
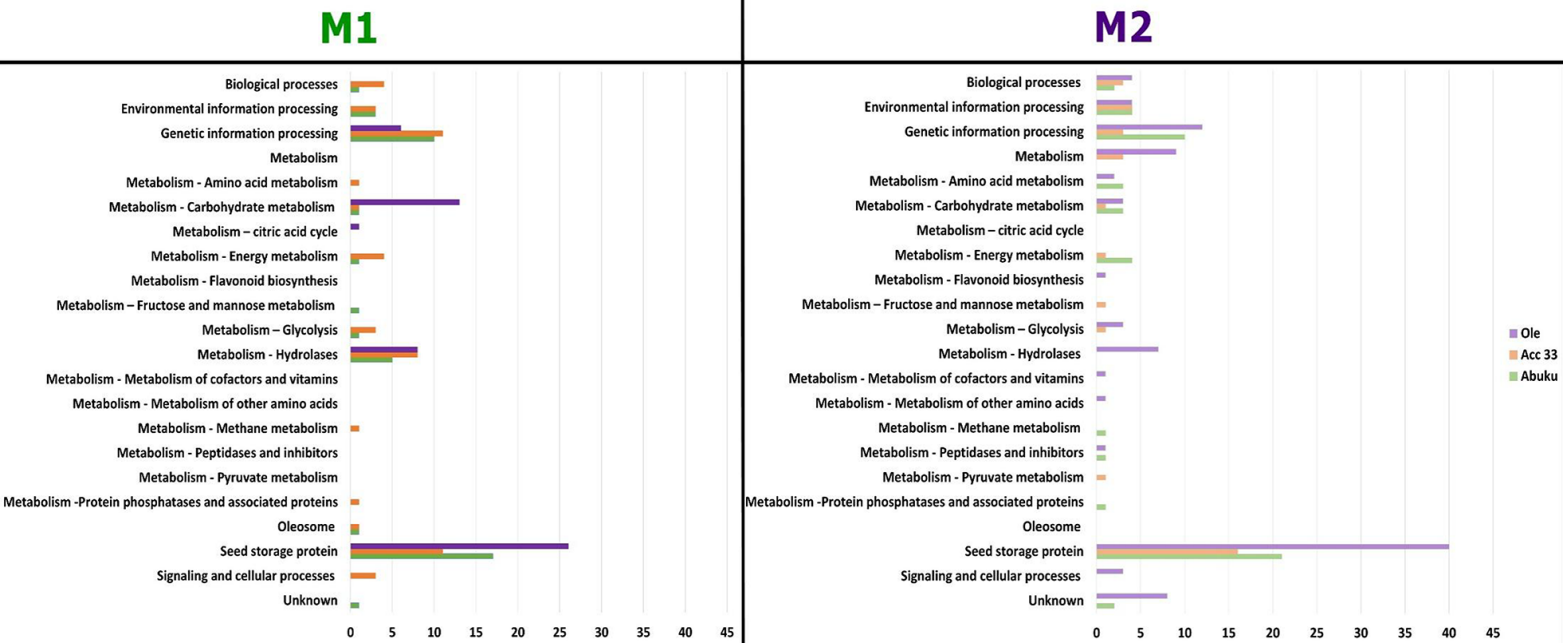
Functional analysis of all identified proteins for accessions Olevolosi, Acc 33 and Abuku 1 at stages M1 and M2. Numbers are based upon all identified proteins. Categorisation was based on KEGG Pathway Database classification^58^. M1 = green mature stage; M2 = purple ripe stage. Ole = Olevolosi (purple bars = M1, light purple bars = M2), Acc 33 = Accession 33 (orange bars = M1, light orange bars = M2), Abuku = Abuku 1 (green bars = M1, light green bars = M2).

The identified proteins were sorted according to their function based on KEGG functions. Proteins associated with the metabolism of co-factors and vitamins, metabolism of pyruvate, and the metabolism of flavonoid biosynthesis were found in seeds at the M2 stage, but not at the M1 stage, while other proteins associated with the metabolic citric acid cycle and oleosomes were present in M1 and were missing in the M2 stage in all three accessions (Fig. 3). Overall, most proteins were assigned to be seed storage proteins with increased numbers at M2 and higher numbers for Oleovosi, followed by the functional classes “metabolism – hydrolases”, “genetic information processing” and “metabolism – carbohydrate metabolism”. In total, 22 different functions were assigned (Fig. 3). Proteins categorised in seed storage were further analysed.

### Identification of proteins from DAS displayed a high number of seed storage proteins in the low germinating accession Olevolosi

**Fig. 4:**
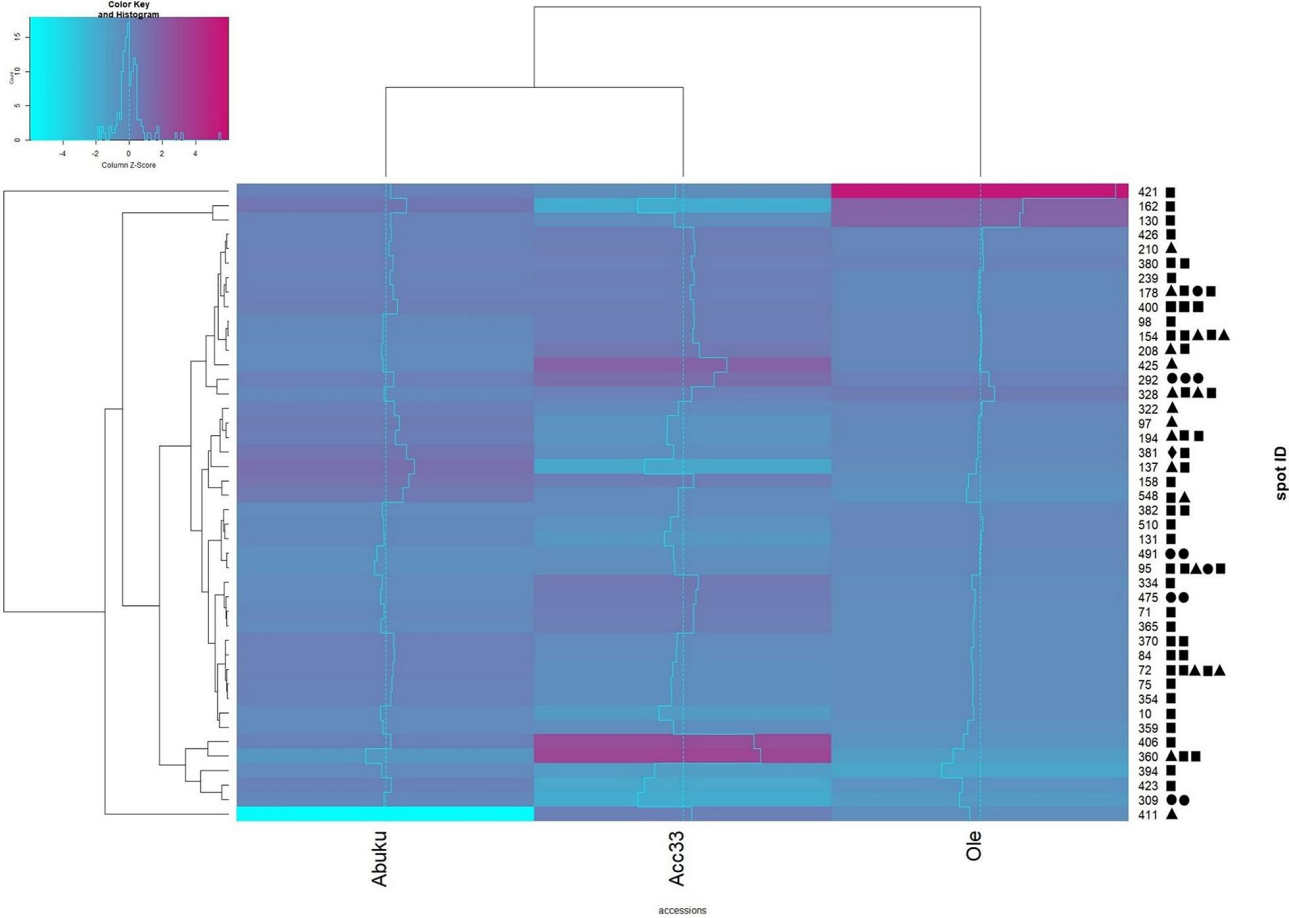
Heat map based upon regulation of normalised spot volume (M2/M1) for seed storage proteins. Pink = positive regulation (= M2 > M1), cyan = negative regulation (= M1 > M2). Light blue lines indicate how strong the reaction is compared to the other spots regulation of the other accessions.  = RmlC-like cupins superfamily protein;  = Cruciferin;  = Cupin family protein;  = Vicilin. Multiple symbols of the same type, display different proteins in one spot. Names, regulation and further information can be taken from supplementary tables S3 and S4.

Data processing displayed 4 different types of seed storage proteins in the picked spots. These included RmlC-like cupins superfamily protein, cruciferin, cupin family protein and vicilin (Table 3; Fig. 4). The highest abundance within the DAS was found in Olevolosi in form of RmlC-like cupins superfamily protein (15 spots M1, 16 spots M2). This was also the dominant seed storage protein for Acc 33 (7 spots M1, 4 spots M2) and for Abuku 1 (5 spots for M1, 6 spots for M2), but with a lower number of DAS. Cruciferin was the second protein group in terms of numbers within DAS of Olevolosi (4 spots in M1, 6 spots in M2), Acc 33 (2 spots in M1, 4 spots in M2) and Abuku 1 (3 spots in M1, 5 spots in M2). The cupin family protein was detected in Olevolosi (1 spots in M1, 4 spots in M2), Acc 33 (0 spots in M1, 2 spots in M2) and Abuku 1 (3 spots in M1, 1 spots in M2). Vicilin was only found to be present in Acc 33 M1 and in Abuku 1 M2 (Table 3). However, numbers are based on selected DAS. If the analysis of the whole proteome would display a different picture is still unclear.

**Table 3:**
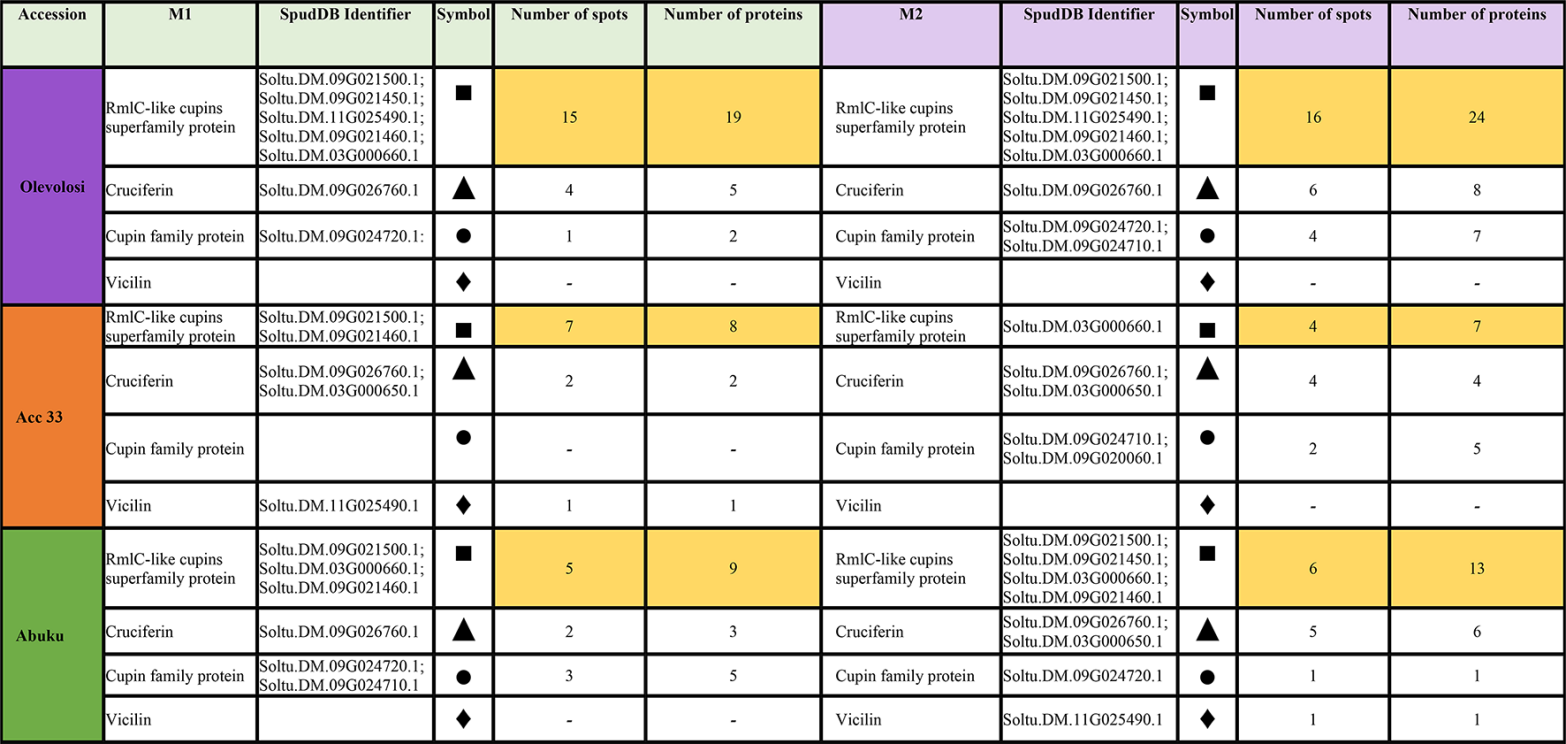
Seed storage proteins identified in the three accessions at M1 and M2 stage based on spot and protein number.

Marked in yellow: highest number of identified specific seed storage proteins among the picked spots within an accession.  = RmlC-like cupins superfamily protein;  = Cruciferin;  = Cupin family protein;  = Vicilin.

### Regulation analysis of identified seed storage proteins

Spot IDs for the calculation of the heat map in Fig. 4 were based upon identification of seed storage proteins within the list of identified spots for each gel. Only those spots, which were displaying at least one potential seed storage protein, were used and their regulation was compared in the heat map. The heat map is based upon abundance of normalised spot volumes M2/M1 for seed storage proteins that displayed changes as seeds transitioned from M1 to M2 stage. For example, spot 421 was significantly higher abundant in M2 stage seeds than in M1 stage seeds of accession Olevolosi (Fig. 4). Some spots, like ID 400, showed a transition of accumulating storage proteins as seeds changed from mature green stage to the ripe stage (Tables S3 and S4). The heatmap displayed a hierachical clustering with a clear separation of the well germinating and the the low germinating accessions, when only the abundance of the spots containing seed storage proteins were analysed. Therefore, differences in seed storage protein composition between the accessions at the two maturity stages (M1 and M2) might display one reason, why accessions differed in germination rates.

### Heat map of interesting spots beyond the seed storage proteins displayed clustering of the well germinating accessions

**Fig. 5:**
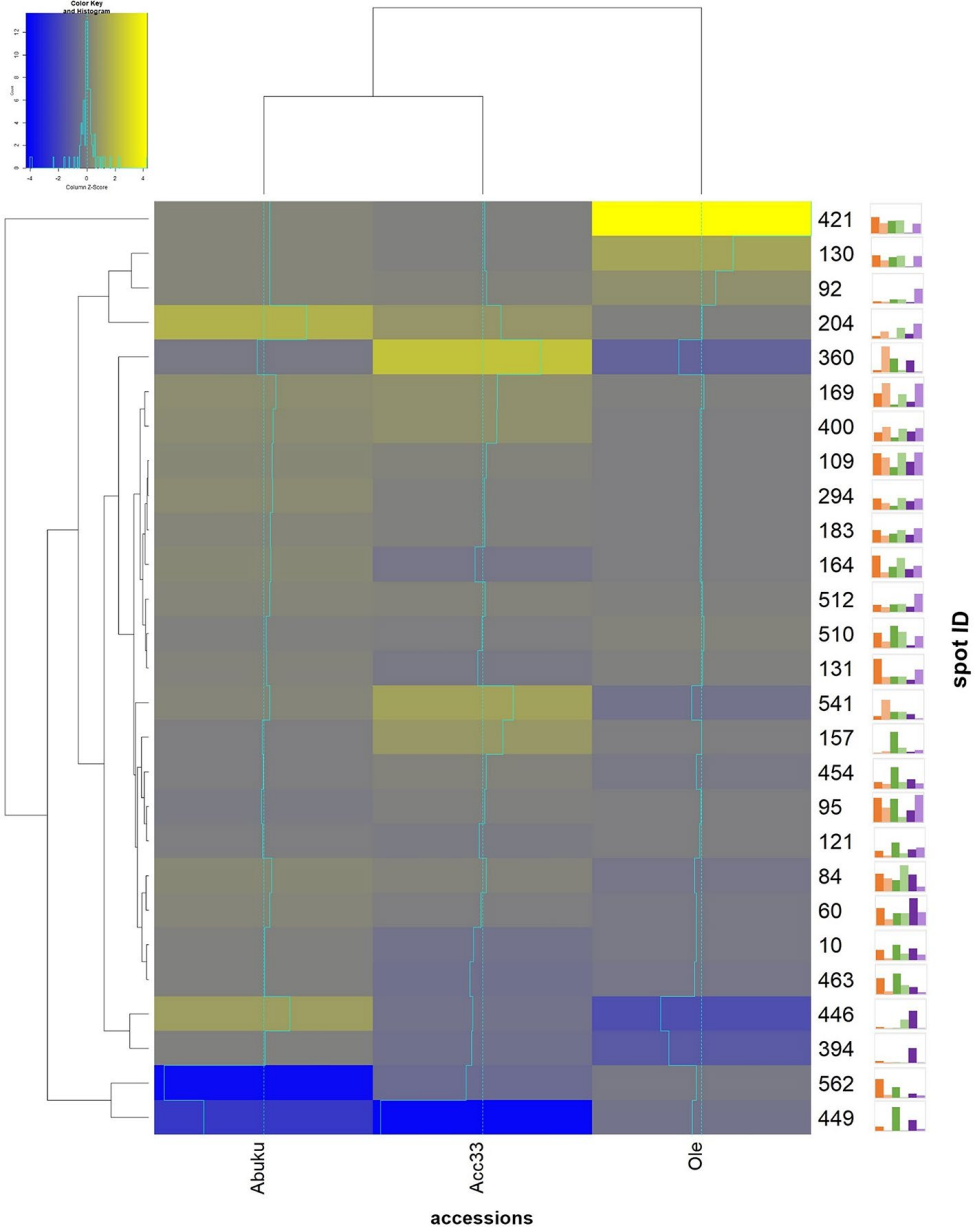
Heat map based upon regulation of normalised spot volume (M2/M1) for interesting spots excluding the seed storage proteins. Yellow = high regulation (= M2 > M1), blue = negative regulation (= M1 > M2). Light blue lines indicate how strong the reaction is compared to the other spots regulation of the other accessions. Graph behind the spot ID number = Mean relative spot volume obtained according to three gels of M1 seeds or M2 seeds illustrated by graphs. The first bar (orange) represents the mean normalised spot volume in the gels of M1 seeds of Accession 33. The second bar (light orange) represents the mean normalised spot volume in the gels of the M2 seeds from Accession 33. The third bar (green) stands for the mean normalised spot volume in the gels of the M1 seeds of Abuku 1. The fourth bar (light green) represents the mean normalised spot volume in the gels of the M2 seeds of Abuku 1. The fifth bar (purple) represents the mean normalised spot volume in the gels of the M1 seeds from Olevolosi. The sixth bar (light purple) stands for the mean normalised spot volume in the gels of the M2 seeds of Olevolosi. Names, regulation and further information can be taken from supplementary Table S5.

After extracting the seed storage protein entries from the data, the remaining data was analysed to identify the proteins differing in regulation between the well and low germinating accessions. For this purpose, proteins were identified, which either displayed an overlap according to the Venn diagrams (Fig. 2), were found to be higher abundant in the well germinating accessions in M1 and were then only identified in the M2 seeds of the low germinating accessions (and vice versa), and also those proteins, which displayed a high abundance in the high or low germinating accessions in only one of the maturity stages. The selected spots were then displayed in a heat map for their regulation based upon the M2/M1 ratio of spot abundance (Fig. 5).

Again, the abundance of the selected spot IDs allowed a clustering of the accessions in well and low germinating accession. Proteins which display a known seed maturation function were also identified e.g. LEA proteins (late embryogenesis abundant protein (LEA) family protein (higher abundance in Acc33 M2: spot ID 175; Ole M2: spot ID 166, 169, 175), late embryogenesis abundant domain-containing protein / LEA domain-containing protein (higher abundance in Acc33 M2: ID 79 and 330; Ole M2: spot ID 79, 91, 92, 330, 333) and Major latex -like proteins (MLPs; higher abundance in Acc33 M1: spot ID 553, Acc33 M2: Spot ID 203, 204; Abuku 1 M1: spot ID 553; Abuku M2: spot ID 203, 204; Ole M2: spot ID 203, 204). However, as these were present in all accessions based on DAS analysed, differences in germination might not be explained by these.

Further proteins which were identified (excluding the seed storage proteins) comprised e.g. phosphoglycerate kinase (higher abundance in Acc33 M1: 130, 131, 421; Ole M2: 130, 131, 421), glutamate decarboxylase (higher abundance in Abuku 1 M1: spot ID 95; Ole M2: spot ID 95), eukaryotic translation initiation factor 4A1 (higher abundance in Acc33 M1: spot ID 121; Abuku 1 M1: spot ID 121) and an oleosin family protein (higher abundance in Acc33 M1: spot ID 562, Abuku 1 M1: spot ID 562).

## Discussion

### Colour change indicates ripening in *S. scabrum* accessions

The stage of maturity had a significant effect (*p* < 0.05), on seed germination for all accessions of African nightshade analysed in this study (Table 1). These findings are in accordance with Tetteh et al. (2018)^7^ who reported that tomato seeds harvested at later stages of maturity germinated better than those harvested at earlier stages of maturity. The significantly higher germination percentages recorded in seeds harvested at the purple maturity stage (M2) may be due to the completion of the development of seed organs and maximum dry matter accumulation in the seeds as compared to the seeds harvested earlier at the mature green stage (M1). These results conform with Valdes et al. (1998)^11^, who reported this also for tomato fruits. However, the nightshade seed germinability did not change during the storage.

The Abuku 1 and Acc33 seeds displayed a high germination rate at the two maturity stages, the germination percentage observed for the two well-germinating accessions was above 85%, which is the recommended percentage for high-quality seeds^24^. However, Olevolosi displayed significantly lower germination percentages at the two maturity stages and did not reach the value for high-quality seeds (in assay 1: 7.3% against 76.3% and 87.0% for Acc 33 and Abuku 1 at M1 and 67.3% against 98.7% and 98.0% for Acc 33 and Abuku 1, respectively at M2 stage, Table 1). The well germinating accessions, Abuku 1 and Acc 33 recorded a higher germination percentage compared to Olevolosi.

Berries of *S. scabrum* display a colour change from green to purple as berries advance in age (see Table 1, pictures for maturity stages). The protein data based on LEA proteins, MPLs and proteins found during seed maturation and drying stages also suggests, that seeds are more mature and start to ripen at the M2 stage (Fig. 5, Table S5, see further discussion). Similarly in tomatoes, it was shown that high-quality tomato seeds could be obtained from half-ripe and fully-ripe berry stages of different accessions based on the colour change of the berries (from green to red), leading to a high germination percentage and seedling emergence^7^. In contrast, a study by Ahmed et al. (2018)^25^ confirmed that paprika berries harvested at the red ripe stage could give rise to superior quality seeds unlike those harvested at dark green and colour breaker stage. The superior seeds of fruits harvested at the red stage were attributed a physiological maturity of seeds which might be related to increased accumulation and assimilation of reserves from source to sink. Therefore, colour change might be a good visual criterion for farmers to detect seed maturity in *S. scabrum*. Nevertheless, the low germination rate of Olevolosi M1 might also be explainable through this, as other accessions might already be further along in their ripening process and therefore are displaying a higher germination percentage at M1 stage.

### Identification of proteins displayed a high number of seed storage proteins within the DAS in the low germinating accession Olevolosi

Seed storage proteins were the predominant protein group within the DAS found in the analysed African nightshade seeds (Figs. 1 and 3). Hay et al. (2017)^24^ reported that proteins accumulate significantly in the developing seed, whose main function was to act as a storage reserve for nitrogen, carbon, and sulphur and these proteins are rapidly mobilised during seed germination. Seed storage proteins were previously classified based on their solubility^26^ and are traditionally sorted into families and superfamilies^27^. However, recently these proteins have been categorised into superfamilies based on sequence information and amino acid conservation. Four different types of seed storage proteins were identified in the analysed *S. scabrum* seeds. These included RmlC-like cupins superfamily protein, cruciferin, cupin family protein, and vicilin (Table 3; Fig. 4). In accordance with the findings of Koshiyama et al. (1983)^28^, RmlC-like cupins superfamily protein was the most abundant in African nightshade seeds. These proteins belong to the 11–12 S globulins and are the most abundant seed storage proteins among higher plants that are synthesised during seed maturation on the mother plant^23^.

Despite RmlC-like cupins superfamily proteins being the most abundant storage proteins within the DAS in the African nightshade seeds, their role in seed maturity and development is not as well documented as their roles in other aspects of plant development and metabolism (Table 3; Fig. 1). However, the specific role of RmlC-like cupins in seed germination can vary depending on the plant species and the particular cupin protein in question. The functionally diverse superfamily can be divided in enzymatically active and non-active proteins^29^. Also within the enzymatic active group of proteins, the functions are diverse, ranging from metal binding or sugar binding proteins to sugar isomerases (epimerases), oxalate oxidases (OXOs), superoxide dismutases (SODs) and many other functions^30^. However, further investigations would have to take place to characterise the functions of the specific proteins found in this study.

Cruciferin was the second most abundant seed storage protein within Olevolosi (4 spots in M1, 6 spots in M2), Acc 33 (2 spots in M1, 4 spots in M2), and Abuku 1 (3 spots in M1, 5 spots in M2) (Table 3). Cruciferins are also one group of 11 S globulins, which are known to accumulate during the seed filling phase, e.g. in rape seeds^24,31^ and serve as a source of nitrogen and amino acids for the germinating embryo^32^. However, in mature dry seeds of *Brassica napus*, levels of cruciferin were hardly detectable^31^ and this was attributed to higher turnover of cruciferin in later seed development^33^. This might indicate, that even in M2 stage, seeds of *S. scabrum* might still be metabolically active and not fully mature yet. However, this would have to be tested further.

Other cupin family proteins were also detected in Olevolosi (1 spots in M1, 4 spots in M2), Acc 33 (0 spots in M1, 2 spots in M2), and Abuku 1 (3 spots in M1, 1 spots in M2), showing that these proteins were the most abundant seed storage proteins within the DAS analysed in *S. scabrum* seeds. Vicilin, however, was only found to be present in Acc 33 M1 and in Abuku 1 M2 seeds (Table 3). Vicilin proteins are 7 S globulins^34^ and have diverse functions including but not limited to seed desiccation tolerance^35^ and plant defences against fungi and microbes^36,37^. In a proteomic analysis of germinating tomato seeds, these were found to be highly abundant in the embryo as well as the endosperm^38^. It might therefore be interesting to analyse the changes in seed storage proteins during germination of *S. scabrum* seeds for a comparison to other Solanum species.

### General maturation patterns indicated by LEA proteins and MLPs

Maturation is an essential step in seed development that is characterised by a decline in reserve synthesis, acquisition of desiccation tolerance, and dormancy. The heat map based upon abundance of the normalised spot volume M2/M1 for the interesting spots (excluding seed storage proteins) showed changes as seeds transitioned from M1 to M2 stage (Fig. 5, see also Fig. 1 and GelMap online (www.gelmap.de/2700*)).* Several proteins were identified, which are known to be of importance as seeds progress in maturation. Proteins belonging to this category included late embryogenesis abundant (LEA) family proteins as well as MLP-like proteins (major latex proteins).

LEAs were highly abundant in the seeds harvested at the ripe stage (Olevolosi and Acc33) and were not detected based on regulation in the seeds harvested at the mature green stage. However, they were highly abundant for the accession Olevolosi at M2 (spots 79, 91, 92, 166, 169, 175, 330 and 333, M2/M1 ratio: 1.65–44.25) and were also found in Acc 33 (spots 79, 175 and 330, M2/M1 ratio: 1.92–6.36), but were not identified within the DAS in Abuku 1 seeds. LEA proteins accumulate during late seed developmental stages and play an important role during seed drying as they confer desiccation tolerance of seeds^12,28^. However, further time points during seed ripening would have to be analysed.

Major latex-like proteins (MLP-like proteins) were also identified as DAS in all accessions (spots 203, 204 and 553). Nonetheless, only in the well germinating accessions were these proteins also found in higher abundance at the M1 stage (spot ID 553) than in M2. One MLP-like protein (MLP-like protein 43) was identified to be of major importance for drought tolerance. A knockout-mutant of *Arabidopsis thaliana* was sensitive to drought, whereas overexpression lines were shown to be drought tolerant^39^. Therefore, an increase in MLP-like protein in later stages of seed maturation might also confer desiccation tolerance in the seeds of the analysed accessions. As an MLP-like protein was already identified within the DAS in the well germinating accession in the M1 phase, this might indicate, that the well germinating accessions were already in a later stage of seed maturation, namely seed desiccation.

### Delay in seed maturation of low germinating accession Olevolosi include a postponed accumulation of PGK, GAD and glycosyl hydrolase

Phosphoglycerate kinase (PGK; E.C. 2.7.2.3) was identified in DAS of M1 seeds of Acc33 (spots 130, 131, 421) as well as in Olevolosi M2 seeds (spots 130,131, 421). Plant genomes contain 3 or more PGK genes^40^ (Fig. 5, see also Fig. 1 and GelMap online (www.gelmap.de/2700*)).* PGK is an enzyme involved not only in photosynthesis, but also plays a major role in glycolysis and gluconeogenesis^41^. It was reported, that in seeds of e.g. *Glycine max*^42^ and *Brassica napus*^43^ the abundance of PGK decreased with commencing seed maturation. Agrawal et al. (2008)^43^ stated that in the stage of seed drying, PGK abundance was very low. As a higher abundance of PGK was identified in Acc33 M1, but for the low germinating accession Olevolosi it was higher abundant in the M2 seeds, this might also display a difference in timing of seed maturations between the accessions with Oleovolosi reaching maturity later.

The identification of glutamate decarboxylase (GAD) also underlines the hypothesis, that Olevolosi seeds might take longer or are delayed in ripening. GAD in seeds acts as a metabolic link between carbon and nitrogen metabolism by catalysing the unidirectional decarboxylation of glutamate to form γ-aminobutyric acid (GABA)^44^. In African nightshade seeds, GAD was found to be higher abundant in Abuku 1 in the M1 stage and in Olevolosi in seeds of the M2 stage. Angelovici et al. (2010)^45^ recorded a shift in *Arabidopsis thaliana* seeds during transition from reserve accumulation stage to desiccation from a general decrease in unbound metabolites to the accumulation of a set of specific metabolites, including γ-aminobutyric acid. Furthermore, Fait et al. (2011)^44^ reported that GABA content is differentially regulated during the late seed maturation-to-desiccation stage, and its accumulation is indicative of a shift toward N metabolism. In *Solanum lycopersicum* berries, up-regulation of the GABA shunt led to the alteration of storage reserve accumulation and fatty acid metabolism, which are linked to seed filling^46^. However, it was reported, that in tomato fruits GABA was increased after flowering, reached a peak at the mature green berry stage and rapidly declined after the breaker stage^42,46,47^. However, if this holds true for fruits of *S. scabrum* has to be further analysed.

Taken together, the low germination in Olevolosi could be attributed to the delayed maturity in this accession as seen in the difference in the abundance of PGK and GAD which are associated with the late stages of seed maturation and that maturity can not be identified by berry colour alone in this accession.

Glycosyl hydrolase family proteins are diverse in function. However, they can function as glucosidases (EC 3.2.1.39), which are associated with germination and seed maturation^48^. Sequence analysis revealed, that Soltu.DM.06G029160.1 (spots 60, 510, 512 and 454) include putative glucosidases. A previous study in *Arabidopsis* showed that the expression of some genes in the glycosyl hydrolase family were detected in dry seeds and induced upon germination^49^. In the well germinating accessions Acc 33 and Abuku 1, glycosyl hydrolase family proteins these were highly abundant for seeds in the M1 stage. However, for the low germinating accession Olevolosi glycosyl hydrolases were also identified in the M2 stage. However, due to the scarce information on these proteins regarding the stages of seed maturation, further experiments would be needed to link these to the maturation stages in *S. scabrum*.

The well germinating accession displayed unique responses for higher abundant proteins in seeds of the M1 stage (Figs. 2 and 5 and GelMap online (www.gelmap.de/2700*)).* The eukaryotic translation factor 4A1 (eIF4A1) was found to be higher abundant only in the well-germinating accessions in M1 seeds. This protein is fundamental in gene expression. It is an ATP-dependant RNA helicase and it plays a major function in unwinding RNA secondary structure, leading to ribosomal binding and translation^50^. Further, these proteins stimulate stress-induced pathways that mediate salinity stress tolerance^51^. The overexpression of translation factor 4 A in peanut (*Arachis hypogaea*) was found to improve drought, salinity, and oxidative stress tolerance^52^. However, as eIF4A1 was only found to be higher abundant in M1 seeds, this might hint to a decline in translation activity in the M2 seeds, which would correlated with the previous described findings regarding PGK, GAD and glycosyl hydrolase, that the M2 seeds of the well germinating accessions are already in later stages of seed maturation or undergoing seed desiccation.

### Oleosin was higher abundant in seeds of the well germinating accessions in the M1 stage compared to the M2 stage

Oleosins are proteins, which are associated with oil bodies in seeds. Oil bodies are important organelles within seeds^53^ and oleosins are the most dominant protein on the oil body surface followed by caleosins and steroleosins^54^. Oleosins are specific to plants and allow the accumulation of neutral lipids that sustain seedlings during germination^55^. They are associated with desiccation tolerance in tomato seeds as they were linked to protective functions within tomato seeds^56^. Oleosins were found to accumulate during seed maturation in *A. thaliana*, however, the authors reported that for mRNA the *OLE1*, *OLE2* and *OLE3* were most abundant during the ongoing maturation, but for the protein abundance they also found OLE5 to be high in abundance per seed at the end of maturation^55^. This is in accordance with the data from this proteomic study, where the sequence identified was similar to oleosin 5 (spot 562; Soltu.DM.12G028510; oleosin 5-like). Following the Gene Onthology classification (GO classification) taken from the SpudDB database (http://spuddb.uga.edu/index.shtml), biolocial processes include post-embryonic development and reproduction (based upon the the TAIR database; https://www.arabidopsis.org/servlets/TairObject? type=locus&name=At3g01570). Within the TAIR database the expression based on the RNA-Seq data from Klepikova et al. (2016) indicate that this olesoin shows high abundance at the mature dry seeds stage of *A. thaliana*^57^. As this protein was also only identified to be higher abundant for the well germinating accessions in the M1 stage of the seeds, but was not regulated in the low germinating accession, this might be an interesting candidate to analyse at different time points in the berry and seed development of *S. scabrum*.

## Conclusion

The present study revealed that African nightshade seeds of high quality can be obtained from berries harvested at the purple ripe stage irrespective of the accession. However, for Olevolosi further indicators for maturity need to be developed, since significantly lower germination percentages were observed for Olevolosi than the commercially recommended germination rate, which was reached for Acc 33 and Abuku 1 in the M2 stage.

There was a number of differentially abundant proteins between the two stages of maturity; the mature green stage and the ripe stage, indicating metabolic differences between the two stages e.g. LEAs, MLP-like proteins, PGK, GAD, glycosyl hydrolase, eIF4A1 and oleosins. This may suggest that seeds harvested from berries of African nightshade accessions at the mature green stage were still within the early maturation phase (accumulation of storage reserves), especially in the low germinating accession, while seeds harvested from purple ripe berries were already in a later stage, i.e. the seed desiccation, in case of the two well germinating accessions. Through the proteomic analysis differences between the well and the low germinating accessions were visible, which lead to the conclusion, that the low germinating accession Olevolosi was delayed in its maturation. These findings give first proteomic insights into the seeds of this orphan crop. Further Omics methods such as metabolomics as well as increasing the number of accessions under investigation would be interesting to check seed maturation in *S. scabrum* to support the findings.

The presented data suggests that proteomic studies might also be of major importance for interesting orphan crops such as *S. scabrum* to determine optimal maturity stage of seeds for harvesting and to better understand accession-dependent variation in germination rate. Moreover, the software GelMap was expanded and now contains a reference proteome map for *S. scabrum*, which is open to be used by the scientific community to deepen seed protein knowledge in this African leafy vegetable.

## Electronic supplementary material

Below is the link to the electronic supplementary material.

Supplementary Material 1

Supplementary Material 2

Supplementary Material 3

Supplementary Material 4

Supplementary Material 5

Supplementary Material 6

Supplementary Material 7

## Data Availability

The datasets used and/or analysed during the current study available from the corresponding author on reasonable request.
