# Publisher Correction: Effect of berry maturity stages on the germination and protein constituents of African nightshade (*Solanum scabrum)* seeds

**DOI:** 10.1038/s41598-025-00689-w

**Published:** 2025-05-13

**Authors:** Noella Andenyi Ekhuya, Mary Abukutsa Onyango, Jennifer Senkler, Traud Winkelmann, Christin Bündig

**Affiliations:** 1 Department of Horticulture and Food Security, Jomo Kenyatta University of Agriculture and Technology, P.O. Box 62000, 0200 Nairobi, Kenya; 2 Institute of Plant Genetics, Leibniz Universität Hannover, Herrenhäuser Straße 2, 30419 Hannover, Germany; 3 Institute of Horticultural Production Systems, Leibniz Universität Hannover, Herrenhäuser Straße 2, 30419 Hannover, Germany

Correction to: *Scientific Reports* https://doi.org/10.1038/s41598-024-80312-6, published online 16 December 2024

The original version of this Article contained errors.

Table 3 was a duplication of Table 2.

The original Table 3 and accompanying legend appears below.

**Table 3:**
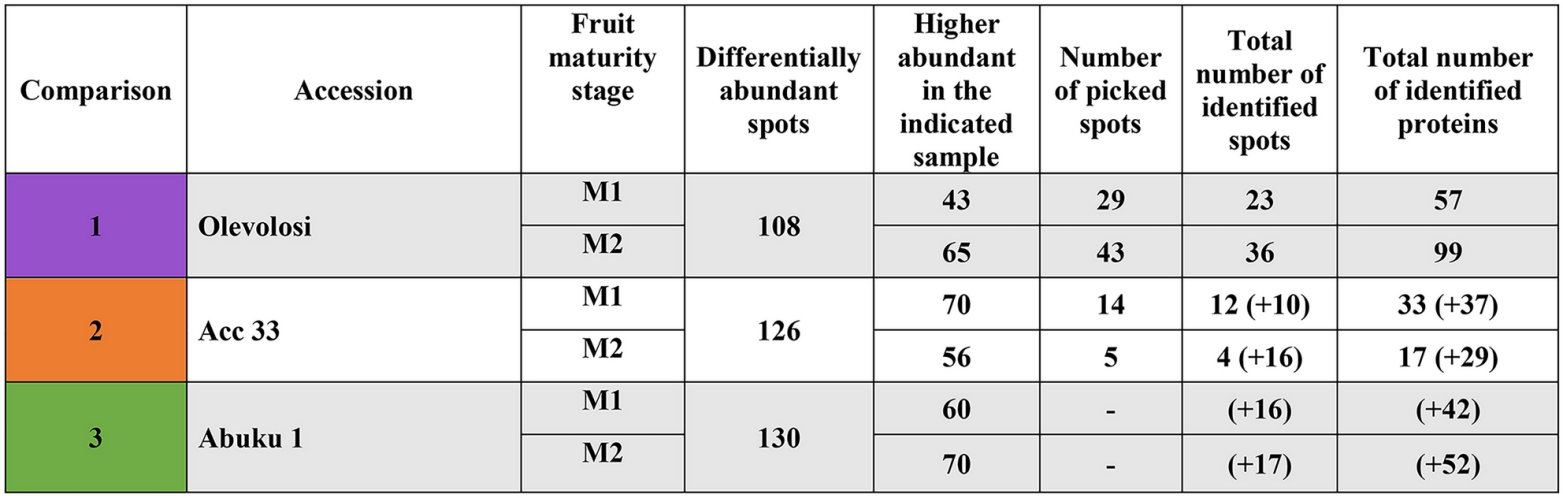
Seed storage proteins identified in the three accessions at M1 and M2 stage based on spot and protein number.

Marked in yellow: highest number of identified specific seed storage proteins among the picked spots within an accession.  = RmlC-like cupins superfamily protein;  = Cruciferin;  = Cupin family protein;  = Vicilin.

Furthermore, the Acknowledgements section was incorrect.

“The authors acknowledge Betty Orangi and Patrick Kavagi for their dedicated support during surveys, seed collection, and farm experiments.”

now reads:

“The authors are grateful for the funding within the project HORTINLEA by the German Federal Ministry of Education and Research (BMBF), grant number 031A248E, within the framework of the GlobE-Global Food Security. Our sincere thanks go to Betty Orangi and Patrick Kavagi for their dedicated support during seed collection, and farm experiments.”

The original Article has been corrected.

