# Supplementary material for: Effect of berry maturity stages on the germination and protein constituents of African nightshade (Solanum scabrum) seeds
Source: Sci Rep. 2024 Dec 16;14:30482. doi: 10.1038/s41598-024-80312-6 (PMC11649806; doi:10.1038/s41598-024-80312-6)

**Figure S1: Pictures of *S. scabrum* berries at different maturation stages and overview of berries at M2 stage on plants in fields.**


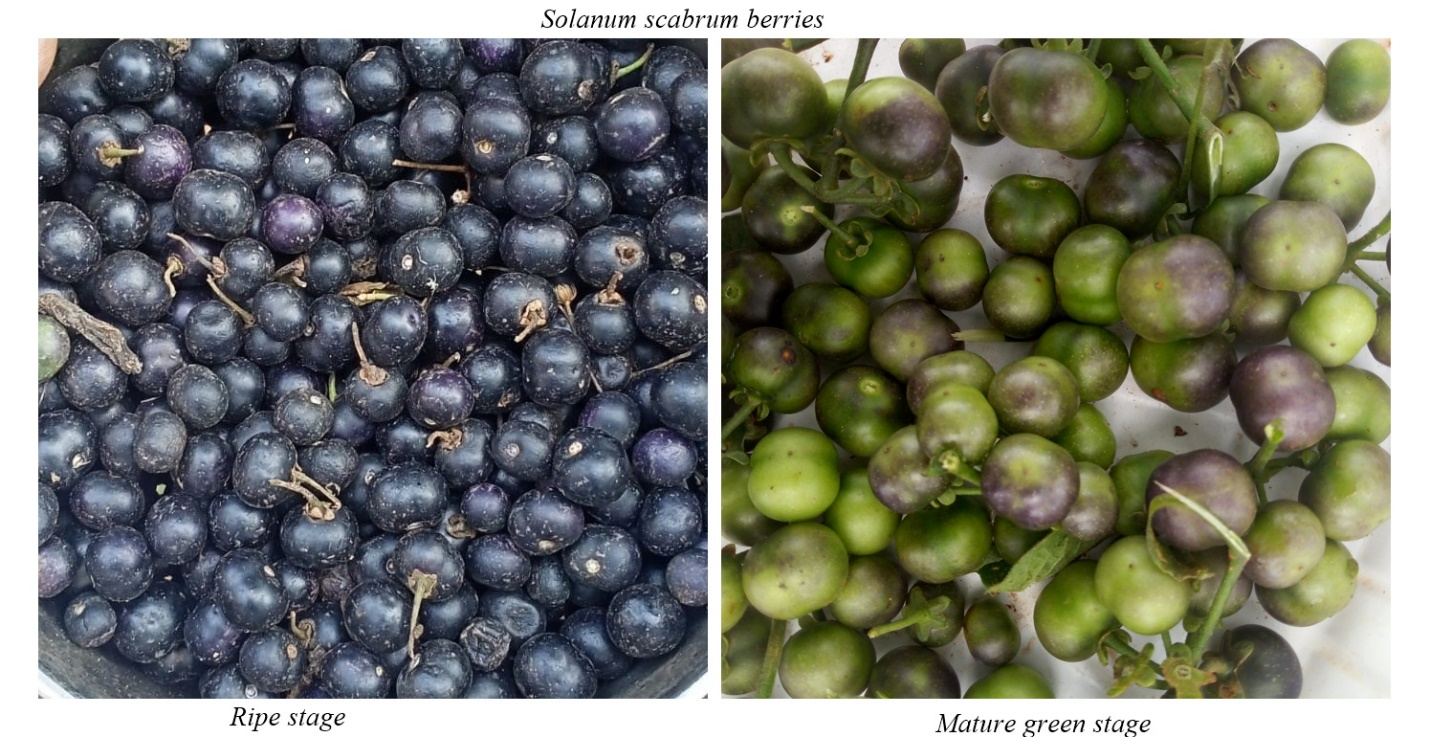


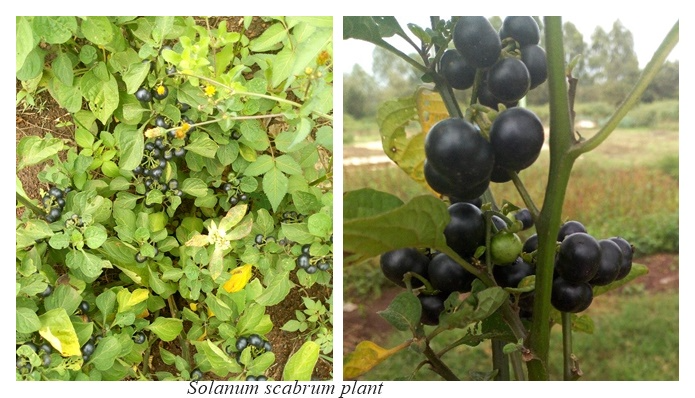

Supplement: Supplementary file 1 — Supplementary Material 1 [file 41598_2024_80312_MOESM1_ESM.docx]
