# Supplementary material for: Effect of berry maturity stages on the germination and protein constituents of African nightshade (Solanum scabrum) seeds
Source: Sci Rep. 2024 Dec 16;14:30482. doi: 10.1038/s41598-024-80312-6 (PMC11649806; doi:10.1038/s41598-024-80312-6)
Supplement: Supplementary file 2 — Supplementary Material 2 [file 41598_2024_80312_MOESM2_ESM.docx]

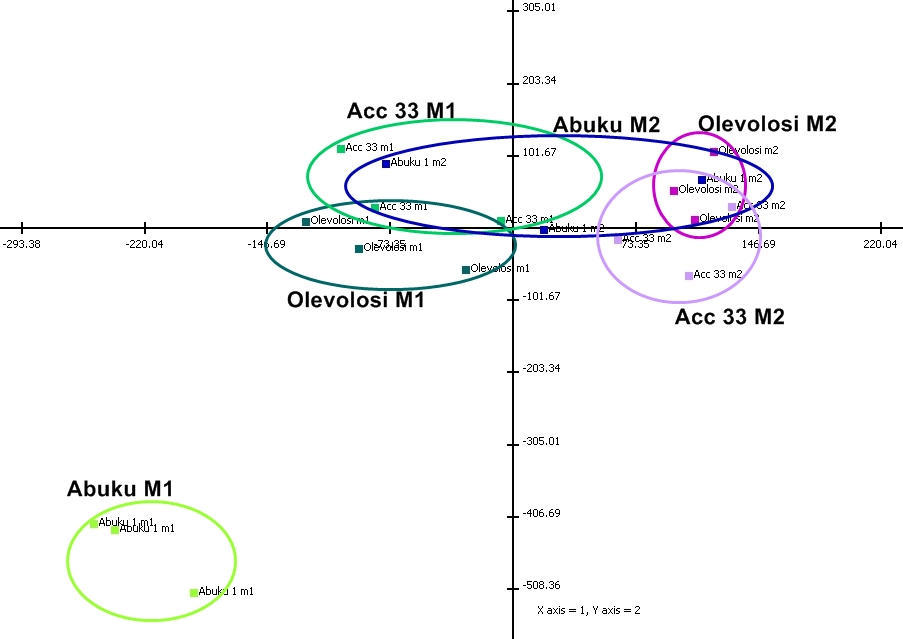


**Figure S1: 2-dimensional principal component analysis (PCA) of gels for the accessions Acc 33, Abuku 1 and Olevolosi M1 and M2. PCA was performed with the Delta2D software by DECODON**. Ellipses were drawn by hand to illustrate grouping of three gels for M1 or M2 per accession.
