# Supplementary material for: Effect of berry maturity stages on the germination and protein constituents of African nightshade (Solanum scabrum) seeds
Source: Sci Rep. 2024 Dec 16;14:30482. doi: 10.1038/s41598-024-80312-6 (PMC11649806; doi:10.1038/s41598-024-80312-6)
Supplement: Supplementary file 4 — Supplementary Material 4 [file 41598_2024_80312_MOESM4_ESM.docx]

**Supplementary table S4: Heat map of regulation (abundance in spot volume M2/M1 gels) for three accessions for seed storage proteins (table comprises all proteins found in the spots with identified seed storage proteins). Spots are ordered according to data in heatmap Figure 4.**

Only alterations of at least 1.5-fold in spot volume were considered to represent true alterations in protein level. Analysis was performed with Delta2D by DECODON. **A** = SpotID as defined by the Delta2D software from DECODON on the master gel from the 2D PAGE gels. Corresponding spots of all gels are labelled with the same ID. **B** = Accession for which the proteins in the row were identified. **C** = Proteins were identified from spots picked from the following 2D PAGE gel. Ole stands for Olevolosi, Abu stands for Abuku and Acc33 stands for Accession 33, M1 = maturity state 1 (green berries), M2 = maturity state 2 (purple berries). **D** = PGSC number from SpudDB. **E** = Functional classification mainly following the KEGG Pathway Database (++ = if no classification was automatically annotated, the proteins were manually classified) ^[1]^. **F** = The protein score obtained via the MASCOT search algorithm (www.matrixscience.com) against a potato protein database, which was based upon the sequences from *Solanum tuberosum* group Phureja DM1-3 v 6.1, which was completely sequenced by the Potato Genome Consortium 2020. **G** = Calculated PI obtained via the MASCOT search algorithm (www.matrixscience.com) against a potato protein database. **H** = Calculated MW obtained via the MASCOT search algorithm (www.matrixscience.com) against a potato protein database. **I** = Number of peptides matched to the protein through the database search. **J** = Sequence coverage in %. **K** = Unique peptides matched to the sequence. Only proteins with at least two unique peptide were considered true hits. L = Molecular weight (MW) in gel as compared to the theoretically expected MW. M: MW in gel corresponding to the theoretically expected MW ± 15 kDa, S: MW in gel lower than theoretically expected. **L**: MW in gel larger than theoretically expected. **M** = Mean relative spot volume obtained according to three gels of M1 seeds or M2 seeds illustrated by graphs. The first bar (orange) represents the mean normalized spot volume in the gels of M1 seeds of Accession 33. The second bar (light orange) represents the mean normalized spot volume in the gels of the M2 seeds from Accession 33. The third bar (green) stands for the mean normalized spot volume in the gels of the M1 seeds of Abuku 1. The fourth bar (light green) represents the mean normalized spot volume in the gels of the M2 seeds of Abuku 1. The fifth bar (purple) represents the mean normalized spot volume in the gels of the M1 seeds from Olevolosi. The sixth bar (light purple) stands for the mean normalized spot volume in the gels of the M2 seeds of Olevolosi.

| **Spot ID^A^** | **Significant in Accession^B^** | **Protein name^C^** | **PGSC numbers^D^** | **KEGG^E^** | **Score^F^** | **PI Calc^G^** | **MW Calc ^H^** | **Pep ^I^** | **SC [%]^J^** | **UPep^K^** | **Size^L^** | **Normalized spot volume^M^** |
| --- | --- | --- | --- | --- | --- | --- | --- | --- | --- | --- | --- | --- |
| **421** | **Olevolosi-M2**  **Acc 33 M1** | **Phosphoglycerate kinase** | **Soltu.DM.07G028580.1** | **Metabolism – Glycolysis ++** | **1162** | **5.4** | **42.3** | **45** | **53.9** | **19** | **L** | **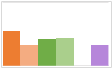** |
|  |  | **Hydroxysteroid dehydrogenase** | **Soltu.DM.06G021080.1** | **Biological processes – Growth and seed production ++** | **381** | **7.8** | **42.9** | **9** | **21.6** | **7** | **L** |  |
|  |  | **RmlC-like cupins superfamily protein** | **Soltu.DM.09G021450.1** | **Seed storage protein ++** | **90** | **6.1** | **49.9** | **3** | **3.4** | **2** | **L** |  |
| **162** | **Olevolosi-M2** | **RmlC-like cupins superfamily protein** | **Soltu.DM.11G025490.1** | **Seed storage protein ++** | **184** | **5.6** | **57.0** | **6** | **6.7** | **3** | **M** | **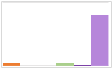** |
| **130** | **Olevolosi-M2**  **Acc 33 M1** | **Phosphoglycerate kinase** | **Soltu.DM.07G028580.1** | **Metabolism – Glycolysis ++** | **839** | **5.4** | **42.3** | **32** | **47.6** | **15** | **L** | **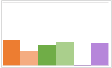** |
|  |  | **Hydroxysteroid dehydrogenase** | **Soltu.DM.06G021080.1** | **Biological processes – Growth and seed production ++** | **250** | **7.8** | **42.9** | **7** | **16.1** | **5** | **L** |  |
|  |  | **RmlC-like cupins superfamily protein** | **Soltu.DM.09G021500.1** | **Seed storage protein ++** | **74** | **7.0** | **54.4** | **5** | **3.7** | **2** | **L** |  |
| **426** | **Olevolosi-M2** | **Protein of unknown function (DUF1264)** | **Soltu.DM.09G031240.1** | **Unknown ++** | **641** | **5.7** | **26.9** | **55** | **41.2** | **10** | **L** | **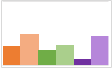** |
|  |  | **RmlC-like cupins superfamily protein** | **Soltu.DM.09G021450.1** | **Seed storage protein ++** | **52** | **6.1** | **49.9** | **2** | **3.4** | **2** | **M** |  |
| **210** | **Olevolosi-M2** | **Cruciferin** | **Soltu.DM.03G000650.1** | **Storage protein ++** | **108** | **7.9** | **42.6** | **5** | **2.4** | **2** | **M** | **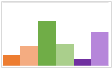** |
| **380** | **Olevolosi-M2** | **RmlC-like cupins superfamily protein** | **Soltu.DM.09G021460.1** | **Seed storage protein ++** | **371** | **7.9** | **53.4** | **26** | **18.1** | **5** | **M** | **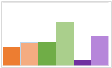** |
|  |  | **RmlC-like cupins superfamily protein** | **Soltu.DM.09G021500.1** | **Seed storage protein ++** | **280** | **7.0** | **54.4** | **18** | **12.7** | **5** | **M** |  |
| **239** | **Olevolosi-M2**  **Acc 33 M2** | **RmlC-like cupins superfamily protein** | **Soltu.DM.03G000660.1** | **Seed storage protein ++** | **158** | **9.6** | **14.7** | **9** | **13.8** | **3** | **M** | **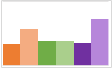** |
| **178** | **Olevolosi-M2**  **Abuku M1** | **RmlC-like cupins superfamily protein** | **Soltu.DM.09G021500.1** | **Seed storage protein ++** | **101** | **7.0** | **54.4** | **8** | **3.7** | **2** | **M** | **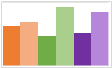** |
|  |  | **Cupin family protein** | **Soltu.DM.09G024720.1** | **Seed storage protein ++** | **65** | **8.6** | **65.7** | **3** | **4.1** | **2** | **S** |  |
|  |  | **RmlC-like cupins superfamily protein** | **Soltu.DM.03G000660.1** | **Seed storage protein ++** | **43** | **9.6** | **14.7** | **3** | **12.2** | **2** | **L** |  |
| **400** | **Acc33-M2**  **Abuku M2** | **Cruciferin** | **Soltu.DM.09G026760.1** | **Seed storage protein ++** | **291** | **6.7** | **58.1** | **8** | **7.4** | **3** | **M** | **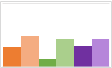** |
|  |  | **RmlC-like cupins superfamily protein** | **Soltu.DM.03G000660.1** | **Seed storage protein ++** | **130** | **9.6** | **14.7** | **8** | **13.8** | **3** | **L** |  |
|  |  | **RmlC-like cupins superfamily protein** | **Soltu.DM.11G025490.1** | **Seed storage protein ++** | **106** | **5.6** | **57.0** | **3** | **4.5** | **2** | **M** |  |
|  |  | **RmlC-like cupins superfamily protein** | **Soltu.DM.09G021500.1** | **Seed storage protein ++** | **99** | **7.0** | **54.4** | **10** | **3.7** | **2** | **M** |  |
|  |  | **1-cysteine peroxiredoxin** | **Soltu.DM.03G013100.1** | **Biological processes – ROS detoxification++** | **86** | **6.1** | **24.2** | **3** | **8.2** | **2** | **L** |  |
| **98** | **Olevolosi-M2** | **RmlC-like cupins superfamily protein** | **Soltu.DM.09G021500.1** | **Seed storage protein ++** | **143** | **7.0** | **54.4** | **11** | **5.0** | **3** | **L** | **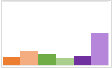** |
| **154** | **Olevolosi-M2** | **RmlC-like cupins superfamily protein** | **Soltu.DM.09G021460.1** | **Seed storage protein ++** | **299** | **7.9** | **53.4** | **16** | **12.4** | **4** | **M** | **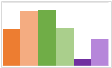** |
|  |  | **RmlC-like cupins superfamily protein** | **Soltu.DM.03G000660.1** | **Seed storage protein ++** | **167** | **9.6** | **14.7** | **11** | **13.8** | **3** | **L** |  |
|  |  | **Cruciferin** | **Soltu.DM.03G000650.1** | **Storage protein ++** | **121** | **7.9** | **42.6** | **5** | **2.4** | **2** | **M** |  |
|  |  | **RmlC-like cupins superfamily protein** | **Soltu.DM.09G021450.1** | **Seed storage protein ++** | **85** | **6.1** | **49.9** | **2** | **3.4** | **2** | **M** |  |
|  |  | **Cruciferin** | **Soltu.DM.09G026760.1** | **Seed storage protein ++** | **54** | **6.7** | **58.1** | **3** | **2.3** | **2** | **M** |  |
| **208** | **Olevolosi-M2**  **Acc 33 M2** | **Cruciferin** | **Soltu.DM.03G000650.1** | **Storage protein ++** | **120** | **7.9** | **42.6** | **5** | **2.4** | **2** | **M** | **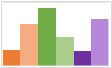** |
|  |  | **RmlC-like cupins superfamily protein** | **Soltu.DM.09G021450.1** | **Seed storage protein ++** | **101** | **6.1** | **49.9** | **3** | **3.4** | **2** | **M** |  |
| **425** | **Olevolosi-M2**  **Acc 33 M2** | **Cruciferin** | **Soltu.DM.09G026760.1** | **Seed storage protein ++** | **442** | **6.7** | **58.1** | **38** | **11.5** | **7** | **L** | **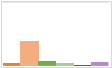** |
|  |  | **mitochondrial lipoamide dehydrogenase** | **Soltu.DM.05G023290.1** | **Metabolism - Carbohydrate metabolism - Glycolysis / Gluconeogenesis** | **54** | **7.1** | **52.8** | **2** | **3.6** | **2** | **L** |  |
| **292** | **Olevolosi-M2**  **Acc 33 M2** | **Cupin family protein** | **Soltu.DM.09G020060.1** | **Seed storage protein ++** | **280** | **9.3** | **53.5** | **33** | **12.0** | **7** | **L** | **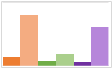** |
|  |  | **Cupin family protein** | **Soltu.DM.09G024710.1** | **Seed storage protein ++** | **104** | **5.9** | **42.5** | **5** | **5.6** | **2** | **L** |  |
|  |  | **Cupin family protein** | **Soltu.DM.09G024720.1** | **Seed storage protein ++** | **72** | **8.6** | **65.7** | **2** | **4.8** | **2** | **L** |  |
| **328** | **Olevolosi-M2** | **Cruciferin** | **Soltu.DM.09G026760.1** | **Seed storage protein ++** | **246** | **6.7** | **58.1** | **7** | **8.5** | **4** | **M** | **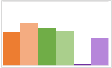** |
|  |  | **RmlC-like cupins superfamily protein** | **Soltu.DM.03G000660.1** | **Seed storage protein ++** | **174** | **9.6** | **14.7** | **11** | **13.8** | **3** | **L** |  |
|  |  | **Cruciferin** | **Soltu.DM.03G000650.1** | **Storage protein ++** | **101** | **7.9** | **42.6** | **4** | **4.7** | **2** | **L** |  |
|  |  | **RmlC-like cupins superfamily protein** | **Soltu.DM.09G021500.1** | **Seed storage protein ++** | **100** | **7.0** | **54.4** | **5** | **5.0** | **3** | **M** |  |
|  |  | **Pyridoxine biosynthesis 1.2** | **Soltu.DM.03G034640.1** | **Metabolism - Metabolism of cofactors and vitamins - Vitamin B6 metabolism** | **92** | **5.2** | **32.7** | **5** | **9.8** | **3** | **L** |  |
| **322** | **Olevolosi-M2**  **Abuku M1** | **Cruciferin** | **Soltu.DM.09G026760.1** | **Seed storage protein ++** | **347** | **6.7** | **58.1** | **21** | **11.5** | **6** | **M** | **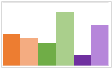** |
| **97** | **Acc33-M1**  **Abuku M2** | **ATP synthase alpha/beta family protein** | **Soltu.DM.05G004470.1** | **Metabolism - Energy metabolism** | **717** | **5.6** | **59.5** | **27** | **22.8** | **11** | **L** | **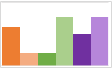** |
|  |  | **Cruciferin** | **Soltu.DM.09G026760.1** | **Seed storage protein ++** | **84** | **6.7** | **58.1** | **4** | **3.3** | **2** | **L** |  |
|  |  | **S-adenosyl-L-homocysteine hydrolase** | **Soltu.DM.09G029630.1** | **Metabolism - Amino acid metabolism - Cysteine and methionine metabolism** | **77** | **5.6** | **53.2** | **3** | **2.9** | **2** | **L** |  |
| **194** | **Acc33-M1**  **Abuku M2** | **Cruciferin** | **Soltu.DM.03G000650.1** | **Seed storage protein ++** | **214** | **7.9** | **42.6** | **6** | **13.4** | **5** | **M** | **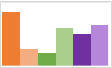** |
|  |  | **RmlC-like cupins superfamily protein** | **Soltu.DM.09G021500.1** | **Seed storage protein ++** | **142** | **7.0** | **54.4** | **8** | **6.2** | **3** | **M** |  |
|  |  | **RmlC-like cupins superfamily protein** | **Soltu.DM.09G021460.1** | **Seed storage protein ++** | **106** | **7.9** | **53.4** | **4** | **4.0** | **2** | **M** |  |
| **381** | **Acc33-M1**  **Abuku M2** | **Protein phosphatase 2C** | **Soltu.DM.07G024120.1** | **Protein families: metabolism -Protein phosphatases and associated proteins** | **288** | **5.6** | **30.9** | **10** | **17.7** | **5** | **5** | **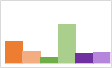** |
|  |  | **Vicilin** | **Soltu.DM.11G025490.1** | **Seed storage protein ++** | **270** | **5.6** | **57.0** | **6** | **11.2** | **5** | **5** |  |
|  |  | **RmlC-like cupins superfamily protein** | **Soltu.DM.09G021500.1** | **Seed storage protein ++** | **165** | **7.0** | **54.4** | **8** | **7.9** | **3** | **2** |  |
| **137** | **Olevolosi-M1** | **Cruciferin** | **Soltu.DM.09G026760.1** | **Seed storage protein ++** | **106** | **6.7** | **58.1** | **3** | **3.9** | **2** | **L** | **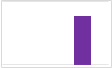** |
|  |  | **RmlC-like cupins superfamily protein** | **Soltu.DM.09G021450.1** | **Seed storage protein ++** | **84** | **6.1** | **49.9** | **3** | **3.4** | **2** | **L** |  |
|  |  | **Glyceraldehyde-3-phosphate dehydrogenase C subunit** | **Soltu.DM.03G024400.1** | **Metabolism - Carbohydrate metabolism - Glycolysis / Gluconeogenesis ++** | **41** | **7.7** | **36.7** | **2** | **4.5** | **2** | **L** |  |
| **158** | **Olevolosi-M1** | **RmlC-like cupins superfamily protein** | **Soltu.DM.11G025490.1** | **Seed storage protein ++** | **143** | **5.6** | **57.0** | **5** | **6.7** | **3** | **M** | **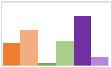** |
| **548** | **Olevolosi-M1** | **RmlC-like cupins superfamily protein** | **Soltu.DM.09G021500.1** | **Seed storage protein ++** | **180** | **7.0** | **54.4** | **5** | **10.0** | **3** | **M** | **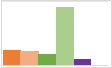** |
|  |  | **Cruciferin** | **Soltu.DM.09G026760.1** | **Seed storage protein ++** | **66** | **6.7** | **58.1** | **5** | **3.7** | **2** | **S** |  |
| **382** | **Olevolosi-M2**  **Abuku M1** | **RmlC-like cupins superfamily protein** | **Soltu.DM.09G021460.1** | **Seed storage protein ++** | **333** | **7.9** | **53.4** | **23** | **12.4** | **4** | **M** | **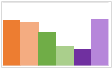** |
|  |  | **RmlC-like cupins superfamily protein** | **Soltu.DM.09G021500.1** | **Seed storage protein ++** | **215** | **7.0** | **54.4** | **12** | **10.8** | **4** | **M** |  |
| **510** | **Olevolosi-M2**  **Acc 33 M1** | **Glycosyl hydrolase family protein** | **Soltu.DM.06G029150.1** | **Metabolism - Hydrolases –Glycosylases- [EC 3.2.1.21] ++** | **276** | **5.7** | **55.9** | **11** | **10.9** | **5** | **L** | **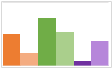** |
|  |  | **Glycosyl hydrolase family protein** | **Soltu.DM.06G029150.2** | **Metabolism - Hydrolases –Glycosylases- [EC 3.2.1.21] ++** | **268** | **6.9** | **66.0** | **14** | **9.2** | **5** | **L** |  |
|  |  | **GTP binding Elongation factor Tu family protein** | **Soltu.DM.06G005560.1** | **Genetic Information Processing - Translation - RNA transport and biogenesis** | **184** | **9.4** | **35.7** | **6** | **12.0** | **4** | **L** |  |
|  |  | **Glycosyl hydrolase family protein** | **Soltu.DM.06G029160.1** | **Metabolism - Hydrolases –Glycosylases- [EC 3.2.1.21] ++** | **173** | **8.5** | **68.7** | **9** | **5.9** | **3** | **L** |  |
|  |  | **RmlC-like cupins superfamily protein** | **Soltu.DM.09G021500.1** | **Seed storage protein ++** | **89** | **7.0** | **54.4** | **4** | **6.2** | **2** | **L** |  |
| **131** | **Olevolosi-M2**  **Acc 33 M1** | **Actin** | **Soltu.DM.11G008990.1** | **Signaling and cellular processes - Cytoskeleton proteins** | **683** | **5.2** | **41.8** | **30** | **31.3** | **11** | **L** | **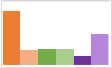** |
|  |  | **Actin** | **Soltu.DM.03G011750.1** | **Signaling and cellular processes - Cytoskeleton proteins** | **661** | **5.2** | **41.7** | **28** | **31.3** | **11** | **L** |  |
|  |  | **Actin-11** | **Soltu.DM.04G007480.1** | **Signaling and cellular processes - Cytoskeleton proteins** | **488** | **5.2** | **41.6** | **23** | **23.6** | **9** | **L** |  |
|  |  | **Phosphoglycerate kinase** | **Soltu.DM.07G028580.1** | **Metabolism – Glycolysis ++** | **156** | **5.4** | **42.3** | **2** | **6.7** | **2** | **L** |  |
|  |  | **Hydroxysteroid dehydrogenase** | **Soltu.DM.06G021080.1** | **Biological processes – Growth and seed production ++** | **95** | **7.8** | **42.9** | **4** | **5.7** | **2** | **L** |  |
|  |  | **RmlC-like cupins superfamily protein** | **Soltu.DM.09G021500.1** | **Seed storage protein ++** | **94** | **7.0** | **54.4** | **4** | **5.0** | **3** | **L** |  |
| **491** | **Olevolosi-M2**  **Abuku M1** | **Cupin family protein** | **Soltu.DM.09G024720.1** | **Seed storage protein ++** | **207** | **8.6** | **65.7** | **20** | **7.6** | **6** | **M** | **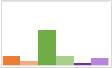** |
|  |  | **Cupin family protein** | **Soltu.DM.09G024710.1** | **Seed storage protein ++** | **136** | **5.9** | **42.5** | **6** | **5.6** | **2** | **L** |  |
| **95** | **Olevolosi-M2**  **Abuku M1** | **RmlC-like cupins superfamily protein** | **Soltu.DM.09G021500.1** | **Seed storage protein ++** | **155** | **7.0** | **54.4** | **12** | **7.3** | **4** | **L** | **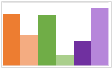** |
|  |  | **RmlC-like cupins superfamily protein** | **Soltu.DM.03G000660.1** | **Seed storage protein ++** | **125** | **9.6** | **14.7** | **4** | **13.8** | **3** | **L** |  |
|  |  | **Glutamate decarboxylase** | **Soltu.DM.03G019160.3** | **Metabolism - Carbohydrate metabolism - Amino acid metabolism** | **117** | **5.7** | **56.7** | **5** | **6.0** | **3** | **L** |  |
|  |  | **Cruciferin** | **Soltu.DM.09G026760.1** | **Seed storage protein ++** | **94** | **6.7** | **58.1** | **4** | **3.9** | **2** | **L** |  |
|  |  | **Cupin family protein** | **Soltu.DM.09G024720.1** | **Seed storage protein ++** | **77** | **8.6** | **65.7** | **3** | **3.5** | **2** | **L** |  |
|  |  | **DNA binding** | **Soltu.DM.11G004880.1** | **Unknown ++** | **63** | **4.7** | **56.6** | **4** | **1.7** | **2** | **L** |  |
|  |  | **RmlC-like cupins superfamily protein** | **Soltu.DM.09G021460.1** | **Seed storage protein ++** | **53** | **7.9** | **53.4** | **3** | **4.0** | **2** | **L** |  |
|  |  | **Structural maintenance of chromosomes (SMC) family protein** | **Soltu.DM.06G034250.1** | **Genetic information processing - Chromosome and associated proteins** | **45** | **9.1** | **141.0** | **3** | **1.3** | **2** | **S** |  |
| **334** | **Olevolosi-M1**  **Abuku M1** | **RmlC-like cupins superfamily protein** | **Soltu.DM.09G021500.1** | **Seed storage protein ++** | **77** | **7.0** | **54.4** | **4** | **7.9** | **3** | **L** | **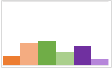** |
| **475** | **Acc33-M2**  **Abuku M1** | **Cupin family protein** | **Soltu.DM.09G024720.1** | **Seed storage protein ++** | **264** | **8.6** | **65.7** | **14** | **8.6** | **6** | **L** | **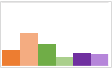** |
|  |  | **Cupin family protein** | **Soltu.DM.09G024710.1** | **Seed storage protein ++** | **121** | **5.9** | **42.5** | **5** | **5.6** | **2** | **L** |  |
| **71** | **Olevolosi-M1** | **RmlC-like cupins superfamily protein** | **Soltu.DM.09G021500.1** | **Seed storage protein ++** | **232** | **7.0** | **54.4** | **10** | **10.8** | **4** | **L** | **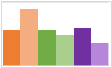** |
|  |  | **Succinate dehydrogenase 1-1** | **Soltu.DM.02G025210.1** | **Metabolism -Carbohydrate metabolism -Citrate cycle (TCA cycle)** | **185** | **6.1** | **69.2** | **4** | **6.5** | **4** | **L** |  |
|  |  | **Thiamine pyrophosphate dependent pyruvate decarboxylase family protein** | **Soltu.DM.10G019450.1** | **Metabolism - Carbohydrate metabolism - Glycolysis / Gluconeogenesis** | **126** | **5.7** | **65.5** | **6** | **5.0** | **3** | **L** |  |
| **365** | **Olevolosi-M1**  **Abuku M1** | **RmlC-like cupins superfamily protein** | **Soltu.DM.09G021500.1** | **Seed storage protein ++** | **375** | **7.0** | **54.4** | **27** | **18.3** | **6** | **M** | **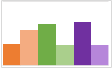** |
| **370** | **Olevolosi-M1**  **Abuku M2** | **RmlC-like cupins superfamily protein** | **Soltu.DM.09G021500.1** | **Seed storage protein ++** | **265** | **7.0** | **54.4** | **15** | **15.4** | **5** | **M** | **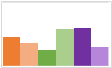** |
|  |  | **NAD(P)-binding Rossmann-fold superfamily protein** | **Soltu.DM.01G038420.1** | **Genetic information processing - Ubiquitin system ++** | **149** | **9.4** | **37.3** | **6** | **11.7** | **3** | **L** |  |
|  |  | **RmlC-like cupins superfamily protein** | **Soltu.DM.09G021450.1** | **Seed storage protein ++** | **143** | **6.1** | **49.9** | **12** | **7.9** | **5** | **M** |  |
| **84** | **Olevolosi-M1** | **Phosphoglycerate mutase, 2,3-bisphosphoglycerate-independent** | **Soltu.DM.07G014610.1** | **Metabolism - Carbohydrate metabolism - Glycolysis / Gluconeogenesis ++** | **176** | **5.3** | **61.2** | **6** | **7.5** | **5** | **L** | **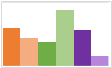** |
|  |  | **Heat-shock protein 70T-2** | **Soltu.DM.09G024280.1** | **Genetic information processing - Chaperones and folding catalysts** | **150** | **5.5** | **62.4** | **6** | **8.6** | **4** | **L** |  |
|  |  | **RmlC-like cupins superfamily protein** | **Soltu.DM.09G021460.1** | **Seed storage protein ++** | **100** | **7.9** | **53.4** | **3** | **4.6** | **2** | **L** |  |
|  |  | **RmlC-like cupins superfamily protein** | **Soltu.DM.09G021450.1** | **Seed storage protein ++** | **85** | **6.1** | **49.9** | **3** | **3.4** | **2** | **L** |  |
|  |  | **Thiamine pyrophosphate dependent pyruvate decarboxylase family protein** | **Soltu.DM.10G019450.1** | **Metabolism - Carbohydrate metabolism - Glycolysis / Gluconeogenesis** | **82** | **5.7** | **65.5** | **2** | **3.0** | **2** | **L** |  |
| **72** | **Olevolosi-M1**  **Abuku M2** | **RmlC-like cupins superfamily protein** | **Soltu.DM.09G021500.1** | **Seed storage protein ++** | **156** | **7.0** | **54.4** | **6** | **7.9** | **3** | **L** | **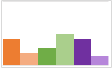** |
|  |  | **Thiamine pyrophosphate dependent pyruvate decarboxylase family protein** | **Soltu.DM.10G019450.1** | **Metabolism - Carbohydrate metabolism - Glycolysis / Gluconeogenesis** | **147** | **5.7** | **65.5** | **8** | **5.0** | **3** | **L** |  |
|  |  | **RmlC-like cupins superfamily protein** | **Soltu.DM.03G000660.1** | **Seed storage protein ++** | **110** | **9.6** | **14.7** | **6** | **12.2** | **2** | **L** |  |
|  |  | **Cruciferin** | **Soltu.DM.03G000650.1** | **Storage protein ++** | **86** | **7.9** | **42.6** | **5** | **2.4** | **2** | **L** |  |
|  |  | **RmlC-like cupins superfamily protein** | **Soltu.DM.09G021460.1** | **Seed storage protein ++** | **83** | **7.9** | **53.4** | **5** | **4.6** | **2** | **L** |  |
|  |  | **Cruciferin** | **Soltu.DM.09G026760.1** | **Seed storage protein ++** | **78** | **6.7** | **58.1** | **3** | **3.9** | **2** | **L** |  |
| **75** | **Olevolosi-M1** | **Thiamine pyrophosphate dependent pyruvate decarboxylase family protein** | **Soltu.DM.10G019450.1** | **Metabolism - Carbohydrate metabolism - Glycolysis / Gluconeogenesis** | **197** | **5.7** | **65.5** | **9** | **6.3** | **4** | **L** | **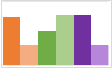** |
|  |  | **RmlC-like cupins superfamily protein** | **Soltu.DM.09G021500.1** | **Seed storage protein ++** | **112** | **7.0** | **54.4** | **3** | **6.7** | **2** | **L** |  |
| **354** | **Olevolosi-M1** | **Glyceraldehyde-3-phosphate dehydrogenase C2** | **Soltu.DM.05G010790.1** | **Metabolism - Carbohydrate metabolism - Glycolysis / Gluconeogenesis ++** | **599** | **6.4** | **36.6** | **24** | **29.3** | **12** | **L** | **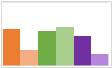** |
|  |  | **Glyceraldehyde-3-phosphate dehydrogenase C subunit** | **Soltu.DM.06G027160.1** | **Metabolism - Carbohydrate metabolism - Glycolysis / Gluconeogenesis ++** | **401** | **7.7** | **36.6** | **17** | **22.8** | **10** | **L** |  |
|  |  | **RmlC-like cupins superfamily protein** | **Soltu.DM.09G021500.1** | **Seed storage protein ++** | **78** | **7.0** | **54.4** | **3** | **3.1** | **2** | **L** |  |
| **10** | **Acc33-M1**  **Abuku M1** | **Heat shock protein** | **Soltu.DM.03G029350.1** | **Genetic information processing - Chaperones and folding catalysts** | **1503** | **5.8** | **101.1** | **56** | **34.0** | **29** | **L** | **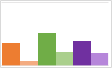** |
|  |  | **Casein lytic proteinase B3** | **Soltu.DM.02G031120.1** | **Genetic information processing - Chaperones and folding catalysts ++** | **184** | **6.1** | **110.3** | **11** | **4.4** | **4** | **L** |  |
|  |  | **RmlC-like cupins superfamily protein** | **Soltu.DM.09G021500.1** | **Seed storage protein ++** | **143** | **7.0** | **54.4** | **9** | **5.0** | **3** | **L** |  |
| **359** | **Olevolosi-M1** | **RmlC-like cupins superfamily protein** | **Soltu.DM.09G021500.1** | **Seed storage protein ++** | **104** | **7.0** | **54.4** | **8** | **7.9** | **3** | **L** | **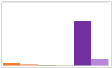** |
|  |  | **Lactate/malate dehydrogenase family protein** | **Soltu.DM.09G026740.1** | **Metabolism – citric acid cycle++** | **85** | **5.9** | **35.4** | **5** | **7.2** | **3** | **L** |  |
|  |  | **NAD(P)-binding Rossmann-fold superfamily protein** | **Soltu.DM.01G038420.1** | **Genetic information processing - Ubiquitin system ++** | **66** | **9.4** | **37.3** | **3** | **7.9** | **2** | **L** |  |
| **406** | **Olevolosi-M1** | **RmlC-like cupins superfamily protein** | **Soltu.DM.11G025490.1** | **Seed storage protein ++** | **118** | **5.6** | **57.0** | **4** | **3.9** | **2** | **M** | **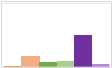** |
| **360** | **Acc33-M2**  **Abuku M1** | **Cruciferin** | **Soltu.DM.09G026760.1** | **Seed storage protein ++** | **358** | **6.7** | **58.1** | **17** | **11.5** | **6** | **M** | **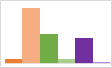** |
|  |  | **NAD(P)-linked oxidoreductase superfamily protein** | **Soltu.DM.09G009380.1** | **Metabolism - Glycolysis - Gluconeogenesis** | **337** | **6.1** | **58.1** | **14** | **20.4** | **7** | **M** |  |
|  |  | **Annexin** | **Soltu.DM.04G029320.1** | **Environmental Information Processing ++** | **262** | **5.3** | **36.3** | **9** | **12.7** | **5** | **L** |  |
|  |  | **NAD(P)-binding Rossmann-fold superfamily protein** | **Soltu.DM.01G038420.1** | **Genetic information processing - Ubiquitin system ++** | **160** | **9.4** | **35.8** | **3** | **10.6** | **3** | **L** |  |
|  |  | **Hydroxysteroid dehydrogenase** | **Soltu.DM.06G028000.1** | **Biological processes – Growth and seed production ++** | **154** | **5.6** | **37.3** | **7** | **12.0** | **5** | **L** |  |
|  |  | **RmlC-like cupins superfamily protein** | **Soltu.DM.03G000660.1** | **Seed storage protein ++** | **144** | **9.6** | **38.7** | **7** | **13.8** | **3** | **L** |  |
|  |  | **RmlC-like cupins superfamily protein** | **Soltu.DM.09G021500.1** | **Seed storage protein ++** | **105** | **7.0** | **14.7** | **8** | **3.7** | **2** | **L** |  |
|  |  | **P-loop containing nucleoside triphosphate hydrolases superfamily protein** | **Soltu.DM.03G014080.1** | **Genetic Information Processing – Translation - Ribosome biogenesis in eukaryotes** | **38** | **9.1** | **54.4** | **2** | **1.2** | **2** | **L** |  |
| **394** | **Olevolosi-M1** | **RmlC-like cupins superfamily protein** | **Soltu.DM.11G025490.1** | **Seed storage protein ++** | **166** | **5.6** | **57.0** | **8** | **6.9** | **3** | **M** | **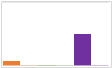** |
|  |  | **Triosephosphate isomerase** | **Soltu.DM.04G007490.1** | **Metabolism - Carbohydrate metabolism - Glycolysis / Gluconeogenesis ++** | **103** | **5.7** | **27.0** | **2** | **8.7** | **2** | **L** |  |
|  |  | **20S proteasome alpha subunit G1** | **Soltu.DM.10G026260.1** | **Genetic Information Processing - Folding, sorting and degradation - Proteasome** | **73** | **6.1** | **27.1** | **3** | **7.6** | **2** | **L** |  |
| **423** | **Olevolosi-M1** | **glyceraldehyde-3-phosphate dehydrogenase C2** | **Soltu.DM.05G010790.1** | **Metabolism - Carbohydrate metabolism - Glycolysis / Gluconeogenesis ++** | **454** | **6.4** | **36.6** | **23** | **25.4** | **11** | **L** | **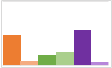** |
|  |  | **RmlC-like cupins superfamily protein** | **Soltu.DM.09G021450.1** | **Seed storage protein ++** | **78** | **6.1** | **49.9** | **2** | **3.4** | **2** | **L** |  |
| **309** | **Olevolosi-M1** | **Cupin family protein** | **Soltu.DM.09G024720.1** | **Seed storage protein ++** | **215** | **8.6** | **65.7** | **5** | **6.0** | **4** | **L** | **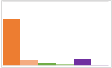** |
|  |  | **Cupin family protein** | **Soltu.DM.09G024710.1** | **Seed storage protein ++** | **132** | **5.9** | **42.5** | **5** | **5.6** | **2** | **L** |  |
| **411** | **Olevolosi-M1**  **Abuku M1** | **Cruciferin** | **Soltu.DM.09G026760.1** | **Seed storage protein ++** | **168** | **6.7** | **58.1** | **9** | **6.4** | **3** | **L** | **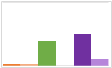** |

# Refrences

1. Kanehisa, M., Furumichi, M., Sato, Y., Kawashima, M. & Ishiguro-Watanabe, M. KEGG for taxonomy-based analysis of pathways and genomes. *Nucleic Acids Res.* **51**, D587–D592 (2023).
