# Supplementary material for: Effect of berry maturity stages on the germination and protein constituents of African nightshade (Solanum scabrum) seeds
Source: Sci Rep. 2024 Dec 16;14:30482. doi: 10.1038/s41598-024-80312-6 (PMC11649806; doi:10.1038/s41598-024-80312-6)
Supplement: Supplementary file 5 — Supplementary Material 5 [file 41598_2024_80312_MOESM5_ESM.docx]

**Supplemntary table S5: Further interesting spots identified for accession Olevolosi, Acc 33 and Abuku 1 for maturity stages M1 and M2, respectively. Data are ordered according to heatmap in Figure 5.**

Only alterations of at least 1.5-fold in spot volume were considered to represent true alterations in protein level. Analysis was performed with Delta2D by DECODON. **A** = SpotID as defined by the Delta2D software from DECODON on the master gel from the 2D PAGE gels. Corresponding spots of all gels are labelled with the same ID. **B** = Accession for which the proteins in the row were identified. **C** = Proteins were identified from spots picked from the following 2D PAGE gel. Ole stands for Olevolosi, Abu stands for Abuku and Acc33 stands for Accession 33, M1 = maturity state 1 (green berries), M2 = maturity state 2 (purple berries). **D** = PGSC number from SpudDB. **E** = Functional classification mainly following the KEGG Pathway Database (++ = if no classification was automatically annotated, the proteins were manually classified) ^[1]^. **F** = The protein score obtained via the MASCOT search algorithm (www.matrixscience.com) against a potato protein database, which was based upon the sequences from *Solanum tuberosum* group Phureja DM1-3 v 6.1, which was completely sequenced by the Potato Genome Consortium 2020. **G** = Calculated PI obtained via the MASCOT search algorithm (www.matrixscience.com) against a potato protein database. **H** = Calculated MW obtained via the MASCOT search algorithm (www.matrixscience.com) against a potato protein database. **I** = Number of peptides matched to the protein through the database search. **J** = Sequence coverage in %. **K** = Unique peptides matched to the sequence. Only proteins with at least two unique peptide were considered true hits. L = Molecular weight (MW) in gel as compared to the theoretically expected MW. M: MW in gel corresponding to the theoretically expected MW ± 15 kDa, S: MW in gel lower than theoretically expected. **L**: MW in gel larger than theoretically expected. **M** = Mean relative spot volume obtained according to three gels of M1 seeds or M2 seeds illustrated by graphs. The first bar (orange) represents the mean normalized spot volume in the gels of M1 seeds of Accession 33. The second bar (light orange) represents the mean normalized spot volume in the gels of the M2 seeds from Accession 33. The third bar (green) stands for the mean normalized spot volume in the gels of the M1 seeds of Abuku 1. The fourth bar (light green) represents the mean normalized spot volume in the gels of the M2 seeds of Abuku 1. The fifth bar (purple) represents the mean normalized spot volume in the gels of the M1 seeds from Olevolosi. The sixth bar (light purple) stands for the mean normalized spot volume in the gels of the M2 seeds of Olevolosi.

| **Spot ID^A^** | **Accession^B^** | **Reg^C^** | **Protein name** | **PGSC numbers** | **KEGG^D^** | **Score^E^** | **PI Calc^F^** | **MW Calc ^G^** | **Pep ^H^** | **SC [%]^I^** | **UPep^J^** | **Size^K^** | **Normalized spot volume^L^** |
| --- | --- | --- | --- | --- | --- | --- | --- | --- | --- | --- | --- | --- | --- |
| **421** | **Olevolosi-M2**  **Acc 33 M1** | **100.34** | **Phosphoglycerate kinase** | **Soltu.DM.07G028580.1** | **Metabolism – Glycolysis ++** | **1162** | **5.4** | **42.3** | **45** | **53.9** | **19** | **L** | **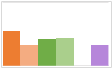** |
|  |  |  | **Hydroxysteroid dehydrogenase** | **Soltu.DM.06G021080.1** | **Biological processes – Growth and seed production ++** | **381** | **7.8** | **42.9** | **9** | **21.6** | **7** | **L** |  |
|  |  |  | **RmlC-like cupins superfamily protein** | **Soltu.DM.09G021450.1** | **Seed storage protein ++** | **90** | **6.1** | **49.9** | **3** | **3.4** | **2** | **L** |  |
| **130** | **Olevolosi-M2**  **Acc 33 M1** | **31.11** | **Phosphoglycerate kinase** | **Soltu.DM.07G028580.1** | **Metabolism – Glycolysis ++** | **839** | **5.4** | **42.3** | **32** | **47.6** | **15** | **L** | **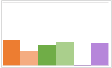** |
|  |  |  | **Hydroxysteroid dehydrogenase** | **Soltu.DM.06G021080.1** | **Biological processes – Growth and seed production ++** | **250** | **7.8** | **42.9** | **7** | **16.1** | **5** | **L** |  |
|  |  |  | **RmlC-like cupins superfamily protein** | **Soltu.DM.09G021500.1** | **Seed storage protein ++** | **74** | **7.0** | **54.4** | **5** | **3.7** | **2** | **L** |  |
| **92** | **Olevolosi-M2** | **14.97** | **Late embryogenesis abundant domain-containing protein / LEA domain-containing protein** | **Soltu.DM.07G020570.1** | **Metabolism ++** | **672** | **5.2** | **56.3** | **35** | **22.4** | **12** | **L** | **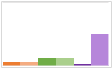** |
|  |  |  | **TCP-1/cpn60 chaperonin family protein** | **Soltu.DM.01G026570.1** | **Genetic information processing - Chaperones and folding catalysts** | **215** | **5.2** | **58.3** | **7** | **15.7** | **7** | **L** |  |
|  |  |  | **2-oxoglutarate (2OG) and Fe(II)-dependent oxygenase superfamily protein** | **Soltu.DM.03G030400.1** | **Metabolism - Flavonoid biosynthesis ++** | **38** | **5.7** | **185.5** | **3** | **0.9** | **2** | **S** |  |
|  |  |  | **hypothetical protein** | **Soltu.DM.02G012540.1** | **Unknown ++** | **34** | **9.2** | **81.2** | **3** | **2.4** | **2** | **L** |  |
| **204** | **Olevolosi-M2**  **Acc 33 M2**  **Abuku M2** | **3.15** | **MLP-like protein** | **Soltu.DM.09G027690.1** | **Environmental Information Processing ++** | **217** | **6.0** | **17.3** | **9** | **14.7** | **3** | **L** | **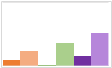** |
|  |  |  | **F-box and associated interaction domains-containing protein** | **Soltu.DM.06G021470.1** | **Genetic information processing ++** | **38** | **5.5** | **22.4** | **2** | **12.4** | **2** | **M** |  |
| **169** | **Olevolosi-M2** | **4.61** | **Late embryogenesis abundant protein (LEA) family protein** | **Soltu.DM.12G002070.1** | **Metabolism** | **369** | **5.4** | **30.0** | **17** | **16.6** | **6** | **M** | **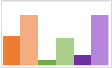** |
|  |  |  | **Glutathione S-transferase family protein** | **Soltu.DM.10G023750.1** | **Metabolism - Metabolism of other amino acids - Glutathione metabolism ++** | **157** | **5.0** | **27.2** | **8** | **23.8** | **5** | **L** |  |
|  |  |  | **Zinc finger (C3HC4-type RING finger) family protein** | **Soltu.DM.02G026990.1** | **Unknown ++** | **38** | **10.5** | **79.4** | **6** | **2.2** | **2** | **S** |  |
| **400** | **Acc33-M2;**  **Abuku M2** | **1.57** | **Cruciferin** | **Soltu.DM.09G026760.1** | **Seed storage protein ++** | **291** | **6.7** | **58.1** | **8** | **7.4** | **3** | **M** | **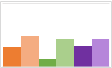** |
|  |  |  | **RmlC-like cupins superfamily protein** | **Soltu.DM.03G000660.1** | **Seed storage protein ++** | **130** | **9.6** | **14.7** | **8** | **13.8** | **3** | **L** |  |
|  |  |  | **RmlC-like cupins superfamily protein** | **Soltu.DM.11G025490.1** | **Seed storage protein ++** | **106** | **5.6** | **57.0** | **3** | **4.5** | **2** | **M** |  |
|  |  |  | **RmlC-like cupins superfamily protein** | **Soltu.DM.09G021500.1** | **Seed storage protein ++** | **99** | **7.0** | **54.4** | **10** | **3.7** | **2** | **M** |  |
|  |  |  | **1-cysteine peroxiredoxin** | **Soltu.DM.03G013100.1** | **Biological processes – ROS detoxification++** | **86** | **6.1** | **24.2** | **3** | **8.2** | **2** | **L** |  |
| **109** | **Olevolosi-M2**  **Abuku M2** | **1.64** | **Alanine-2-oxoglutarate aminotransferase** | **Soltu.DM.01G003360.1** | **Metabolism - Amino acid metabolism - Alanine, aspartate and glutamate metabolism** | **404** | **5.0** | **35.9** | **10** | **20.3** | **6** | **L** | **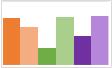** |
|  |  |  | **Alanine-2-oxoglutarate aminotransferase** | **Soltu.DM.05G009660.1** | **Metabolism - Amino acid metabolism - Alanine, aspartate and glutamate metabolism** | **285** | **6.8** | **53.4** | **8** | **11.2** | **6** | **L** |  |
|  |  |  | **Hypothetical protein** | **Soltu.DM.04G033200.1** | **Unknown ++** | **63** | **5.3** | **8.8** | **2** | **8.9** | **2** | **L** |  |
|  |  |  | **P-loop containing nucleoside triphosphate hydrolases superfamily protein** | **Soltu.DM.06G018790.1** | **Genetic information processing - Chromosome and associated proteins ++** | **61** | **5.4** | **49.7** | **3** | **4.4** | **2** | **L** |  |
|  |  |  | **Insulinase (Peptidase family M16) protein** | **Soltu.DM.05G014230.1** | **Metabolism - Peptidases and inhibitors** | **56** | **6.0** | **54.5** | **2** | **4.0** | **2** | **L** |  |
| **294** | **Acc33-M1**  **Abuku M2** | **0.57** | **Alcohol dehydrogenase** | **Soltu.DM.04G025720.1** | **Metabolism - Carbohydrate metabolism - Glycolysis / Gluconeogenesis [EC:1.1.1.1]** | **683** | **6.2** | **41.0** | **43** | **37.2** | **15** | **L** | **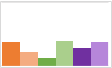** |
|  |  |  | **GroES-like zinc-binding dehydrogenase family protein** | **Soltu.DM.09G018820.1** | **Metabolism - Methane metabolism** | **122** | **6.4** | **40.6** | **4** | **5.3** | **2** | **L** |  |
|  |  |  | **Hydroxysteroid dehydrogenase** | **Soltu.DM.06G021080.1** | **Biological processes – Growth and seed production ++** | **119** | **7.8** | **42.9** | **4** | **9.1** | **3** | **L** |  |
| **183** | **Acc33-M1** | **0.53** | **Mitochondrion-localized small heat shock protein 23.6** | **Soltu.DM.08G024980.1** | **Genetic information processing - Chaperones and folding catalysts** | **252** | **7.3** | **23.9** | **22** | **30.8** | **7** | **L** | **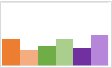** |
| **164** | **Acc33-M1**  **Abuku M2** | **0.23** | **Glutathione S-transferase, C-terminal-like;Translation elongation  factor EF1B/ribosomal protein S6** | **Soltu.DM.01G037330.1** | **Environmental information processing – biological defense response ++** | **268** | **4.3** | **25.3** | **12** | **29.7** | **7** | **L** | **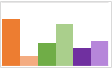** |
|  |  |  | **Translation elongation factor EF1B/ribosomal protein S6 family protein** | **Soltu.DM.11G025220.1** | **Genetic Information Processing - Translation - RNA transport and biogenesis ++** | **140** | **4.4** | **24.5** | **6** | **13.2** | **3** | **L** |  |
| **512** | **Olevolosi-M2** | **3.56** | **Glycosyl hydrolase family protein** | **Soltu.DM.06G029150.1** | **Metabolism - Hydrolases –Glycosylases- [EC 3.2.1.21] ++** | **439** | **5.7** | **55.9** | **20** | **14.3** | **8** | **L** | **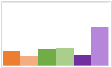** |
|  |  |  | **Glycosyl hydrolase family protein** | **Soltu.DM.06G029150.2** | **Metabolism - Hydrolases –Glycosylases- [EC 3.2.1.21] ++** | **392** | **6.9** | **66.0** | **20** | **10.9** | **7** | **L** |  |
|  |  |  | **Glycosyl hydrolase family protein** | **Soltu.DM.06G029160.1** | **Metabolism - Hydrolases –Glycosylases- [EC 3.2.1.21] ++** | **286** | **8.5** | **68.7** | **14** | **8.0** | **5** | **L** |  |
|  |  |  | **Glycosyl hydrolase family protein** | **Soltu.DM.11G024490.2** | **Metabolism - Hydrolases –Glycosylases- [EC 3.2.1.21] ++** | **121** | **9.0** | **69.6** | **7** | **4.4** | **3** | **L** |  |
|  |  |  | **GTP binding Elongation factor Tu family protein** | **Soltu.DM.06G005580.1** | **Genetic Information Processing - Translation - RNA transport and biogenesis** | **107** | **9.8** | **49.2** | **3** | **3.8** | **2** | **L** |  |
| **510** | **Olevolosi-M2**  **Acc 33 M1** | **4.75** | **Glycosyl hydrolase family protein** | **Soltu.DM.06G029150.1** | **Metabolism - Hydrolases –Glycosylases- [EC 3.2.1.21] ++** | **276** | **5.7** | **55.9** | **11** | **10.9** | **5** | **L** | **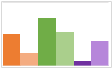** |
|  |  |  | **Glycosyl hydrolase family protein** | **Soltu.DM.06G029150.2** | **Metabolism - Hydrolases –Glycosylases- [EC 3.2.1.21] ++** | **268** | **6.9** | **66.0** | **14** | **9.2** | **5** | **L** |  |
|  |  |  | **GTP binding Elongation factor Tu family protein** | **Soltu.DM.06G005560.1** | **Genetic Information Processing - Translation - RNA transport and biogenesis** | **184** | **9.4** | **35.7** | **6** | **12.0** | **4** | **L** |  |
|  |  |  | **Glycosyl hydrolase family protein** | **Soltu.DM.06G029160.1** | **Metabolism - Hydrolases –Glycosylases- [EC 3.2.1.21] ++** | **173** | **8.5** | **68.7** | **9** | **5.9** | **3** | **L** |  |
|  |  |  | **RmlC-like cupins superfamily protein** | **Soltu.DM.09G021500.1** | **Seed storage protein ++** | **89** | **7.0** | **54.4** | **4** | **6.2** | **2** | **L** |  |
| **131** | **Olevolosi-M2**  **Acc 33 M1** | **3.37** | **Actin** | **Soltu.DM.11G008990.1** | **Signaling and cellular processes - Cytoskeleton proteins** | **683** | **5.2** | **41.8** | **30** | **31.3** | **11** | **L** | **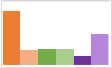** |
|  |  |  | **Actin** | **Soltu.DM.03G011750.1** | **Signaling and cellular processes - Cytoskeleton proteins** | **661** | **5.2** | **41.7** | **28** | **31.3** | **11** | **L** |  |
|  |  |  | **Actin-11** | **Soltu.DM.04G007480.1** | **Signaling and cellular processes - Cytoskeleton proteins** | **488** | **5.2** | **41.6** | **23** | **23.6** | **9** | **L** |  |
|  |  |  | **Phosphoglycerate kinase** | **Soltu.DM.07G028580.1** | **Metabolism – Glycolysis ++** | **156** | **5.4** | **42.3** | **2** | **6.7** | **2** | **L** |  |
|  |  |  | **Hydroxysteroid dehydrogenase** | **Soltu.DM.06G021080.1** | **Biological processes – Growth and seed production ++** | **95** | **7.8** | **42.9** | **4** | **5.7** | **2** | **L** |  |
|  |  |  | **RmlC-like cupins superfamily protein** | **Soltu.DM.09G021500.1** | **Seed storage protein ++** | **94** | **7.0** | **54.4** | **4** | **5.0** | **3** | **L** |  |
| **541** | **Olevolosi-M1** | **0.17** | **Voltage dependent anion channel** | **Soltu.DM.03G008530.1** | **Protein families: genetic information processing - Mitochondrial biogenesis - Protein families: signalling and cellular processes - Ion channels** | **51** | **8.8** | **29.4** | **2** | **6.2** | **2** | **L** | **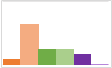** |
| **157** | **Olevolosi-M2**  **Abuku M1** | **2.52** | **Voltage dependent anion channel** | **Soltu.DM.03G008530.1** | **Protein families: genetic information processing - Mitochondrial biogenesis - Protein families: signaling and cellular processes - Ion channels** | **366** | **8.8** | **29.4** | **18** | **24.6** | **9** | **L** | **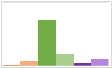** |
|  |  |  | **Voltage dependent anion channel** | **Soltu.DM.01G009390.1** | **Protein families: genetic information processing - Mitochondrial biogenesis - Protein families: signaling and cellular processes - Ion channels** | **241** | **8.7** | **29.4** | **9** | **13.8** | **6** | **L** |  |
| **454** | **Olevolosi-M1**  **Abuku M1** | **0.57** | **Glycosyl hydrolase family protein** | **Soltu.DM.06G029150.1** | **Metabolism - Hydrolases –Glycosylases- [EC 3.2.1.21] ++** | **539** | **5.7** | **55.9** | **20** | **16.2** | **9** | **L** | **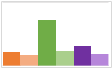** |
|  |  |  | **Glycosyl hydrolase family protein** | **Soltu.DM.06G029150.2** | **Metabolism - Hydrolases –Glycosylases- [EC 3.2.1.21] ++** | **478** | **6.9** | **66.0** | **22** | **13.5** | **8** | **L** |  |
|  |  |  | **Glycosyl hydrolase family protein** | **Soltu.DM.06G029160.1** | **Metabolism - Hydrolases –Glycosylases- [EC 3.2.1.21] ++** | **430** | **8.5** | **68.7** | **17** | **12.1** | **8** | **L** |  |
| **95** | **Olevolosi-M2**  **Abuku M1** | **2.32** | **RmlC-like cupins superfamily protein** | **Soltu.DM.09G021500.1** | **Seed storage protein ++** | **155** | **7.0** | **54.4** | **12** | **7.3** | **4** | **L** | **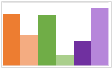** |
|  |  |  | **RmlC-like cupins superfamily protein** | **Soltu.DM.03G000660.1** | **Seed storage protein ++** | **125** | **9.6** | **14.7** | **4** | **13.8** | **3** | **L** |  |
|  |  |  | **Glutamate decarboxylase** | **Soltu.DM.03G019160.3** | **Metabolism - Carbohydrate metabolism - Amino acid metabolism** | **117** | **5.7** | **56.7** | **5** | **6.0** | **3** | **L** |  |
|  |  |  | **Cruciferin** | **Soltu.DM.09G026760.1** | **Seed storage protein ++** | **94** | **6.7** | **58.1** | **4** | **3.9** | **2** | **L** |  |
|  |  |  | **Cupin family protein** | **Soltu.DM.09G024720.1** | **Seed storage protein ++** | **77** | **8.6** | **65.7** | **3** | **3.5** | **2** | **L** |  |
|  |  |  | **DNA binding** | **Soltu.DM.11G004880.1** | **Unknown ++** | **63** | **4.7** | **56.6** | **4** | **1.7** | **2** | **L** |  |
|  |  |  | **RmlC-like cupins superfamily protein** | **Soltu.DM.09G021460.1** | **Seed storage protein ++** | **53** | **7.9** | **53.4** | **3** | **4.0** | **2** | **L** |  |
|  |  |  | **Structural maintenance of chromosomes (SMC) family protein** | **Soltu.DM.06G034250.1** | **Genetic information processing - Chromosome and associated proteins** | **45** | **9.1** | **141.0** | **3** | **1.3** | **2** | **S** |  |
| **121** | **Acc33-M1**  **Abuku M1** | **0.32** | **Eukaryotic translation initiation factor 4A1** | **Soltu.DM.12G004380.1** | **Genetic Information Processing – Translation - RNA transport and biogenesis** | **103** | **5.4** | **46.8** | **4** | **7.5** | **3** | **L** | **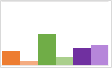** |
| **84** | **Olevolosi-M1** | **0.29** | **Phosphoglycerate mutase, 2,3-bisphosphoglycerate-independent** | **Soltu.DM.07G014610.1** | **Metabolism - Carbohydrate metabolism - Glycolysis / Gluconeogenesis ++** | **176** | **5.3** | **61.2** | **6** | **7.5** | **5** | **L** | **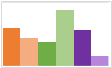** |
|  |  |  | **Heat-shock protein 70T-2** | **Soltu.DM.09G024280.1** | **Genetic information processing - Chaperones and folding catalysts** | **150** | **5.5** | **62.4** | **6** | **8.6** | **4** | **L** |  |
|  |  |  | **RmlC-like cupins superfamily protein** | **Soltu.DM.09G021460.1** | **Seed storage protein ++** | **100** | **7.9** | **53.4** | **3** | **4.6** | **2** | **L** |  |
|  |  |  | **RmlC-like cupins superfamily protein** | **Soltu.DM.09G021450.1** | **Seed storage protein ++** | **85** | **6.1** | **49.9** | **3** | **3.4** | **2** | **L** |  |
|  |  |  | **Thiamine pyrophosphate dependent pyruvate decarboxylase family protein** | **Soltu.DM.10G019450.1** | **Metabolism - Carbohydrate metabolism - Glycolysis / Gluconeogenesis** | **82** | **5.7** | **65.5** | **2** | **3.0** | **2** | **L** |  |
| **60** | **Olevolosi-M1**  **Acc 33 M1** | **0.49** | **Glycosyl hydrolase family protein** | **Soltu.DM.06G029150.1** | **Metabolism - Hydrolases –Glycosylases- [EC 3.2.1.21] ++** | **224** | **5.7** | **55.9** | **8** | **11.7** | **5** | **L** | **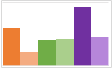** |
|  |  |  | **Glycosyl hydrolase family protein** | **Soltu.DM.06G029150.2** | **Metabolism - Hydrolases –Glycosylases- [EC 3.2.1.21] ++** | **220** | **6.9** | **66.0** | **10** | **9.8** | **5** | **L** |  |
|  |  |  | **Glycosyl hydrolase family protein** | **Soltu.DM.06G029160.1** | **Metabolism - Hydrolases –Glycosylases- [EC 3.2.1.21] ++** | **157** | **8.5** | **68.7** | **5** | **6.5** | **3** | **L** |  |
| **10** | **Acc33-M1**  **Abuku M1** | **0.22** | **Heat shock protein** | **Soltu.DM.03G029350.1** | **Genetic information processing - Chaperones and folding catalysts** | **1503** | **5.8** | **101.1** | **56** | **34.0** | **29** | **L** | **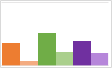** |
|  |  |  | **Casein lytic proteinase B3** | **Soltu.DM.02G031120.1** | **Genetic information processing - Chaperones and folding catalysts ++** | **184** | **6.1** | **110.3** | **11** | **4.4** | **4** | **L** |  |
|  |  |  | **RmlC-like cupins superfamily protein ++** | **Soltu.DM.09G021500.1** | **Seed storage protein** | **143** | **7.0** | **54.4** | **9** | **5.0** | **3** | **L** |  |
| **463** | **Olevolosi-M1**  **Acc 33 M1**  **Abuku M1** | **0.29** | **Glycosyl hydrolase family protein** | **Soltu.DM.06G029150.1** | **Metabolism - Hydrolases –Glycosylases- [EC 3.2.1.21] ++** | **106** | **5.7** | **55.9** | **4** | **4.3** | **2** | **L** | **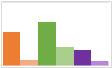** |
|  |  |  | **Glycosyl hydrolase family protein** | **Soltu.DM.06G029150.2** | **Metabolism - Hydrolases –Glycosylases- [EC 3.2.1.21] ++** | **66** | **6.9** | **66.0** | **3** | **3.5** | **2** | **L** |  |
| **446** | **Acc33-M1**  **Abuku M2** | **0.20** | **Argonaute family protein** | **Soltu.DM.01G005850.1** | **Genetic information processing - Messenger RNA biogenesis** | **1306** | **9.7** | **101.7** | **54** | **26.4** | **27** | **L** | **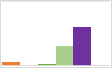** |
|  |  |  | **Argonaute family protein** | **Soltu.DM.06G028860.1** | **Genetic information processing - Messenger RNA biogenesis** | **1087** | **9.7** | **101.9** | **46** | **23.2** | **22** | **L** |  |
|  |  |  | **Argonaute family protein** | **Soltu.DM.01G035930.1** | **Genetic information processing - Messenger RNA biogenesis** | **203** | **9.9** | **90.5** | **7** | **4.8** | **4** | **L** |  |
| **360** | **Acc33-M2**  **Abuku M1** | **13.55** | **Cruciferin** | **Soltu.DM.09G026760.1** | **Seed storage protein ++** | **358** | **6.7** | **58.1** | **17** | **11.5** | **6** | **M** | **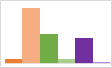** |
|  |  |  | **NAD(P)-linked oxidoreductase superfamily protein** | **Soltu.DM.09G009380.1** | **Metabolism - Glycolysis - Gluconeogenesis** | **337** | **6.1** | **58.1** | **14** | **20.4** | **7** | **M** |  |
|  |  |  | **Annexin** | **Soltu.DM.04G029320.1** | **Environmental Information Processing ++** | **262** | **5.3** | **36.3** | **9** | **12.7** | **5** | **L** |  |
|  |  |  | **NAD(P)-binding Rossmann-fold superfamily protein** | **Soltu.DM.01G038420.1** | **Genetic information processing - Ubiquitin system ++** | **160** | **9.4** | **35.8** | **3** | **10.6** | **3** | **L** |  |
|  |  |  | **Hydroxysteroid dehydrogenase** | **Soltu.DM.06G028000.1** | **Biological processes – Growth and seed production ++** | **154** | **5.6** | **37.3** | **7** | **12.0** | **5** | **L** |  |
|  |  |  | **RmlC-like cupins superfamily protein** | **Soltu.DM.03G000660.1** | **Seed storage protein ++** | **144** | **9.6** | **38.7** | **7** | **13.8** | **3** | **L** |  |
|  |  |  | **RmlC-like cupins superfamily protein** | **Soltu.DM.09G021500.1** | **Seed storage protein ++** | **105** | **7.0** | **14.7** | **8** | **3.7** | **2** | **L** |  |
|  |  |  | **P-loop containing nucleoside triphosphate hydrolases superfamily protein** | **Soltu.DM.03G014080.1** | **Genetic Information Processing – Translation - Ribosome biogenesis in eukaryotes** | **38** | **9.1** | **54.4** | **2** | **1.2** | **2** | **L** |  |
| **394** | **Olevolosi-M1** | **0.04** | **RmlC-like cupins superfamily protein** | **Soltu.DM.11G025490.1** | **Seed storage protein ++** | **166** | **5.6** | **57.0** | **8** | **6.9** | **3** | **M** | **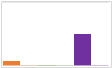** |
|  |  |  | **Triosephosphate isomerase** | **Soltu.DM.04G007490.1** | **Metabolism - Carbohydrate metabolism - Glycolysis / Gluconeogenesis ++** | **103** | **5.7** | **27.0** | **2** | **8.7** | **2** | **L** |  |
|  |  |  | **20S proteasome alpha subunit G1** | **Soltu.DM.10G026260.1** | **Genetic Information Processing - Folding, sorting and degradation - Proteasome** | **73** | **6.1** | **27.1** | **3** | **7.6** | **2** | **L** |  |
| **562** | **Acc33-M1**  **Abuku M1** | **0.15** | **Oleosin family protein** | **Soltu.DM.12G028510.1** | **Oleosome - oil body protein ++** | **118** | **10.1** | **17.5** | **4** | **12.7** | **2** | **L** | **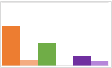** |
| **449** | **Acc33-M1**  **Abuku M1** | **0.03** | **Argonaute family protein** | **Soltu.DM.01G005850.1** | **Genetic information processing - Messenger RNA biogenesis** | **1095** | **9.7** | **101.7** | **36** | **24.4** | **24** | **L** | **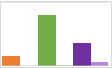** |
|  |  |  | **Argonaute family protein** | **Soltu.DM.06G028860.1** | **Genetic information processing - Messenger RNA biogenesis** | **587** | **9.7** | **101.9** | **22** | **14.1** | **14** | **L** |  |

Reference

1. Kanehisa, M., Furumichi, M., Sato, Y., Kawashima, M. & Ishiguro-Watanabe, M. KEGG for taxonomy-based analysis of pathways and genomes. *Nucleic Acids Res.* **51**, D587–D592 (2023).
