# Supplementary material for: Effect of berry maturity stages on the germination and protein constituents of African nightshade (Solanum scabrum) seeds
Source: Sci Rep. 2024 Dec 16;14:30482. doi: 10.1038/s41598-024-80312-6 (PMC11649806; doi:10.1038/s41598-024-80312-6)
Supplement: Supplementary file 6 — Supplementary Material 6 [file 41598_2024_80312_MOESM6_ESM.docx]

**Supplementary table S3: Identification of proteins from spots with changed abundance in comparison 1 between M2 and M1 of Acc 33**. A Student's t-test was performed (p-value ≤ 0.05) to determine significant changes in spot volume based on normalised relative spot volume. Only alterations of at least 1.5-fold in spot volume were considered to represent true alterations in protein level. Analysis was performed with Delta2D by DECODON. **A** = SpotID as defined by the Delta2D software from DECODON on the master gel from the 2D PAGE gels. Corresponding spots of all gels are labelled with the same ID. **B** = Accession for which the proteins in the row were identified. **C** = Proteins were identified from spots picked from the following 2D PAGE gel. Ole stands for Olevolosi, Abu stands for Abuku and Acc33 stands for Accession 33, M1 = maturity state 1 (green berries), M2 = maturity state 2 (purple berries). **D** = Regulation of a spot according to the comparison between groups. Regulation is given as the ratio between spot abundance (M2 / M1). **E** = Functional classification mainly following the KEGG Pathway Database (++ = if no classification was automatically annotated, the proteins were manually classified) ^[1]^.. **F** = The protein score obtained via the MASCOT search algorithm (www.matrixscience.com) against a potato protein database, which was based upon the sequences from *Solanum tuberosum* group Phureja DM1-3 v 6.1, which was completely sequenced by the Potato Genome Consortium 2020. **G** = Calculated PI obtained via the MASCOT search algorithm (www.matrixscience.com) against a potato protein database. **H** = Calculated MW obtained via the MASCOT search algorithm (www.matrixscience.com) against a potato protein database. **I** = Number of peptides matched to the protein through the database search. **J** = Sequence coverage in %. **K** = Unique peptides matched to the sequence. Only proteins with at least two unique peptide were considered true hits. L = Molecular weight (MW) in gel as compared to the theoretically expected MW. M: MW in gel corresponding to the theoretically expected MW ± 15 kDa, S: MW in gel lower than theoretically expected. **L**: MW in gel larger than theoretically expected. **M** = Mean relative spot volume obtained according to three gels of M1 seeds or M2 seeds illustrated by graphs. The first bar (orange) represents the mean normalized spot volume in the gels of M1 seeds of Accession 33. The second bar (light orange) represents the mean normalized spot volume in the gels of the M2 seeds from Accession 33. The third bar (green) stands for the mean normalized spot volume in the gels of the M1 seeds of Abuku 1. The fourth bar (light green) represents the mean normalized spot volume in the gels of the M2 seeds of Abuku 1. The fifth bar (purple) represents the mean normalized spot volume in the gels of the M1 seeds from Olevolosi. The sixth bar (light purple) stands for the mean normalized spot volume in the gels of the M2 seeds of Olevolosi.

| **Spot ID^A^** | **Accession^B^** | **Identified from Gel^C^** | **Reg^D^** | **Protein name** | **PGSC numbers** | **KEGG^E^** | **Score^F^** | **PI Calc^G^** | **MW Calc ^H^** | **Pep ^I^** | **SC [%]^J^** | **UPep^K^** | **Size^L^** | **Normalized spot volume^M^** |
| --- | --- | --- | --- | --- | --- | --- | --- | --- | --- | --- | --- | --- | --- | --- |
| **10** | **Abuku 1-M1** | **Acc 33 - M1** | **0.42** | **Heat shock protein** | **Soltu.DM.03G029350.1** | **Genetic information processing - Chaperones and folding catalysts** | **1503** | **5.8** | **101.1** | **56** | **34.0** | **29** | **L** |  |
| **10** | **Abuku 1-M1** | **Acc 33 - M1** | **0.42** | **Casein lytic proteinase B3** | **Soltu.DM.02G031120.1** | **Genetic information processing - Chaperones and folding catalysts ++** | **184** | **6.1** | **110.3** | **11** | **4.4** | **4** | **L** |  |
| **10** | **Abuku 1-M1** | **Acc 33 - M1** | **0.42** | **RmlC-like cupins superfamily protein** | **Soltu.DM.09G021500.1** | **Seed storage protein ++** | **143** | **7.0** | **54.4** | **9** | **5.0** | **3** | **L** |  |
| **121** | **Abuku 1-M1** | **Acc 33 - M1** | **0.28** | **Eukaryotic translation initiation factor 4A1** | **Soltu.DM.12G004380.1** | **Genetic Information Processing – Translation - RNA transport and biogenesis** | **103** | **5.4** | **46.8** | **4** | **7.5** | **3** | **L** |  |
| **449** | **Abuku 1-M1** | **Acc 33 - M1** | **0.02** | **Argonaute family protein** | **Soltu.DM.01G005850.1** | **Genetic information processing - Messenger RNA biogenesis** | **1095** | **9.7** | **101.7** | **36** | **24.4** | **24** | **L** |  |
| **449** | **Abuku 1-M1** | **Acc 33 - M1** | **0.02** | **Argonaute family protein** | **Soltu.DM.06G028860.1** | **Genetic information processing - Messenger RNA biogenesis** | **587** | **9.7** | **101.9** | **22** | **14.1** | **14** | **L** |  |
| **553** | **Abuku 1-M1** | **Acc 33 - M1** | **0.18** | **MLP-like protein** | **Soltu.DM.09G027690.1** | **Environmental Information Processing ++** | **221** | **6.0** | **17.3** | **13** | **23.1** | **5** | **M** |  |
| **553** | **Abuku 1-M1** | **Acc 33 - M1** | **0.18** | **MLP-like protein** | **Soltu.DM.09G027700.1** | **Environmental Information Processing ++** | **134** | **5.2** | **17.3** | **6** | **18.8** | **3** | **L** |  |
| **562** | **Abuku 1-M1** | **Acc 33 - M1** | **0.01** | **Oleosin family protein** | **Soltu.DM.12G028510.1** | **Oleosome - oil body protein ++** | **118** | **10.1** | **17.5** | **4** | **12.7** | **2** | **L** |  |
| **101** | **Abuku 1-M1** | **Acc 33 – M2** | **0.21** | **ATP synthase alpha/beta family protein** | **Soltu.DM.02G026440.1** | **Metabolism - Energy metabolism - Oxidative phosphorylation** | **60** | **6.0** | **59.5** | **2** | **3.8** | **2** | **L** |  |
| **101** | **Abuku 1-M1** | **Acc 33 – M2** | **0.21** | **Xylose isomerase family protein** | **Soltu.DM.07G001920.1** | **Metabolism – Fructose and mannose metabolism** | **54** | **9.2** | **112.8** | **2** | **1.6** | **2** | **M** |  |
| **360** | **Abuku 1-M1** | **Acc 33 – M2** | **0.14** | **Cruciferin** | **Soltu.DM.09G026760.1** | **Seed storage protein ++** | **358** | **6.7** | **58.1** | **17** | **11.5** | **6** | **M** |  |
| **360** | **Abuku 1-M1** | **Acc 33 – M2** | **0.14** | **NAD(P)-linked oxidoreductase superfamily protein** | **Soltu.DM.09G009380.1** | **Metabolism - Glycolysis - Gluconeogenesis** | **337** | **6.1** | **58.1** | **14** | **20.4** | **7** | **M** |  |
| **360** | **Abuku 1-M1** | **Acc 33 – M2** | **0.14** | **Annexin** | **Soltu.DM.04G029320.1** | **Environmental Information Processing ++** | **262** | **5.3** | **36.3** | **9** | **12.7** | **5** | **L** |  |
| **360** | **Abuku 1-M1** | **Acc 33 – M2** | **0.14** | **NAD(P)-binding Rossmann-fold superfamily protein** | **Soltu.DM.01G038420.1** | **Genetic information processing - Ubiquitin system ++** | **160** | **9.4** | **35.8** | **3** | **10.6** | **3** | **L** |  |
| **360** | **Abuku 1-M1** | **Acc 33 – M2** | **0.14** | **Hydroxysteroid dehydrogenase** | **Soltu.DM.06G028000.1** | **Biological processes – Growth and seed production ++** | **154** | **5.6** | **37.3** | **7** | **12.0** | **5** | **L** |  |
| **360** | **Abuku 1-M1** | **Acc 33 – M2** | **0.14** | **RmlC-like cupins superfamily protein** | **Soltu.DM.03G000660.1** | **Seed storage protein ++** | **144** | **9.6** | **38.7** | **7** | **13.8** | **3** | **L** |  |
| **360** | **Abuku 1-M1** | **Acc 33 – M2** | **0.14** | **RmlC-like cupins superfamily protein** | **Soltu.DM.09G021500.1** | **Seed storage protein ++** | **105** | **7.0** | **14.7** | **8** | **3.7** | **2** | **L** |  |
| **360** | **Abuku 1-M1** | **Acc 33 – M2** | **0.14** | **P-loop containing nucleoside triphosphate hydrolases superfamily protein** | **Soltu.DM.03G014080.1** | **Genetic Information Processing – Translation - Ribosome biogenesis in eukaryotes** | **38** | **9.1** | **54.4** | **2** | **1.2** | **2** | **L** |  |
| **475** | **Abuku 1-M1** | **Acc 33 – M2** | **0.42** | **Cupin family protein** | **Soltu.DM.09G024720.1** | **Seed storage protein ++** | **264** | **8.6** | **65.7** | **14** | **8.6** | **6** | **L** |  |
| **475** | **Abuku 1-M1** | **Acc 33 – M2** | **0.42** | **Cupin family protein** | **Soltu.DM.09G024710.1** | **Seed storage protein ++** | **121** | **5.9** | **42.5** | **5** | **5.6** | **2** | **L** |  |
| **157** | **Abuku 1-M1** | **Olevolosi – M2** | **0.26** | **Voltage dependent anion channel** | **Soltu.DM.03G008530.1** | **Protein families: genetic information processing - Mitochondrial biogenesis - Protein families: signalling and cellular processes - Ion channels** | **366** | **8.8** | **29.4** | **18** | **24.6** | **9** | **L** |  |
| **157** | **Abuku 1-M1** | **Olevolosi – M2** | **0.26** | **Voltage dependent anion channel** | **Soltu.DM.01G009390.1** | **Protein families: genetic information processing - Mitochondrial biogenesis - Protein families: signalling and cellular processes - Ion channels** | **241** | **8.7** | **29.4** | **9** | **13.8** | **6** | **L** |  |
| **382** | **Abuku 1-M1** | **Olevolosi – M2** | **0.52** | **RmlC-like cupins superfamily protein** | **Soltu.DM.09G021460.1** | **Seed storage protein ++** | **333** | **7.9** | **53.4** | **23** | **12.4** | **4** | **M** |  |
| **382** | **Abuku 1-M1** | **Olevolosi – M2** | **0.52** | **RmlC-like cupins superfamily protein** | **Soltu.DM.09G021500.1** | **Seed storage protein ++** | **215** | **7.0** | **54.4** | **12** | **10.8** | **4** | **M** |  |
| **454** | **Abuku 1-M1** | **Olevolosi – M1** | **0.32** | **Glycosyl hydrolase family protein** | **Soltu.DM.06G029150.1** | **Metabolism - Hydrolases –Glycosylases- [EC 3.2.1.21] ++** | **539** | **5.7** | **55.9** | **20** | **16.2** | **9** | **L** |  |
| **454** | **Abuku 1-M1** | **Olevolosi – M1** | **0.32** | **Glycosyl hydrolase family protein** | **Soltu.DM.06G029150.2** | **Metabolism - Hydrolases –Glycosylases- [EC 3.2.1.21] ++** | **478** | **6.9** | **66.0** | **22** | **13.5** | **8** | **L** |  |
| **454** | **Abuku 1-M1** | **Olevolosi – M1** | **0.32** | **Glycosyl hydrolase family protein** | **Soltu.DM.06G029160.1** | **Metabolism - Hydrolases –Glycosylases- [EC 3.2.1.21] ++** | **430** | **8.5** | **68.7** | **17** | **12.1** | **8** | **L** |  |
| **491** | **Abuku 1-M1** | **Olevolosi – M2** | **0.28** | **Cupin family protein** | **Soltu.DM.09G024720.1** | **Seed storage protein ++** | **207** | **8.6** | **65.7** | **20** | **7.6** | **6** | **M** |  |
| **491** | **Abuku 1-M1** | **Olevolosi – M2** | **0.28** | **Cupin family protein** | **Soltu.DM.09G024710.1** | **Seed storage protein ++** | **136** | **5.9** | **42.5** | **6** | **5.6** | **2** | **L** |  |
| **95** | **Abuku 1-M1** | **Olevolosi – M2** | **0.23** | **RmlC-like cupins superfamily protein** | **Soltu.DM.09G021500.1** | **Seed storage protein ++** | **155** | **7.0** | **54.4** | **12** | **7.3** | **4** | **L** |  |
| **95** | **Abuku 1-M1** | **Olevolosi – M2** | **0.23** | **RmlC-like cupins superfamily protein** | **Soltu.DM.03G000660.1** | **Seed storage protein ++** | **125** | **9.6** | **14.7** | **4** | **13.8** | **3** | **L** |  |
| **95** | **Abuku 1-M1** | **Olevolosi – M2** | **0.23** | **Glutamate decarboxylase** | **Soltu.DM.03G019160.3** | **Metabolism - Carbohydrate metabolism - Amino acid metabolism** | **117** | **5.7** | **56.7** | **5** | **6.0** | **3** | **L** |  |
| **95** | **Abuku 1-M1** | **Olevolosi – M2** | **0.23** | **Cruciferin** | **Soltu.DM.09G026760.1** | **Seed storage protein ++** | **94** | **6.7** | **58.1** | **4** | **3.9** | **2** | **L** |  |
| **95** | **Abuku 1-M1** | **Olevolosi – M2** | **0.23** | **Cupin family protein** | **Soltu.DM.09G024720.1** | **Seed storage protein ++** | **77** | **8.6** | **65.7** | **3** | **3.5** | **2** | **L** |  |
| **95** | **Abuku 1-M1** | **Olevolosi – M2** | **0.23** | **DNA binding** | **Soltu.DM.11G004880.1** | **Unknown ++** | **63** | **4.7** | **56.6** | **4** | **1.7** | **2** | **L** |  |
| **95** | **Abuku 1-M1** | **Olevolosi – M2** | **0.23** | **RmlC-like cupins superfamily protein** | **Soltu.DM.09G021460.1** | **Seed storage protein ++** | **53** | **7.9** | **53.4** | **3** | **4.0** | **2** | **L** |  |
| **95** | **Abuku 1-M1** | **Olevolosi – M2** | **0.23** | **Structural maintenance of chromosomes (SMC) family protein** | **Soltu.DM.06G034250.1** | **Genetic information processing - Chromosome and associated proteins** | **45** | **9.1** | **141.0** | **3** | **1.3** | **2** | **S** |  |
| **365** | **Abuku 1-M1** | **Olevolosi – M1** | **0.49** | **RmlC-like cupins superfamily protein** | **Soltu.DM.09G021500.1** | **Seed storage protein ++** | **375** | **7.0** | **54.4** | **27** | **18.3** | **6** | **M** |  |
| **463** | **Abuku 1-M1** | **Olevolosi – M1** | **0.43** | **Glycosyl hydrolase family protein** | **Soltu.DM.06G029150.1** | **Metabolism - Hydrolases –Glycosylases- [EC 3.2.1.21] ++** | **106** | **5.7** | **55.9** | **4** | **4.3** | **2** | **L** |  |
| **463** | **Abuku 1-M1** | **Olevolosi – M1** | **0.43** | **Glycosyl hydrolase family protein** | **Soltu.DM.06G029150.2** | **Metabolism - Hydrolases –Glycosylases- [EC 3.2.1.21] ++** | **66** | **6.9** | **66.0** | **3** | **3.5** | **2** | **L** |  |
| **411** | **Abuku 1-M1** | **Olevolosi – M1** | **0.02** | **Cruciferin** | **Soltu.DM.09G026760.1** | **Seed storage protein ++** | **168** | **6.7** | **58.1** | **9** | **6.4** | **3** | **L** |  |
| **100** | **Abuku 1-M2** | **Acc 33 – M1** | **5.89** | **ATP synthase alpha/beta family protein** | **Soltu.DM.05G004470.1** | **Metabolism - Energy metabolism** | **1633** | **5.6** | **59.5** | **74** | **51.3** | **23** | **L** |  |
| **100** | **Abuku 1-M2** | **Acc 33 – M1** | **5.89** | **ATP synthase alpha/beta family protein** | **Soltu.DM.04G002460.1** | **Metabolism - Energy metabolism - Oxidative phosphorylation** | **1486** | **5.7** | **59.8** | **66** | **47.3** | **21** | **L** |  |
| **100** | **Abuku 1-M2** | **Acc 33 – M1** | **5.89** | **ATPase, V1 complex, subunit B protein** | **Soltu.DM.01G051310.1** | **Metabolism - Energy metabolism - Oxidative phosphorylation** | **81** | **4.9** | **54.2** | **3** | **5.7** | **3** | **L** |  |
| **113** | **Abuku 1-M2** | **Olevolosi – M2** | **7.93** | **embryonic cell protein** | **Soltu.DM.09G004550.1** | **Unknown ++** | **520** | **5.3** | **43.1** | **25** | **18.8** | **7** | **L** |  |
| **113** | **Abuku 1-M2** | **Olevolosi – M2** | **7.93** | **Enolase** | **Soltu.DM.03G028540.1** | **Metabolism - Carbohydrate metabolism - Glycolysis / Gluconeogenesis ++** | **138** | **6.1** | **52.1** | **4** | **6.4** | **3** | **L** |  |
| **178** | **Abuku 1-M2** | **Olevolosi – M2** | **1.93** | **RmlC-like cupins superfamily protein** | **Soltu.DM.09G021500.1** | **Seed storage protein ++** | **101** | **7.0** | **54.4** | **8** | **3.7** | **2** | **M** |  |
| **178** | **Abuku 1-M2** | **Olevolosi – M2** | **1.93** | **Cupin family protein** | **Soltu.DM.09G024720.1** | **Seed storage protein ++** | **65** | **8.6** | **65.7** | **3** | **4.1** | **2** | **S** |  |
| **178** | **Abuku 1-M2** | **Olevolosi – M2** | **1.93** | **RmlC-like cupins superfamily protein** | **Soltu.DM.03G000660.1** | **Seed storage protein ++** | **43** | **9.6** | **14.7** | **3** | **12.2** | **2** | **L** |  |
| **194** | **Abuku 1-M2** | **Acc 33 – M1** | **2.83** | **Cruciferin** | **Soltu.DM.03G000650.1** | **Storage protein ++** | **214** | **7.9** | **42.6** | **6** | **13.4** | **5** | **M** |  |
| **194** | **Abuku 1-M2** | **Acc 33 – M1** | **2.83** | **RmlC-like cupins superfamily protein** | **Soltu.DM.09G021500.1** | **Seed storage protein ++** | **142** | **7.0** | **54.4** | **8** | **6.2** | **3** | **M** |  |
| **194** | **Abuku 1-M2** | **Acc 33 – M1** | **2.83** | **RmlC-like cupins superfamily protein** | **Soltu.DM.09G021460.1** | **Seed storage protein ++** | **106** | **7.9** | **53.4** | **4** | **4.0** | **2** | **M** |  |
| **196** | **Abuku 1-M2** | **Olevolosi – M2** | **1.86** | **HSP20-like chaperones superfamily protein** | **Soltu.DM.06G031840.1** | **Genetic Information Processing - Folding, sorting and degradation** | **272** | **5.8** | **17.6** | **9** | **33.1** | **6** | **L** |  |
| **196** | **Abuku 1-M2** | **Olevolosi – M2** | **1.86** | **HSP20-like chaperones superfamily protein** | **Soltu.DM.06G031870.1** | **Genetic Information Processing - Folding, sorting and degradation** | **238** | **6.2** | **17.6** | **9** | **34.4** | **6** | **L** |  |
| **196** | **Abuku 1-M2** | **Olevolosi – M2** | **1.86** | **Heat shock protein 17.4** | **Soltu.DM.09G009430.1** | **Genetic Information Processing -Folding, sorting and degradation** | **133** | **5.3** | **17.9** | **3** | **16.6** | **3** | **L** |  |
| **203** | **Abuku 1-M2** | **Olevolosi – M2** | **53.53** | **MLP-like** | **Soltu.DM.09G027690.1** | **Environmental Information Processing ++** | **439** | **6.0** | **17.3** | **21** | **41.7** | **7** | **L** |  |
| **203** | **Abuku 1-M2** | **Olevolosi – M2** | **53.53** | **MLP-like protein** | **Soltu.DM.09G027720.2** | **Environmental Information Processing ++** | **62** | **5.8** | **17.2** | **4** | **9.7** | **2** | **L** |  |
| **204** | **Abuku 1-M2** | **Olevolosi – M2** | **25.53** | **MLP-like protein** | **Soltu.DM.09G027690.1** | **Environmental Information Processing ++** | **217** | **6.0** | **17.3** | **9** | **14.7** | **3** | **L** |  |
| **204** | **Abuku 1-M2** | **Olevolosi – M2** | **25.53** | **F-box and associated interaction domains-containing protein** | **Soltu.DM.06G021470.1** | **Genetic information processing ++** | **38** | **5.5** | **22.4** | **2** | **12.4** | **2** | **M** |  |
| **294** | **Abuku 1-M2** | **Acc 33 – M1** | **3.02** | **Alcohol dehydrogenase** | **Soltu.DM.04G025720.1** | **Metabolism - Carbohydrate metabolism - Glycolysis / Gluconeogenesis [EC:1.1.1.1]** | **683** | **6.2** | **41.0** | **43** | **37.2** | **15** | **L** |  |
| **294** | **Abuku 1-M2** | **Acc 33 – M1** | **3.02** | **GroES-like zinc-binding dehydrogenase family protein** | **Soltu.DM.09G018820.1** | **Metabolism - Methane metabolism** | **122** | **6.4** | **40.6** | **4** | **5.3** | **2** | **L** |  |
| **294** | **Abuku 1-M2** | **Acc 33 – M1** | **3.02** | **hydroxysteroid dehydrogenase** | **Soltu.DM.06G021080.1** | **Biological processes – Growth and seed production ++** | **119** | **7.8** | **42.9** | **4** | **9.1** | **3** | **L** |  |
| **322** | **Abuku 1-M2** | **Olevolosi – M2** | **2.36** | **Cruciferin** | **Soltu.DM.09G026760.1** | **Seed storage protein ++** | **347** | **6.7** | **58.1** | **21** | **11.5** | **6** | **M** |  |
| **370** | **Abuku 1-M2** | **Olevolosi –M1** | **2.23** | **RmlC-like cupins superfamily protein** | **Soltu.DM.09G021500.1** | **Seed storage protein ++** | **265** | **7.0** | **54.4** | **15** | **15.4** | **5** | **M** |  |
| **370** | **Abuku 1-M2** | **Olevolosi –M1** | **2.23** | **NAD(P)-binding Rossmann-fold superfamily protein** | **Soltu.DM.01G038420.1** | **Genetic information processing - Ubiquitin system ++** | **149** | **9.4** | **37.3** | **6** | **11.7** | **3** | **L** |  |
| **370** | **Abuku 1-M2** | **Olevolosi –M1** | **2.23** | **RmlC-like cupins superfamily protein** | **Soltu.DM.09G021450.1** | **Seed storage protein ++** | **143** | **6.1** | **49.9** | **12** | **7.9** | **5** | **M** |  |
| **446** | **Abuku 1-M2** | **Acc 33 – M1** | **14.17** | **Argonaute family protein** | **Soltu.DM.01G005850.1** | **Genetic information processing - Messenger RNA biogenesis** | **1306** | **9.7** | **101.7** | **54** | **26.4** | **27** | **L** |  |
| **446** | **Abuku 1-M2** | **Acc 33 – M1** | **14.17** | **Argonaute family protein** | **Soltu.DM.06G028860.1** | **Genetic information processing - Messenger RNA biogenesis** | **1087** | **9.7** | **101.9** | **46** | **23.2** | **22** | **L** |  |
| **446** | **Abuku 1-M2** | **Acc 33 – M1** | **14.17** | **Argonaute family protein** | **Soltu.DM.01G035930.1** | **Genetic information processing - Messenger RNA biogenesis** | **203** | **9.9** | **90.5** | **7** | **4.8** | **4** | **L** |  |
| **400** | **Abuku 1-M2** | **Acc 33 – M2** | **3.30** | **Cruciferin** | **Soltu.DM.09G026760.1** | **Seed storage protein ++** | **291** | **6.7** | **58.1** | **8** | **7.4** | **3** | **M** |  |
| **400** | **Abuku 1-M2** | **Acc 33 – M2** | **3.30** | **RmlC-like cupins superfamily protein** | **Soltu.DM.03G000660.1** | **Seed storage protein ++** | **130** | **9.6** | **14.7** | **8** | **13.8** | **3** | **L** |  |
| **400** | **Abuku 1-M2** | **Acc 33 – M2** | **3.30** | **RmlC-like cupins superfamily protein** | **Soltu.DM.11G025490.1** | **Seed storage protein++** | **106** | **5.6** | **57.0** | **3** | **4.5** | **2** | **M** |  |
| **400** | **Abuku 1-M2** | **Acc 33 – M2** | **3.30** | **RmlC-like cupins superfamily protein** | **Soltu.DM.09G021500.1** | **Seed storage protein ++** | **99** | **7.0** | **54.4** | **10** | **3.7** | **2** | **M** |  |
| **400** | **Abuku 1-M2** | **Acc 33 – M2** | **3.30** | **1-cysteine peroxiredoxin** | **Soltu.DM.03G013100.1** | **Biological processes – ROS detoxification ++** | **86** | **6.1** | **24.2** | **3** | **8.2** | **2** | **L** |  |
| **97** | **Abuku 1-M2** | **Acc 33 – M1** | **3.95** | **ATP synthase alpha/beta family protein** | **Soltu.DM.05G004470.1** | **Metabolism - Energy metabolism** | **717** | **5.6** | **59.5** | **27** | **22.8** | **11** | **L** |  |
| **97** | **Abuku 1-M2** | **Acc 33 – M1** | **3.95** | **Cruciferin** | **Soltu.DM.09G026760.1** | **Seed storage protein ++** | **84** | **6.7** | **58.1** | **4** | **3.3** | **2** | **L** |  |
| **97** | **Abuku 1-M2** | **Acc 33 – M1** | **3.95** | **S-adenosyl-L-homocysteine hydrolase** | **Soltu.DM.09G029630.1** | **Metabolism - Amino acid metabolism - Cysteine and methionine metabolism** | **77** | **5.6** | **53.2** | **3** | **2.9** | **2** | **L** |  |
| **381** | **Abuku 1-M2** | **Acc 33 – M1** | **6.27** | **Protein phosphatase 2C** | **Soltu.DM.07G024120.1** | **Protein families: metabolism -Protein phosphatases and associated proteins** | **288** | **5.6** | **30.9** | **10** | **17.7** | **5** | **5** |  |
| **381** | **Abuku 1-M2** | **Acc 33 – M1** | **6.27** | **Vicilin** | **Soltu.DM.11G025490.1** | **Seed storage protein ++** | **270** | **5.6** | **57.0** | **6** | **11.2** | **5** | **5** |  |
| **381** | **Abuku 1-M2** | **Acc 33 – M1** | **6.27** | **RmlC-like cupins superfamily protein** | **Soltu.DM.09G021500.1** | **Seed storage protein ++** | **165** | **7.0** | **54.4** | **8** | **7.9** | **3** | **2** |  |
| **109** | **Abuku 1-M2** | **Olevolosi M2** | **2.78** | **Alanine-2-oxoglutarate aminotransferase** | **Soltu.DM.01G003360.1** | **Metabolism - Amino acid metabolism - Alanine, aspartate and glutamate metabolism** | **404** | **5.0** | **35.9** | **10** | **20.3** | **6** | **L** |  |
| **109** | **Abuku 1-M2** | **Olevolosi M2** | **2.78** | **Alanine-2-oxoglutarate aminotransferase** | **Soltu.DM.05G009660.1** | **Metabolism - Amino acid metabolism - Alanine, aspartate and glutamate metabolism** | **285** | **6.8** | **53.4** | **8** | **11.2** | **6** | **L** |  |
| **109** | **Abuku 1-M2** | **Olevolosi M2** | **2.78** | **Hypothetical protein** | **Soltu.DM.04G033200.1** | **Unknown ++** | **63** | **5.3** | **8.8** | **2** | **8.9** | **2** | **L** |  |
| **109** | **Abuku 1-M2** | **Olevolosi M2** | **2.78** | **P-loop containing nucleoside triphosphate hydrolases superfamily protein** | **Soltu.DM.06G018790.1** | **Genetic information processing - Chromosome and associated proteins ++** | **61** | **5.4** | **49.7** | **3** | **4.4** | **2** | **L** |  |
| **109** | **Abuku 1-M2** | **Olevolosi M2** | **2.78** | **Insulinase (Peptidase family M16) protein** | **Soltu.DM.05G014230.1** | **Metabolism - Peptidases and inhibitors** | **56** | **6.0** | **54.5** | **2** | **4.0** | **2** | **L** |  |
| **164** | **Abuku 1-M2** | **Acc 33 – M1** | **1.85** | **Glutathione S-transferase, C-terminal-like;Translation elongation factor EF1B/ribosomal protein S6** | **Soltu.DM.01G037330.1** | **Environmental information processing – biological defence response ++** | **268** | **4.3** | **25.3** | **12** | **29.7** | **7** | **L** |  |
| **164** | **Abuku 1-M2** | **Acc 33 – M1** | **1.85** | **Translation elongation factor EF1B/ribosomal protein S6 family protein** | **Soltu.DM.11G025220.1** | **Genetic Information Processing - Translation - RNA transport and biogenesis ++** | **140** | **4.4** | **24.5** | **6** | **13.2** | **3** | **L** |  |
| **72** | **Abuku 1-M2** | **Olevolosi – M1** | **1.84** | **RmlC-like cupins superfamily protein** | **Soltu.DM.09G021500.1** | **Seed storage protein ++** | **156** | **7.0** | **54.4** | **6** | **7.9** | **3** | **L** |  |
| **72** | **Abuku 1-M2** | **Olevolosi – M1** | **1.84** | **Thiamine pyrophosphate dependent pyruvate decarboxylase family protein** | **Soltu.DM.10G019450.1** | **Metabolism - Carbohydrate metabolism - Glycolysis / Gluconeogenesis** | **147** | **5.7** | **65.5** | **8** | **5.0** | **3** | **L** |  |
| **72** | **Abuku 1-M2** | **Olevolosi – M1** | **1.84** | **RmlC-like cupins superfamily protein** | **Soltu.DM.03G000660.1** | **Seed storage protein ++** | **110** | **9.6** | **14.7** | **6** | **12.2** | **2** | **L** |  |
| **72** | **Abuku 1-M2** | **Olevolosi – M1** | **1.84** | **Cruciferin** | **Soltu.DM.03G000650.1** | **Storage protein ++** | **86** | **7.9** | **42.6** | **5** | **2.4** | **2** | **L** |  |
| **72** | **Abuku 1-M2** | **Olevolosi – M1** | **1.84** | **RmlC-like cupins superfamily protein** | **Soltu.DM.09G021460.1** | **Seed storage protein ++** | **83** | **7.9** | **53.4** | **5** | **4.6** | **2** | **L** |  |
| **72** | **Abuku 1-M2** | **Olevolosi – M1** | **1.84** | **Cruciferin** | **Soltu.DM.09G026760.1** | **Seed storage protein ++** | **78** | **6.7** | **58.1** | **3** | **3.9** | **2** | **L** |  |

**A** = SpotID as defined by the Delta2D software from DECODON on the master gel from the 2D PAGE gels. Corresponding spots of all gels are labelled with the same ID.

**B** = Accession for which the proteins in the row were identified.

**C** = Proteins were identified from spots picked from the following 2D PAGE gel. Ole stands for Olevolosi, Abu stands for Abuku and Acc33 stands for Accession 33, M1 = maturity state 1 (green berries), M2 = maturity state 2 (purple berries).

**D** = Regulation of a spot according to the comparison between groups. Regulation is given as the ratio between spot abundance (M2 / M1).

**E** = Functional classification mainly following the KEGG Pathway Database (++ = if no classification was automatically annotated, the proteins were manually classified).

**F** = The protein score obtained via the MASCOT search algorithm (www.matrixscience.com) against a potato protein database, which was based upon the sequences from *Solanum tuberosum* group Phureja DM1-3 v 6.1, which was completely sequenced by the Potato Genome Consortium in 2020.

**G** = Calculated PI obtained via the MASCOT search algorithm (www.matrixscience.com) against a potato protein database.

**H** = Calculated MW obtained via the MASCOT search algorithm (www.matrixscience.com) against a potato protein database.

**I** = Number of peptides matched to the protein through the database search.

**J** = Sequence coverage in %.

**K** = Unique peptides matched to the sequence. Only proteins with at least two unique peptide were considered true hits.

**L** = Molecular weight (MW) in gel as compared to the theoretically expected MW.

**M**: MW in gel corresponding to the theoretically expected MW ± 15 kDa

**S**: MW in gel lower than theoretically expected

**L**: MW in gel larger than theoretically expected

**M** = Mean relative spot volume obtained according to three gels of M1 seeds or M2 seeds illustrated by graphs. The first bar (orange) represents the mean normalized spot volume in the gels of M1 seeds of Accession 33. The second bar (light orange) represents the mean normalized spot volume in the gels of the M2 seeds from Accession 33. The third bar (green) stands for the mean normalized spot volume in the gels of the M1 seeds of Abuku 1. The fourth bar (light green) represents the mean normalized spot volume in the gels of the M2 seeds of Abuku 1. The fifth bar (purple) represents the mean normalized spot volume in the gels of the M1 seeds from Olevolosi. The sixth bar (light purple) stands for the mean normalized spot volume in the gels of the M2 seeds of Olevolosi.

Tabel 5: Identification of proteins from spots with changed abundance in comparison 1 between M2 and M1 of Olevolosi. A Student's t-test was performed (p-value ≤ 0.05) to determine significant changes in spot volume based on normalised relative spot volume. Only alterations of at least 1.5-fold in spot volume were considered to represent true alterations in protein level. Analysis was performed with Delta2D by DECODON.

| **Spot ID^A^** | **Accession^B^** | **Identified from Gel^C^** | **Reg^D^** | **Protein name** | **PGSC numbers** | **KEGG^E^** | **Score^F^** | **PI Calc^G^** | **MW Calc ^H^** | **Pep ^I^** | **SC [%]^J^** | **UPep^K^** | **Size^L^** | **Normalized spot volume^M^** |
| --- | --- | --- | --- | --- | --- | --- | --- | --- | --- | --- | --- | --- | --- | --- |
| **60** | **Olevolosi-M1** | **Olevolosi-M1** | **0.49** | **Glycosyl hydrolase family protein** | **Soltu.DM.06G029150.1** | **Metabolism - Hydrolases –Glycosylases- [EC 3.2.1.21] ++** | **224** | **5.7** | **55.9** | **8** | **11.7** | **5** | **L** |  |
| **60** | **Olevolosi-M1** | **Olevolosi-M1** | **0.49** | **Glycosyl hydrolase family protein** | **Soltu.DM.06G029150.2** | **Metabolism - Hydrolases –Glycosylases- [EC 3.2.1.21] ++** | **220** | **6.9** | **66.0** | **10** | **9.8** | **5** | **L** |  |
| **60** | **Olevolosi-M1** | **Olevolosi-M1** | **0.49** | **Glycosyl hydrolase family protein** | **Soltu.DM.06G029160.1** | **Metabolism - Hydrolases –Glycosylases- [EC 3.2.1.21] ++** | **157** | **8.5** | **68.7** | **5** | **6.5** | **3** | **L** |  |
| **71** | **Olevolosi-M1** | **Olevolosi-M1** | **0.62** | **RmlC-like cupins superfamily protein** | **Soltu.DM.09G021500.1** | **Seed storage protein ++** | **232** | **7.0** | **54.4** | **10** | **10.8** | **4** | **L** |  |
| **71** | **Olevolosi-M1** | **Olevolosi-M1** | **0.62** | **Succinate dehydrogenase 1-1** | **Soltu.DM.02G025210.1** | **Metabolism -Carbohydrate metabolism -Citrate cycle (TCA cycle)** | **185** | **6.1** | **69.2** | **4** | **6.5** | **4** | **L** |  |
| **71** | **Olevolosi-M1** | **Olevolosi-M1** | **0.62** | **Thiamine pyrophosphate dependent pyruvate decarboxylase family protein** | **Soltu.DM.10G019450.1** | **Metabolism - Carbohydrate metabolism - Glycolysis / Gluconeogenesis** | **126** | **5.7** | **65.5** | **6** | **5.0** | **3** | **L** |  |
| **334** | **Olevolosi-M1** | **Olevolosi-M1** | **0.31** | **RmlC-like cupins superfamily protein** | **Soltu.DM.09G021500.1** | **Seed storage protein ++** | **77** | **7.0** | **54.4** | **4** | **7.9** | **3** | **L** |  |
| **370** | **Olevolosi-M1** | **Olevolosi-M1** | **0.50** | **RmlC-like cupins superfamily protein** | **Soltu.DM.09G021500.1** | **Seed storage protein ++** | **265** | **7.0** | **54.4** | **15** | **15.4** | **5** | **M** |  |
| **370** | **Olevolosi-M1** | **Olevolosi-M1** | **0.50** | **NAD(P)-binding Rossmann-fold superfamily protein** | **Soltu.DM.01G038420.1** | **Genetic information processing - Ubiquitin system ++** | **149** | **9.4** | **37.3** | **6** | **11.7** | **3** | **L** |  |
| **370** | **Olevolosi-M1** | **Olevolosi-M1** | **0.50** | **RmlC-like cupins superfamily protein** | **Soltu.DM.09G021450.1** | **Seed storage protein ++** | **143** | **6.1** | **49.9** | **12** | **7.9** | **5** | **M** |  |
| **394** | **Olevolosi-M1** | **Olevolosi-M1** | **0.04** | **RmlC-like cupins superfamily protein** | **Soltu.DM.11G025490.1** | **Seed storage protein ++** | **166** | **5.6** | **57.0** | **8** | **6.9** | **3** | **M** |  |
| **394** | **Olevolosi-M1** | **Olevolosi-M1** | **0.04** | **Triosephosphate isomerase** | **Soltu.DM.04G007490.1** | **Metabolism - Carbohydrate metabolism - Glycolysis / Gluconeogenesis ++** | **103** | **5.7** | **27.0** | **2** | **8.7** | **2** | **L** |  |
| **394** | **Olevolosi-M1** | **Olevolosi-M1** | **0.04** | **20S proteasome alpha subunit G1** | **Soltu.DM.10G026260.1** | **Genetic Information Processing - Folding, sorting and degradation - Proteasome** | **73** | **6.1** | **27.1** | **3** | **7.6** | **2** | **L** |  |
| **423** | **Olevolosi-M1** | **Olevolosi-M1** | **0.10** | **glyceraldehyde-3-phosphate dehydrogenase C2** | **Soltu.DM.05G010790.1** | **Metabolism - Carbohydrate metabolism - Glycolysis / Gluconeogenesis ++** | **454** | **6.4** | **36.6** | **23** | **25.4** | **11** | **L** |  |
| **423** | **Olevolosi-M1** | **Olevolosi-M1** | **0.10** | **RmlC-like cupins superfamily protein** | **Soltu.DM.09G021450.1** | **Seed storage protein ++** | **78** | **6.1** | **49.9** | **2** | **3.4** | **2** | **L** |  |
| **424** | **Olevolosi-M1** | **Olevolosi-M1** | **0.02** | **Glyceraldehyde-3-phosphate dehydrogenase C subunit** | **Soltu.DM.03G024400.1** | **Metabolism - Carbohydrate metabolism - Glycolysis / Gluconeogenesis ++** | **405** | **7.7** | **36.7** | **14** | **24.0** | **10** | **L** |  |
| **424** | **Olevolosi-M1** | **Olevolosi-M1** | **0.02** | **Glyceraldehyde-3-phosphate dehydrogenase C subunit** | **Soltu.DM.06G027160.1** | **Metabolism - Carbohydrate metabolism - Glycolysis / Gluconeogenesis ++** | **357** | **7.7** | **36.6** | **12** | **19.9** | **9** | **L** |  |
| **424** | **Olevolosi-M1** | **Olevolosi-M1** | **0.02** | **glyceraldehyde-3-phosphate dehydrogenase C2** | **Soltu.DM.05G010790.1** | **Metabolism - Carbohydrate metabolism - Glycolysis / Gluconeogenesis ++** | **339** | **6.4** | **36.6** | **13** | **18.3** | **9** | **L** |  |
| **548** | **Olevolosi-M1** | **Olevolosi-M1** | **0.14** | **RmlC-like cupins superfamily protein** | **Soltu.DM.09G021500.1** | **Seed storage protein ++** | **180** | **7.0** | **54.4** | **5** | **10.0** | **3** | **M** |  |
| **548** | **Olevolosi-M1** | **Olevolosi-M1** | **0.14** | **Cruciferin** | **Soltu.DM.09G026760.1** | **Seed storage protein ++** | **66** | **6.7** | **58.1** | **5** | **3.7** | **2** | **S** |  |
| **454** | **Olevolosi-M1** | **Olevolosi-M1** | **0.57** | **Glycosyl hydrolase family protein** | **Soltu.DM.06G029150.1** | **Metabolism - Hydrolases –Glycosylases- [EC 3.2.1.21] ++** | **539** | **5.7** | **55.9** | **20** | **16.2** | **9** | **L** |  |
| **454** | **Olevolosi-M1** | **Olevolosi-M1** | **0.57** | **Glycosyl hydrolase family protein** | **Soltu.DM.06G029150.2** | **Metabolism - Hydrolases –Glycosylases- [EC 3.2.1.21] ++** | **478** | **6.9** | **66.0** | **22** | **13.5** | **8** | **L** |  |
| **454** | **Olevolosi-M1** | **Olevolosi-M1** | **0.57** | **Glycosyl hydrolase family protein** | **Soltu.DM.06G029160.1** | **Metabolism - Hydrolases –Glycosylases- [EC 3.2.1.21] ++** | **430** | **8.5** | **68.7** | **17** | **12.1** | **8** | **L** |  |
| **158** | **Olevolosi-M1** | **Olevolosi-M1** | **0.18** | **RmlC-like cupins superfamily protein** | **Soltu.DM.11G025490.1** | **Seed storage protein ++** | **143** | **5.6** | **57.0** | **5** | **6.7** | **3** | **M** |  |
| **541** | **Olevolosi-M1** | **Olevolosi-M1** | **0.17** | **Voltage dependent anion channel** | **Soltu.DM.03G008530.1** | **Protein families: genetic information processing - Mitochondrial biogenesis - Protein families: signalling and cellular processes - Ion channels** | **51** | **8.8** | **29.4** | **2** | **6.2** | **2** | **L** |  |
| **359** | **Olevolosi-M1** | **Olevolosi-M1** | **0.16** | **RmlC-like cupins superfamily protein** | **Soltu.DM.09G021500.1** | **Seed storage protein ++** | **104** | **7.0** | **54.4** | **8** | **7.9** | **3** | **L** |  |
| **359** | **Olevolosi-M1** | **Olevolosi-M1** | **0.16** | **Lactate/malate dehydrogenase family protein** | **Soltu.DM.09G026740.1** | **Metabolism – citric acid cycle++** | **85** | **5.9** | **35.4** | **5** | **7.2** | **3** | **L** |  |
| **359** | **Olevolosi-M1** | **Olevolosi-M1** | **0.16** | **NAD(P)-binding Rossmann-fold superfamily protein** | **Soltu.DM.01G038420.1** | **Genetic information processing - Ubiquitin system ++** | **66** | **9.4** | **37.3** | **3** | **7.9** | **2** | **L** |  |
| **309** | **Olevolosi-M1** | **Olevolosi-M1** | **0.08** | **Cupin family protein** | **Soltu.DM.09G024720.1** | **Seed storage protein ++** | **215** | **8.6** | **65.7** | **5** | **6.0** | **4** | **L** |  |
| **309** | **Olevolosi-M1** | **Olevolosi-M1** | **0.08** | **Cupin family protein** | **Soltu.DM.09G024710.1** | **Seed storage protein ++** | **132** | **5.9** | **42.5** | **5** | **5.6** | **2** | **L** |  |
| **137** | **Olevolosi-M1** | **Olevolosi-M1** | **0.00** | **Cruciferin** | **Soltu.DM.09G026760.1** | **Seed storage protein ++** | **106** | **6.7** | **58.1** | **3** | **3.9** | **2** | **L** |  |
| **137** | **Olevolosi-M1** | **Olevolosi-M1** | **0.00** | **RmlC-like cupins superfamily protein** | **Soltu.DM.09G021450.1** | **Seed storage protein ++** | **84** | **6.1** | **49.9** | **3** | **3.4** | **2** | **L** |  |
| **137** | **Olevolosi-M1** | **Olevolosi-M1** | **0.00** | **Glyceraldehyde-3-phosphate dehydrogenase C subunit** | **Soltu.DM.03G024400.1** | **Metabolism - Carbohydrate metabolism - Glycolysis / Gluconeogenesis ++** | **41** | **7.7** | **36.7** | **2** | **4.5** | **2** | **L** |  |
| **411** | **Olevolosi-M1** | **Olevolosi-M1** | **0.21** | **Cruciferin** | **Soltu.DM.09G026760.1** | **Seed storage protein ++** | **168** | **6.7** | **58.1** | **9** | **6.4** | **3** | **L** |  |
| **84** | **Olevolosi-M1** | **Olevolosi-M1** | **0.29** | **Phosphoglycerate mutase, 2,3-bisphosphoglycerate-independent** | **Soltu.DM.07G014610.1** | **Metabolism - Carbohydrate metabolism - Glycolysis / Gluconeogenesis ++** | **176** | **5.3** | **61.2** | **6** | **7.5** | **5** | **L** |  |
| **84** | **Olevolosi-M1** | **Olevolosi-M1** | **0.29** | **Heat-shock protein 70T-2** | **Soltu.DM.09G024280.1** | **Genetic information processing - Chaperones and folding catalysts** | **150** | **5.5** | **62.4** | **6** | **8.6** | **4** | **L** |  |
| **84** | **Olevolosi-M1** | **Olevolosi-M1** | **0.29** | **RmlC-like cupins superfamily protein** | **Soltu.DM.09G021460.1** | **Seed storage protein ++** | **100** | **7.9** | **53.4** | **3** | **4.6** | **2** | **L** |  |
| **84** | **Olevolosi-M1** | **Olevolosi-M1** | **0.29** | **RmlC-like cupins superfamily protein** | **Soltu.DM.09G021450.1** | **Seed storage protein ++** | **85** | **6.1** | **49.9** | **3** | **3.4** | **2** | **L** |  |
| **84** | **Olevolosi-M1** | **Olevolosi-M1** | **0.29** | **Thiamine pyrophosphate dependent pyruvate decarboxylase family protein** | **Soltu.DM.10G019450.1** | **Metabolism - Carbohydrate metabolism - Glycolysis / Gluconeogenesis** | **82** | **5.7** | **65.5** | **2** | **3.0** | **2** | **L** |  |
| **463** | **Olevolosi-M1** | **Olevolosi-M1** | **0.29** | **Glycosyl hydrolase family protein** | **Soltu.DM.06G029150.1** | **Metabolism - Hydrolases –Glycosylases- [EC 3.2.1.21] ++** | **106** | **5.7** | **55.9** | **4** | **4.3** | **2** | **L** |  |
| **463** | **Olevolosi-M1** | **Olevolosi-M1** | **0.29** | **Glycosyl hydrolase family protein** | **Soltu.DM.06G029150.2** | **Metabolism - Hydrolases –Glycosylases- [EC 3.2.1.21] ++** | **66** | **6.9** | **66.0** | **3** | **3.5** | **2** | **L** |  |
| **406** | **Olevolosi-M1** | **Olevolosi-M1** | **0.11** | **RmlC-like cupins superfamily protein** | **Soltu.DM.11G025490.1** | **Seed storage protein ++** | **118** | **5.6** | **57.0** | **4** | **3.9** | **2** | **M** |  |
| **365** | **Olevolosi-M1** | **Olevolosi-M1** | **0.47** | **RmlC-like cupins superfamily protein** | **Soltu.DM.09G021500.1** | **Seed storage protein ++** | **375** | **7.0** | **54.4** | **27** | **18.3** | **6** | **M** |  |
| **354** | **Olevolosi-M1** | **Olevolosi-M1** | **0.38** | **Glyceraldehyde-3-phosphate dehydrogenase C2** | **Soltu.DM.05G010790.1** | **Metabolism - Carbohydrate metabolism - Glycolysis / Gluconeogenesis ++** | **599** | **6.4** | **36.6** | **24** | **29.3** | **12** | **L** |  |
| **354** | **Olevolosi-M1** | **Olevolosi-M1** | **0.38** | **Glyceraldehyde-3-phosphate dehydrogenase C subunit** | **Soltu.DM.06G027160.1** | **Metabolism - Carbohydrate metabolism - Glycolysis / Gluconeogenesis ++** | **401** | **7.7** | **36.6** | **17** | **22.8** | **10** | **L** |  |
| **354** | **Olevolosi-M1** | **Olevolosi-M1** | **0.38** | **RmlC-like cupins superfamily protein** | **Soltu.DM.09G021500.1** | **Seed storage protein ++** | **78** | **7.0** | **54.4** | **3** | **3.1** | **2** | **L** |  |
| **72** | **Olevolosi-M1** | **Olevolosi-M1** | **0.35** | **RmlC-like cupins superfamily protein** | **Soltu.DM.09G021500.1** | **Seed storage protein ++** | **156** | **7.0** | **54.4** | **6** | **7.9** | **3** | **L** |  |
| **72** | **Olevolosi-M1** | **Olevolosi-M1** | **0.35** | **Thiamine pyrophosphate dependent pyruvate decarboxylase family protein** | **Soltu.DM.10G019450.1** | **Metabolism - Carbohydrate metabolism - Glycolysis / Gluconeogenesis** | **147** | **5.7** | **65.5** | **8** | **5.0** | **3** | **L** |  |
| **72** | **Olevolosi-M1** | **Olevolosi-M1** | **0.35** | **RmlC-like cupins superfamily protein** | **Soltu.DM.03G000660.1** | **Seed storage protein ++** | **110** | **9.6** | **14.7** | **6** | **12.2** | **2** | **L** |  |
| **72** | **Olevolosi-M1** | **Olevolosi-M1** | **0.35** | **Cruciferin** | **Soltu.DM.03G000650.1** | **Storage protein ++** | **86** | **7.9** | **42.6** | **5** | **2.4** | **2** | **L** |  |
| **72** | **Olevolosi-M1** | **Olevolosi-M1** | **0.35** | **RmlC-like cupins superfamily protein** | **Soltu.DM.09G021460.1** | **Seed storage protein ++** | **83** | **7.9** | **53.4** | **5** | **4.6** | **2** | **L** |  |
| **72** | **Olevolosi-M1** | **Olevolosi-M1** | **0.35** | **Cruciferin** | **Soltu.DM.09G026760.1** | **Seed storage protein ++** | **78** | **6.7** | **58.1** | **3** | **3.9** | **2** | **L** |  |
| **75** | **Olevolosi-M1** | **Olevolosi-M1** | **0.41** | **Thiamine pyrophosphate dependent pyruvate decarboxylase family protein** | **Soltu.DM.10G019450.1** | **Metabolism - Carbohydrate metabolism - Glycolysis / Gluconeogenesis** | **197** | **5.7** | **65.5** | **9** | **6.3** | **4** | **L** |  |
| **75** | **Olevolosi-M1** | **Olevolosi-M1** | **0.41** | **RmlC-like cupins superfamily protein** | **Soltu.DM.09G021500.1** | **Seed storage protein ++** | **112** | **7.0** | **54.4** | **3** | **6.7** | **2** | **L** |  |
| **26** | **Olevolosi-M2** | **Olevolosi-M2** | **2.17** | **Pyruvate orthophosphate dikinase** | **Soltu.DM.01G023530.1** | **Metabolism [EC 2.7.9.19] ++** | **1621** | **5.1** | **93.6** | **69** | **31.2** | **28** | **L** |  |
| **26** | **Olevolosi-M2** | **Olevolosi-M2** | **2.17** | **Conserved hypothetical protein** | **Soltu.DM.11G001270.1** | **Unknown ++** | **50** | **7.8** | **25.5** | **2** | **5.5** | **2** | **L** |  |
| **79** | **Olevolosi-M2** | **Olevolosi-M2** | **2.21** | **Late embryogenesis abundant domain-containing protein / LEA domain-containing protein** | **Soltu.DM.07G020570.1** | **Metabolism ++** | **686** | **5.2** | **56.3** | **52** | **21.8** | **12** | **L** |  |
| **91** | **Olevolosi-M2** | **Olevolosi-M2** | **8.98** | **Late embryogenesis abundant domain-containing protein / LEA domain-containing protein** | **Soltu.DM.07G020570.1** | **Metabolism ++** | **542** | **5.2** | **56.3** | **36** | **19.5** | **11** | **L** |  |
| **92** | **Olevolosi-M2** | **Olevolosi-M2** | **14.97** | **Late embryogenesis abundant domain-containing protein / LEA domain-containing protein** | **Soltu.DM.07G020570.1** | **Metabolism ++** | **672** | **5.2** | **56.3** | **35** | **22.4** | **12** | **L** |  |
| **92** | **Olevolosi-M2** | **Olevolosi-M2** | **14.97** | **TCP-1/cpn60 chaperonin family protein** | **Soltu.DM.01G026570.1** | **Genetic information processing - Chaperones and folding catalysts** | **215** | **5.2** | **58.3** | **7** | **15.7** | **7** | **L** |  |
| **92** | **Olevolosi-M2** | **Olevolosi-M2** | **14.97** | **2-oxoglutarate (2OG) and Fe(II)-dependent oxygenase superfamily protein** | **Soltu.DM.03G030400.1** | **Metabolism - Flavonoid biosynthesis ++** | **38** | **5.7** | **185.5** | **3** | **0.9** | **2** | **S** |  |
| **92** | **Olevolosi-M2** | **Olevolosi-M2** | **14.97** | **hypothetical protein** | **Soltu.DM.02G012540.1** | **Unknown ++** | **34** | **9.2** | **81.2** | **3** | **2.4** | **2** | **L** |  |
| **95** | **Olevolosi-M2** | **Olevolosi-M2** | **2.32** | **RmlC-like cupins superfamily protein** | **Soltu.DM.09G021500.1** | **Seed storage protein ++** | **155** | **7.0** | **54.4** | **12** | **7.3** | **4** | **L** |  |
| **95** | **Olevolosi-M2** | **Olevolosi-M2** | **2.32** | **RmlC-like cupins superfamily protein** | **Soltu.DM.03G000660.1** | **Seed storage protein ++** | **125** | **9.6** | **14.7** | **4** | **13.8** | **3** | **L** |  |
| **95** | **Olevolosi-M2** | **Olevolosi-M2** | **2.32** | **Glutamate decarboxylase** | **Soltu.DM.03G019160.3** | **Metabolism - Carbohydrate metabolism - Amino acid metabolism** | **117** | **5.7** | **56.7** | **5** | **6.0** | **3** | **L** |  |
| **95** | **Olevolosi-M2** | **Olevolosi-M2** | **2.32** | **Cruciferin** | **Soltu.DM.09G026760.1** | **Seed storage protein ++** | **94** | **6.7** | **58.1** | **4** | **3.9** | **2** | **L** |  |
| **95** | **Olevolosi-M2** | **Olevolosi-M2** | **2.32** | **Cupin family protein** | **Soltu.DM.09G024720.1** | **Seed storage protein ++** | **77** | **8.6** | **65.7** | **3** | **3.5** | **2** | **L** |  |
| **95** | **Olevolosi-M2** | **Olevolosi-M2** | **2.32** | **DNA binding** | **Soltu.DM.11G004880.1** | **Unknown ++** | **63** | **4.7** | **56.6** | **4** | **1.7** | **2** | **L** |  |
| **95** | **Olevolosi-M2** | **Olevolosi-M2** | **2.32** | **RmlC-like cupins superfamily protein** | **Soltu.DM.09G021460.1** | **Seed storage protein ++** | **53** | **7.9** | **53.4** | **3** | **4.0** | **2** | **L** |  |
| **95** | **Olevolosi-M2** | **Olevolosi-M2** | **2.32** | **Structural maintenance of chromosomes (SMC) family protein** | **Soltu.DM.06G034250.1** | **Genetic information processing - Chromosome and associated proteins** | **45** | **9.1** | **141.0** | **3** | **1.3** | **2** | **S** |  |
| **98** | **Olevolosi-M2** | **Olevolosi-M2** | **3.48** | **RmlC-like cupins superfamily protein** | **Soltu.DM.09G021500.1** | **Seed storage protein ++** | **143** | **7.0** | **54.4** | **11** | **5.0** | **3** | **L** |  |
| **104** | **Olevolosi-M2** | **Olevolosi-M2** | **2.94** | **Embryonic cell protein** | **Soltu.DM.09G004550.1** | **Unknown ++** | **191** | **5.3** | **43.1** | **12** | **8.0** | **3** | **L** |  |
| **109** | **Olevolosi-M2** | **Olevolosi-M2** | **1.64** | **Alanine-2-oxoglutarate aminotransferase** | **Soltu.DM.01G003360.1** | **Metabolism - Amino acid metabolism - Alanine, aspartate and glutamate metabolism** | **404** | **5.0** | **35.9** | **10** | **20.3** | **6** | **L** |  |
| **109** | **Olevolosi-M2** | **Olevolosi-M2** | **1.64** | **Alanine-2-oxoglutarate aminotransferase** | **Soltu.DM.05G009660.1** | **Metabolism - Amino acid metabolism - Alanine, aspartate and glutamate metabolism** | **285** | **6.8** | **53.4** | **8** | **11.2** | **6** | **L** |  |
| **109** | **Olevolosi-M2** | **Olevolosi-M2** | **1.64** | **Hypothetical protein** | **Soltu.DM.04G033200.1** | **Unknown ++** | **63** | **5.3** | **8.8** | **2** | **8.9** | **2** | **L** |  |
| **109** | **Olevolosi-M2** | **Olevolosi-M2** | **1.64** | **P-loop containing nucleoside triphosphate hydrolases superfamily protein** | **Soltu.DM.06G018790.1** | **Genetic information processing - Chromosome and associated proteins ++** | **61** | **5.4** | **49.7** | **3** | **4.4** | **2** | **L** |  |
| **109** | **Olevolosi-M2** | **Olevolosi-M2** | **1.64** | **Insulinase (Peptidase family M16) protein** | **Soltu.DM.05G014230.1** | **Metabolism - Peptidases and inhibitors** | **56** | **6.0** | **54.5** | **2** | **4.0** | **2** | **L** |  |
| **113** | **Olevolosi-M2** | **Olevolosi-M2** | **1.98** | **Embryonic cell protein** | **Soltu.DM.09G004550.1** | **Unknown ++** | **520** | **5.3** | **43.1** | **25** | **18.8** | **7** | **L** |  |
| **113** | **Olevolosi-M2** | **Olevolosi-M2** | **1.98** | **Enolase** | **Soltu.DM.03G028540.1** | **Metabolism - Carbohydrate metabolism - Glycolysis / Gluconeogenesis ++** | **138** | **6.1** | **52.1** | **4** | **6.4** | **3** | **L** |  |
| **130** | **Olevolosi-M2** | **Olevolosi-M2** | **31.11** | **Phosphoglycerate kinase** | **Soltu.DM.07G028580.1** | **Metabolism – Glycolysis ++** | **839** | **5.4** | **42.3** | **32** | **47.6** | **15** | **L** |  |
| **130** | **Olevolosi-M2** | **Olevolosi-M2** | **31.11** | **Hydroxysteroid dehydrogenase** | **Soltu.DM.06G021080.1** | **Biological processes – Growth and seed production ++** | **250** | **7.8** | **42.9** | **7** | **16.1** | **5** | **L** |  |
| **130** | **Olevolosi-M2** | **Olevolosi-M2** | **31.11** | **RmlC-like cupins superfamily protein** | **Soltu.DM.09G021500.1** | **Seed storage protein ++** | **74** | **7.0** | **54.4** | **5** | **3.7** | **2** | **L** |  |
| **131** | **Olevolosi-M2** | **Olevolosi-M2** | **3.37** | **Actin** | **Soltu.DM.11G008990.1** | **Signaling and cellular processes - Cytoskeleton proteins** | **683** | **5.2** | **41.8** | **30** | **31.3** | **11** | **L** |  |
| **131** | **Olevolosi-M2** | **Olevolosi-M2** | **3.37** | **Actin** | **Soltu.DM.03G011750.1** | **Signaling and cellular processes - Cytoskeleton proteins** | **661** | **5.2** | **41.7** | **28** | **31.3** | **11** | **L** |  |
| **131** | **Olevolosi-M2** | **Olevolosi-M2** | **3.37** | **Actin-11** | **Soltu.DM.04G007480.1** | **Signaling and cellular processes - Cytoskeleton proteins** | **488** | **5.2** | **41.6** | **23** | **23.6** | **9** | **L** |  |
| **131** | **Olevolosi-M2** | **Olevolosi-M2** | **3.37** | **Phosphoglycerate kinase** | **Soltu.DM.07G028580.1** | **Metabolism – Glycolysis ++** | **156** | **5.4** | **42.3** | **2** | **6.7** | **2** | **L** |  |
| **131** | **Olevolosi-M2** | **Olevolosi-M2** | **3.37** | **Hydroxysteroid dehydrogenase** | **Soltu.DM.06G021080.1** | **Biological processes – Growth and seed production ++** | **95** | **7.8** | **42.9** | **4** | **5.7** | **2** | **L** |  |
| **131** | **Olevolosi-M2** | **Olevolosi-M2** | **3.37** | **RmlC-like cupins superfamily protein** | **Soltu.DM.09G021500.1** | **Seed storage protein ++** | **94** | **7.0** | **54.4** | **4** | **5.0** | **3** | **L** |  |
| **154** | **Olevolosi-M2** | **Olevolosi-M2** | **3.71** | **RmlC-like cupins superfamily protein** | **Soltu.DM.09G021460.1** | **Seed storage protein ++** | **299** | **7.9** | **53.4** | **16** | **12.4** | **4** | **M** |  |
| **154** | **Olevolosi-M2** | **Olevolosi-M2** | **3.71** | **RmlC-like cupins superfamily protein** | **Soltu.DM.03G000660.1** | **Seed storage protein ++** | **167** | **9.6** | **14.7** | **11** | **13.8** | **3** | **L** |  |
| **154** | **Olevolosi-M2** | **Olevolosi-M2** | **3.71** | **Cruciferin** | **Soltu.DM.03G000650.1** | **Storage protein ++** | **121** | **7.9** | **42.6** | **5** | **2.4** | **2** | **M** |  |
| **154** | **Olevolosi-M2** | **Olevolosi-M2** | **3.71** | **RmlC-like cupins superfamily protein** | **Soltu.DM.09G021450.1** | **Seed storage protein ++** | **85** | **6.1** | **49.9** | **2** | **3.4** | **2** | **M** |  |
| **154** | **Olevolosi-M2** | **Olevolosi-M2** | **3.71** | **Cruciferin** | **Soltu.DM.09G026760.1** | **Seed storage protein ++** | **54** | **6.7** | **58.1** | **3** | **2.3** | **2** | **M** |  |
| **157** | **Olevolosi-M2** | **Olevolosi-M2** | **2.52** | **Voltage dependent anion channel** | **Soltu.DM.03G008530.1** | **Protein families: genetic information processing - Mitochondrial biogenesis - Protein families: signaling and cellular processes - Ion channels** | **366** | **8.8** | **29.4** | **18** | **24.6** | **9** | **L** |  |
| **157** | **Olevolosi-M2** | **Olevolosi-M2** | **2.52** | **Voltage dependent anion channel** | **Soltu.DM.01G009390.1** | **Protein families: genetic information processing - Mitochondrial biogenesis - Protein families: signaling and cellular processes - Ion channels** | **241** | **8.7** | **29.4** | **9** | **13.8** | **6** | **L** |  |
| **162** | **Olevolosi-M2** | **Olevolosi-M2** | **33.57** | **RmlC-like cupins superfamily protein** | **Soltu.DM.11G025490.1** | **Seed storage protein ++** | **184** | **5.6** | **57.0** | **6** | **6.7** | **3** | **M** |  |
| **162** | **Olevolosi-M2** | **Olevolosi-M2** | **33.57** | **AGAMOUS-like** | **Soltu.DM.01G019080.1** | **Genetic information processing - Transcription factors ++** | **35** | **9.8** | **20.8** | **2** | **4.4** | **1** | **L** |  |
| **166** | **Olevolosi-M2** | **Olevolosi-M2** | **4.19** | **Late embryogenesis abundant protein (LEA) family protein** | **Soltu.DM.12G002070.1** | **Metabolism** | **384** | **5.4** | **30.0** | **21** | **9.0** | **5** | **L** |  |
| **166** | **Olevolosi-M2** | **Olevolosi-M2** | **4.19** | **NB-ARC domain-containing disease resistance protein** | **Soltu.DM.09G029500.1** | **Environmental information processing – defence responses ++** | **36** | **8.9** | **99.5** | **2** | **2.1** | **2** | **S** |  |
| **169** | **Olevolosi-M2** | **Olevolosi-M2** | **4.61** | **Late embryogenesis abundant protein (LEA) family protein** | **Soltu.DM.12G002070.1** | **Metabolism** | **369** | **5.4** | **30.0** | **17** | **16.6** | **6** | **M** |  |
| **169** | **Olevolosi-M2** | **Olevolosi-M2** | **4.61** | **Glutathione S-transferase family protein** | **Soltu.DM.10G023750.1** | **Metabolism - Metabolism of other amino acids - Glutathione metabolism ++** | **157** | **5.0** | **27.2** | **8** | **23.8** | **5** | **L** |  |
| **169** | **Olevolosi-M2** | **Olevolosi-M2** | **4.61** | **Zinc finger (C3HC4-type RING finger) family protein** | **Soltu.DM.02G026990.1** | **Unknown ++** | **38** | **10.5** | **79.4** | **6** | **2.2** | **2** | **S** |  |
| **175** | **Olevolosi-M2** | **Olevolosi-M2** | **44.25** | **Late embryogenesis abundant protein (LEA) family protein** | **Soltu.DM.12G002070.1** | **Metabolism** | **178** | **5.4** | **30.0** | **10** | **8.3** | **3** | **M** |  |
| **178** | **Olevolosi-M2** | **Olevolosi-M2** | **1.64** | **RmlC-like cupins superfamily protein** | **Soltu.DM.09G021500.1** | **Seed storage protein ++** | **101** | **7.0** | **54.4** | **8** | **3.7** | **2** | **M** |  |
| **178** | **Olevolosi-M2** | **Olevolosi-M2** | **1.64** | **Cupin family protein** | **Soltu.DM.09G024720.1** | **Seed storage protein ++** | **65** | **8.6** | **65.7** | **3** | **4.1** | **2** | **S** |  |
| **178** | **Olevolosi-M2** | **Olevolosi-M2** | **1.64** | **RmlC-like cupins superfamily protein** | **Soltu.DM.03G000660.1** | **Seed storage protein ++** | **43** | **9.6** | **14.7** | **3** | **12.2** | **2** | **L** |  |
| **196** | **Olevolosi-M2** | **Olevolosi-M2** | **1.90** | **HSP20-like chaperones superfamily protein** | **Soltu.DM.06G031840.1** | **Genetic Information Processing - Folding, sorting and degradation** | **272** | **5.8** | **17.6** | **9** | **33.1** | **6** | **L** |  |
| **196** | **Olevolosi-M2** | **Olevolosi-M2** | **1.90** | **HSP20-like chaperones superfamily protein** | **Soltu.DM.06G031870.1** | **Genetic Information Processing - Folding, sorting and degradation** | **238** | **6.2** | **17.6** | **9** | **34.4** | **6** | **L** |  |
| **196** | **Olevolosi-M2** | **Olevolosi-M2** | **1.90** | **Heat shock protein 17.4** | **Soltu.DM.09G009430.1** | **Genetic Information Processing -Folding, sorting and degradation** | **133** | **5.3** | **17.9** | **3** | **16.6** | **3** | **L** |  |
| **203** | **Olevolosi-M2** | **Olevolosi-M2** | **3.64** | **MLP-like protein** | **Soltu.DM.09G027690.1** | **Environmental Information Processing ++** | **439** | **6.0** | **17.3** | **21** | **41.7** | **7** | **L** |  |
| **203** | **Olevolosi-M2** | **Olevolosi-M2** | **3.64** | **MLP-like protein** | **Soltu.DM.09G027720.2** | **Environmental Information Processing ++** | **62** | **5.8** | **17.2** | **4** | **9.7** | **2** | **L** |  |
| **204** | **Olevolosi-M2** | **Olevolosi-M2** | **3.15** | **MLP-like protein** | **Soltu.DM.09G027690.1** | **Environmental Information Processing ++** | **217** | **6.0** | **17.3** | **9** | **14.7** | **3** | **L** |  |
| **204** | **Olevolosi-M2** | **Olevolosi-M2** | **3.15** | **F-box and associated interaction domains-containing protein** | **Soltu.DM.06G021470.1** | **Genetic information processing ++** | **38** | **5.5** | **22.4** | **2** | **12.4** | **2** | **M** |  |
| **208** | **Olevolosi-M2** | **Olevolosi-M2** | **3.19** | **Cruciferin** | **Soltu.DM.03G000650.1** | **Storage protein ++** | **120** | **7.9** | **42.6** | **5** | **2.4** | **2** | **M** |  |
| **208** | **Olevolosi-M2** | **Olevolosi-M2** | **3.19** | **RmlC-like cupins superfamily protein** | **Soltu.DM.09G021450.1** | **Seed storage protein ++** | **101** | **6.1** | **49.9** | **3** | **3.4** | **2** | **M** |  |
| **210** | **Olevolosi-M2** | **Olevolosi-M2** | **4.52** | **Cruciferin** | **Soltu.DM.03G000650.1** | **Storage protein ++** | **108** | **7.9** | **42.6** | **5** | **2.4** | **2** | **M** |  |
| **239** | **Olevolosi-M2** | **Olevolosi-M2** | **2.02** | **RmlC-like cupins superfamily protein** | **Soltu.DM.03G000660.1** | **Seed storage protein ++** | **158** | **9.6** | **14.7** | **9** | **13.8** | **3** | **M** |  |
| **292** | **Olevolosi-M2** | **Olevolosi-M2** | **9.14** | **Cupin family protein** | **Soltu.DM.09G020060.1** | **Seed storage protein ++** | **280** | **9.3** | **53.5** | **33** | **12.0** | **7** | **L** |  |
| **292** | **Olevolosi-M2** | **Olevolosi-M2** | **9.14** | **Cupin family protein** | **Soltu.DM.09G024710.1** | **Seed storage protein ++** | **104** | **5.9** | **42.5** | **5** | **5.6** | **2** | **L** |  |
| **292** | **Olevolosi-M2** | **Olevolosi-M2** | **9.14** | **Cupin family protein** | **Soltu.DM.09G024720.1** | **Seed storage protein ++** | **72** | **8.6** | **65.7** | **2** | **4.8** | **2** | **L** |  |
| **322** | **Olevolosi-M2** | **Olevolosi-M2** | **3.62** | **Cruciferin** | **Soltu.DM.09G026760.1** | **Seed storage protein ++** | **347** | **6.7** | **58.1** | **21** | **11.5** | **6** | **M** |  |
| **328** | **Olevolosi-M2** | **Olevolosi-M2** | **12.80** | **Cruciferin** | **Soltu.DM.09G026760.1** | **Seed storage protein ++** | **246** | **6.7** | **58.1** | **7** | **8.5** | **4** | **M** |  |
| **328** | **Olevolosi-M2** | **Olevolosi-M2** | **12.80** | **RmlC-like cupins superfamily protein** | **Soltu.DM.03G000660.1** | **Seed storage protein ++** | **174** | **9.6** | **14.7** | **11** | **13.8** | **3** | **L** |  |
| **328** | **Olevolosi-M2** | **Olevolosi-M2** | **12.80** | **Cruciferin** | **Soltu.DM.03G000650.1** | **Storage protein ++** | **101** | **7.9** | **42.6** | **4** | **4.7** | **2** | **L** |  |
| **328** | **Olevolosi-M2** | **Olevolosi-M2** | **12.80** | **RmlC-like cupins superfamily protein** | **Soltu.DM.09G021500.1** | **Seed storage protein ++** | **100** | **7.0** | **54.4** | **5** | **5.0** | **3** | **M** |  |
| **328** | **Olevolosi-M2** | **Olevolosi-M2** | **12.80** | **Pyridoxine biosynthesis 1.2** | **Soltu.DM.03G034640.1** | **Metabolism - Metabolism of cofactors and vitamins - Vitamin B6 metabolism** | **92** | **5.2** | **32.7** | **5** | **9.8** | **3** | **L** |  |
| **330** | **Olevolosi-M2** | **Olevolosi-M2** | **1.65** | **Late embryogenesis abundant domain-containing protein / LEA domain-containing protein** | **Soltu.DM.07G020570.1** | **Metabolism ++** | **618** | **5.2** | **56.3** | **23** | **21.4** | **10** | **L** |  |
| **330** | **Olevolosi-M2** | **Olevolosi-M2** | **1.65** | **Sec14p-like phosphatidylinositol transfer family protein** | **Soltu.DM.11G013030.1** | **Biological processes – cell growth and division ++** | **212** | **4.9** | **56.2** | **7** | **6.7** | **4** | **L** |  |
| **333** | **Olevolosi-M2** | **Olevolosi-M2** | **3.39** | **Late embryogenesis abundant domain-containing protein / LEA domain-containing protein** | **Soltu.DM.07G020570.1** | **Metabolism ++** | **666** | **5.2** | **56.3** | **38** | **22.6** | **11** | **L** |  |
| **380** | **Olevolosi-M2** | **Olevolosi-M2** | **5.06** | **RmlC-like cupins superfamily protein** | **Soltu.DM.09G021460.1** | **Seed storage protein ++** | **371** | **7.9** | **53.4** | **26** | **18.1** | **5** | **M** |  |
| **380** | **Olevolosi-M2** | **Olevolosi-M2** | **5.06** | **RmlC-like cupins superfamily protein** | **Soltu.DM.09G021500.1** | **Seed storage protein ++** | **280** | **7.0** | **54.4** | **18** | **12.7** | **5** | **M** |  |
| **382** | **Olevolosi-M2** | **Olevolosi-M2** | **2.73** | **RmlC-like cupins superfamily protein** | **Soltu.DM.09G021460.1** | **Seed storage protein ++** | **333** | **7.9** | **53.4** | **23** | **12.4** | **4** | **M** |  |
| **382** | **Olevolosi-M2** | **Olevolosi-M2** | **2.73** | **RmlC-like cupins superfamily protein** | **Soltu.DM.09G021500.1** | **Seed storage protein ++** | **215** | **7.0** | **54.4** | **12** | **10.8** | **4** | **M** |  |
| **421** | **Olevolosi-M2** | **Olevolosi-M2** | **100.34** | **Phosphoglycerate kinase** | **Soltu.DM.07G028580.1** | **Metabolism – Glycolysis ++** | **1162** | **5.4** | **42.3** | **45** | **53.9** | **19** | **L** |  |
| **421** | **Olevolosi-M2** | **Olevolosi-M2** | **100.34** | **Hydroxysteroid dehydrogenase** | **Soltu.DM.06G021080.1** | **Biological processes – Growth and seed production ++** | **381** | **7.8** | **42.9** | **9** | **21.6** | **7** | **L** |  |
| **421** | **Olevolosi-M2** | **Olevolosi-M2** | **100.34** | **RmlC-like cupins superfamily protein** | **Soltu.DM.09G021450.1** | **Seed storage protein ++** | **90** | **6.1** | **49.9** | **3** | **3.4** | **2** | **L** |  |
| **425** | **Olevolosi-M2** | **Olevolosi-M2** | **2.37** | **Cruciferin** | **Soltu.DM.09G026760.1** | **Seed storage protein ++** | **442** | **6.7** | **58.1** | **38** | **11.5** | **7** | **L** |  |
| **425** | **Olevolosi-M2** | **Olevolosi-M2** | **2.37** | **mitochondrial lipoamide dehydrogenase** | **Soltu.DM.05G023290.1** | **Metabolism - Carbohydrate metabolism - Glycolysis / Gluconeogenesis** | **54** | **7.1** | **52.8** | **2** | **3.6** | **2** | **L** |  |
| **426** | **Olevolosi-M2** | **Olevolosi-M2** | **4.21** | **Protein of unknown function (DUF1264)** | **Soltu.DM.09G031240.1** | **Unknown ++** | **641** | **5.7** | **26.9** | **55** | **41.2** | **10** | **L** |  |
| **426** | **Olevolosi-M2** | **Olevolosi-M2** | **4.21** | **RmlC-like cupins superfamily protein** | **Soltu.DM.09G021450.1** | **Seed storage protein ++** | **52** | **6.1** | **49.9** | **2** | **3.4** | **2** | **M** |  |
| **491** | **Olevolosi-M2** | **Olevolosi-M2** | **2.65** | **Cupin family protein** | **Soltu.DM.09G024720.1** | **Seed storage protein ++** | **207** | **8.6** | **65.7** | **20** | **7.6** | **6** | **M** |  |
| **491** | **Olevolosi-M2** | **Olevolosi-M2** | **2.65** | **Cupin family protein** | **Soltu.DM.09G024710.1** | **Seed storage protein ++** | **136** | **5.9** | **42.5** | **6** | **5.6** | **2** | **L** |  |
| **510** | **Olevolosi-M2** | **Olevolosi-M2** | **4.75** | **Glycosyl hydrolase family protein** | **Soltu.DM.06G029150.1** | **Metabolism - Hydrolases –Glycosylases- [EC 3.2.1.21] ++** | **276** | **5.7** | **55.9** | **11** | **10.9** | **5** | **L** |  |
| **510** | **Olevolosi-M2** | **Olevolosi-M2** | **4.75** | **Glycosyl hydrolase family protein** | **Soltu.DM.06G029150.2** | **Metabolism - Hydrolases –Glycosylases- [EC 3.2.1.21] ++** | **268** | **6.9** | **66.0** | **14** | **9.2** | **5** | **L** |  |
| **510** | **Olevolosi-M2** | **Olevolosi-M2** | **4.75** | **GTP binding Elongation factor Tu family protein** | **Soltu.DM.06G005560.1** | **Genetic Information Processing - Translation - RNA transport and biogenesis** | **184** | **9.4** | **35.7** | **6** | **12.0** | **4** | **L** |  |
| **510** | **Olevolosi-M2** | **Olevolosi-M2** | **4.75** | **Glycosyl hydrolase family protein** | **Soltu.DM.06G029160.1** | **Metabolism - Hydrolases –Glycosylases- [EC 3.2.1.21] ++** | **173** | **8.5** | **68.7** | **9** | **5.9** | **3** | **L** |  |
| **510** | **Olevolosi-M2** | **Olevolosi-M2** | **4.75** | **RmlC-like cupins superfamily protein** | **Soltu.DM.09G021500.1** | **Seed storage protein ++** | **89** | **7.0** | **54.4** | **4** | **6.2** | **2** | **L** |  |
| **512** | **Olevolosi-M2** | **Olevolosi-M2** | **3.56** | **Glycosyl hydrolase family protein** | **Soltu.DM.06G029150.1** | **Metabolism - Hydrolases –Glycosylases- [EC 3.2.1.21] ++** | **439** | **5.7** | **55.9** | **20** | **14.3** | **8** | **L** |  |
| **512** | **Olevolosi-M2** | **Olevolosi-M2** | **3.56** | **Glycosyl hydrolase family protein** | **Soltu.DM.06G029150.2** | **Metabolism - Hydrolases –Glycosylases- [EC 3.2.1.21] ++** | **392** | **6.9** | **66.0** | **20** | **10.9** | **7** | **L** |  |
| **512** | **Olevolosi-M2** | **Olevolosi-M2** | **3.56** | **Glycosyl hydrolase family protein** | **Soltu.DM.06G029160.1** | **Metabolism - Hydrolases –Glycosylases- [EC 3.2.1.21] ++** | **286** | **8.5** | **68.7** | **14** | **8.0** | **5** | **L** |  |
| **512** | **Olevolosi-M2** | **Olevolosi-M2** | **3.56** | **Glycosyl hydrolase family protein** | **Soltu.DM.11G024490.2** | **Metabolism - Hydrolases –Glycosylases- [EC 3.2.1.21] ++** | **121** | **9.0** | **69.6** | **7** | **4.4** | **3** | **L** |  |
| **512** | **Olevolosi-M2** | **Olevolosi-M2** | **3.56** | **GTP binding Elongation factor Tu family protein** | **Soltu.DM.06G005580.1** | **Genetic Information Processing - Translation - RNA transport and biogenesis** | **107** | **9.8** | **49.2** | **3** | **3.8** | **2** | **L** |  |

**A** = SpotID as defined by the Delta2D software from DECODON on the master gel from the 2D PAGE gels. Corresponding spots of all gels are labelled with the same ID.

**B** = Accession for which the proteins in the row were identified.

**C** = Proteins were identified from spots picked from the following 2D PAGE gel. Ole stands for Olevolosi, Abu stands for Abuku and Acc33 stands for Accession 33, M1 = maturity state 1 (green berries), M2 = maturity state 2 (purple berries).

**D** = Regulation of a spot according to the comparison between groups. Regulation is given as the ratio between spot abundance (M2 / M1).

**E** = Functional classification mainly following the KEGG Pathway Database (++ = if no classification was automatically annotated, the proteins were manually classified).

**F** = The protein score obtained via the MASCOT search algorithm (www.matrixscience.com) against a potato protein database, which was based upon the sequences from *Solanum tuberosum* group Phureja DM1-3 v 6.1, which was completely sequenced by the Potato Genome Consortium in 2020.

**G** = Calculated PI obtained via the MASCOT search algorithm (www.matrixscience.com) against a potato protein database.

**H** = Calculated MW obtained via the MASCOT search algorithm (www.matrixscience.com) against a potato protein database.

**I** = Number of peptides matched to the protein through the database search.

**J** = Sequence coverage in %.

**K** = Unique peptides matched to the sequence. Only proteins with at least two unique peptide were considered true hits.

**L** = Molecular weight (MW) in gel as compared to the theoretically expected MW.

**M**: MW in gel corresponding to the theoretically expected MW ± 15 kDa

**S**: MW in gel lower than theoretically expected

**L**: MW in gel larger than theoretically expected

**M** = Mean relative spot volume obtained according to three gels of M1 seeds or M2 seeds illustrated by graphs. The first bar (orange) represents the mean normalized spot volume in the gels of M1 seeds of Accession 33. The second bar (light orange) represents the mean normalized spot volume in the gels of the M2 seeds from Accession 33. The third bar (green) stands for the mean normalized spot volume in the gels of the M1 seeds of Abuku 1. The fourth bar (light green) represents the mean normalized spot volume in the gels of the M2 seeds of Abuku 1. The fifth bar (purple) represents the mean normalized spot volume in the gels of the M1 seeds from Olevolosi. The sixth bar (light purple) stands for the mean normalized spot volume in the gels of the M2 seeds of Olevolosi.

# Reference

1. Kanehisa, M., Furumichi, M., Sato, Y., Kawashima, M. & Ishiguro-Watanabe, M. KEGG for taxonomy-based analysis of pathways and genomes. *Nucleic Acids Res.* **51**, D587–D592 (2023).
