# Supplementary material for: Effect of berry maturity stages on the germination and protein constituents of African nightshade (Solanum scabrum) seeds
Source: Sci Rep. 2024 Dec 16;14:30482. doi: 10.1038/s41598-024-80312-6 (PMC11649806; doi:10.1038/s41598-024-80312-6)
Supplement: Supplementary file 7 — Supplementary Material 7 [file 41598_2024_80312_MOESM7_ESM.docx]

**Supplementary Table S7: Germination percentage, Mean Germination Time (MGT) and Mean Germination Rate (MGR) of nine African nightshade (*Solanum scabrum*) seed accessions harvested at two maturity stages (M1 and M2).** M1 = the mature green stage; M2 = the ripe purple stage. The MGT was calculated using the formula, $MGT=(\sum(ni\times ti))/(\sum ni)$, where *ni​* = number of seeds germinated at time and *ti*​ = time to germination (in days). MGR is the reciprocal of the MGT.

| **Accession** | **M1** | | **M2** | | **M1** | | **M2** | |
| --- | --- | --- | --- | --- | --- | --- | --- | --- |
|  | Mean Germination Time (MGT) (days) | Mean Germination Rate (MGR) (days) | Mean Germination time (MGT) (days) | Mean Germination Rate (MGR) (days) | Mean Germination Time (MGT) (days) | Mean Germination Rate (MGR) (days) | Mean Germination Time (MGT) (days) | Mean Germination Rate (MGR) (days) |
| Olevolosi | 6.48±0.156 | 0.154 ±0.024 | 5.64±0.158 | 0.177 ±0.028 | 6.36 ±0.057 | 0.157±0.009 | 5.58±0.100 | 0.179±0.018 |
| Acc 33 | 5.38±0.268 | 0.186±0.032 | 4.68±0.197 | 0.214±0.042 | 5.62±0.320 | 0.178±0.057 | 4.46±0.143 | 0.224±0.032 |
| Abuku 1 | 5.47±0.077 | 0.183±0.014 | 4.93±0.039 | 0.203±0.008 | 5.22±0.240 | 0.192±0.046 | 4.75±0.195 | 0.211±0.041 |
| Abuku 2 | 6.02±0.223 | 0.166±0.037 | 4.78±0.220 | 0.209±0.046 | 6.44±0.219 | 0.155±0.034 | 4.72±0.264 | 0.212±0.056 |
| Acc1 | 5.92±0.101 | 0.169±0.017 | 5.18±0.161 | 0.193±0.031 | 6.07±0.237 | 0.165±0.039 | 4.98±0.224 | 0.201±0.045 |
| Acc 18 | 5.96±0.131 | 0.168±0.022 | 5.06±0.278 | 0.198±0.055 | 6.15±0.049 | 0.163±0.008 | 5.22±0.084 | 0.192±0.016 |
| Acc3 | 5.57±0.050 | 0.180±0.009 | 4.84±0.203 | 0.207±0.042 | 5.6±0.146 | 0.179±0.026 | 4.96±0.253 | 0.202±0.051 |
| Acc7 | 6.24±0.256 | 0.160±0.041 | 4.96±0.188 | 0.202±0.038 | 6.36±0.280 | 0.157±0.044 | 5.05±0.222 | 0.198±0.044 |
| SS 40 | 6.37±0.083 | 0.157±0.013 | 5.26±0.189 | 0.190±0.036 | 6.28±0.151 | 0.157±0.024 | 4.89±0.186 | 0.204±0.038 |
